# Therapeutic functions of medical implants from various material categories with integrated biomacromolecular systems

**DOI:** 10.3389/fbioe.2024.1509397

**Published:** 2025-01-10

**Authors:** Guilherme Bedeschi Calais, Guilherme Domingos Garcia, Celso Fidelis de Moura Júnior, José Diego Magalhães Soares, Liliane Maria Ferrareso Lona, Marisa Masumi Beppu, Jacobo Hernandez-Montelongo, João Batista Maia Rocha Neto

**Affiliations:** ^1^ Universidade Estadual de Campinas (UNICAMP), School of Chemical Engineering, Department of Materials Engineering and Bioprocesses, Campinas, Brazil; ^2^ Federal University of Alagoas, Center of Technology, Maceió, Brazil; ^3^ Federal Institute of Alagoas (IFAL), Chemistry Coordination Office (Campus Maceió), Maceió, Brazil; ^4^ Universidad Católica de Temuco, Department of Mathematical and Physical Sciences, Bioproducts and Advanced Materials Research Center (BioMA), Temuco, Chile; ^5^ Universidad de Guadalajara, Department of Translational Bioengineering, Guadalajara, Mexico

**Keywords:** medical implant, therapeutic, metal, polymer, biopolymer, ceramic, composite

## Abstract

Medical implants are designed to replace missing parts or improve body functions and must be capable of providing structural support or therapeutic intervention for a medical condition. Advances in materials science have enabled the development of devices made from metals, polymers, bioceramics, and composites, each with its specific advantages and limitations. This review analyzes the incorporation of biopolymers, proteins, and other biomacromolecules into implants, focusing on their role in biological integration and therapeutic functions. It synthesizes advancements in surface modification, discusses biomacromolecules as carriers for controlled drug release, and explores the application of nanoceramics and composites to improve osseointegration and tissue regeneration. Biomacromolecule systems are capable of interacting with device components and therapeutic agents - such as growth factors (GFs), antibiotics, and nanoceramics - allowing control over substance release. Incorporating therapeutic agents into these systems enables localized treatments for tissue regeneration, osseointegration, post-surgery infection control, and disease and pre-existing conditions. The review highlights these materials’ therapeutic advantages and customization opportunities, by covering mechanical and biological perspectives. Developing composites and hybrid drug delivery systems align with recent efforts in interdisciplinary personalized medicine and implant innovations. For instance, a trend was observed for integrating inorganic (especially nanoceramics, e.g., hydroxyapatite) and organic phases in composites for better implant interaction with biological tissues and faster recovery. This article supports understanding how integrating these materials can create more personalized, functional, durable, and biocompatible implant devices.

## Graphical Abstract

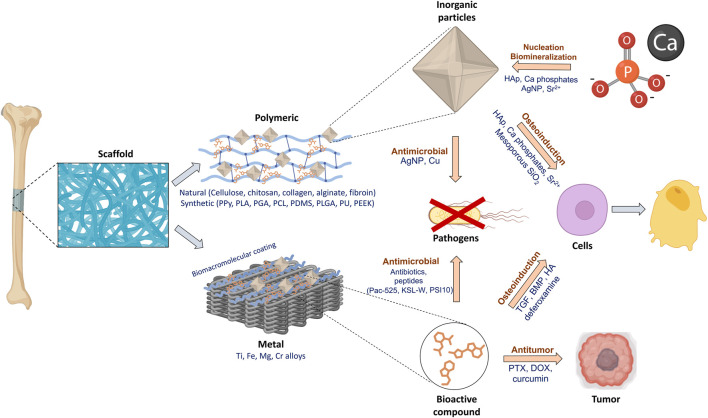

## 1 Introduction

### 1.1 Medical implants

Biomedical implants are used to extend the functionality of important body systems to monitor, prevent failure, or replace missing elements, being partly or totally introduced into the body permanently or temporarily (Teo et al., 2016; Mezher and AlAbbas, 2022). The U.S.A. Food and Drug Administration (FDA) also considers medical implants “devices or tissues that are placed inside or on the surface of the body” (Implants and Prosthetics, 2019), some of them intending to replace missing body parts (Ghasemi-Mobarakeh et al., 2019) and others to perform different functionalities such as treatment and diagnosis (Lee et al., 2021; Harman, 2020) by delivering medication, monitoring body functions, or offering structural support to organs and tissues (Implants and Prosthetics, 2019).

Common implantable devices include catheters, artificial knees or eye lenses, coronary stents, plate and screw fixation for fractures, silicone breasts, and uterine contraceptive devices. Modern devices can also present electronic properties such as *in vivo* sensors and actuators like brain stimulators, cochlear implants, insulin pumps, cardiac defibrillators, wireless capsule endoscopes, and gastric stimulators, with some of them being able to transmit wireless signals to outside the body through wireless body area networks (Mezher and AlAbbas, 2022; Li et al., 2015a; Mezher et al., 2020). These devices can be produced with different categories of materials, such as metals and alloys, synthetic polymers, and biopolymers (Patel et al., 2016), bioceramics and bioglasses or composites, depending on the applied site and desired functional properties (Filip et al., 2022), each possessing its particle advantages and limitations. However, many limitations of the implant matrices can be overcome, and additional properties can be obtained by combining them with other materials (Fujihara et al., 2004a), such as systems composed of biomacromolecules.

### 1.2 Biomacromolecule systems

Biomacromolecules such as proteins, nucleic acids, and polysaccharides produced by organisms (Nagel et al., 1992) can comprise compelling systems for loading and releasing therapeutic agents (Lopes et al., 2022). Among the employed biomacromolecules, polysaccharides are especially attractive for composing drug-loading systems because while they offer a significant versatility of uses and behaviors, they also present the differential of being biocompatible (Barclay et al., 2019). The functional groups of organic molecules take part in the most diverse types of interactions, whether ensuring the stability of the coatings they may constitute or controlling the loading and release of active ingredients and being able to participate either in the constitution of the device matrix (Rebelo et al., 2017) or in surface coatings (Richardson et al., 1979; Eftekhar Ashtiani et al., 2021; López-Valverde et al., 2022a; Escobar et al., 2023a) capable of retaining the desired bioactive components.

### 1.3 Therapeutic agents

Therapeutic agents consist of bioactive molecules and substances used to treat diseases or disorders, which aim to cure a disease or condition, alleviate its symptoms, or enhance the patient’s quality of life (Faccio et al., 2022; Oprea and Hasselgren, 2017). In the case of implants, they are usually employed for improved osseointegration, tissue regeneration, antibacterial and anti-inflammatory properties, or controlled drug delivery. They are divided into several categories regarding the treated condition (Food and Drug Administration FDA, 2018) and are obtained through biological or synthetic paths (Kharkar et al., 2017). These active compounds may be used to prevent risks associated with implant insertion and permanence (Hu et al., 2023a; Braem et al., 2023), but can also be employed in cases where the implant itself is used as a drug vessel to treat a pre-existing illness or condition (Dash and Cudworth, 1998). For instance, implant surgery may impose several risks and reactions such as inflammations, infections, calcifications, necrosis, fibrosis, swelling, inadequate healing, and severe immune responses (Lopes et al., 2022; Hu et al., 2023b; Major et al., 2015).

The creation of an implantable drug delivery system must take into account its compatibility with the human body. They are divided into biodegradable or nonbiodegradable implants (e.g., pump systems) and consist of monolithic or reservoir systems (Dash and Cudworth, 1998). Any used components must be non-carcinogenic, hypoallergenic, and mechanically stable at the implant site. Usually, the material should not be altered by the environment of the implantation site, and it must not cause any inflammatory or thrombogenic response, or be chemically or physically affected by sterilization processes. It is desirable that it can be easily removed or bioabsorbable after its therapeutic duration (Patel et al., 2016; Dash and Cudworth, 1998; Danckwerts and Fassihi, 1991).

## 2 Implantable materials

### 2.1 Metals

#### 2.1.1 Background

Metals have long been used in orthopedic, dental, and cardiovascular applications, such as in stents, joint replacements, bone plates, spinal screws, cardiac pacemakers, and dental implants (Davis et al., 2022; de Andrade et al., 2022; Copes et al., 2024). Among these, absorbable metal alloys have made a significant impact, revolutionizing biomedical implants by offering structural support to damaged tissues while interacting positively with the surrounding biological environment (Loffredo et al., 2022; Sotoudeh Bagha et al., 2023). Biodegradable metal alloys represent a novel approach in medical applications, including advanced implants like electronic sensors and drug delivery devices. Compared to polymer-based alternatives, these metals provide superior strength and performance, particularly in stents and bone implants (Li et al., 2022). Additionally, applying biomolecules to the surface of implants can accelerate osseointegration, enhance mobility, and help prevent inflammation, infections, and mechanical complications (Dong et al., 2020).

#### 2.1.2 Composition

Materials commonly used in permanent metallic implants include titanium alloys, stainless steel alloys, cobalt-chromium alloys, nitinol (Ni-Ti alloy), and tantalum alloys (Copes et al., 2024). However, biodegradable metals such as magnesium (Mg), iron (Fe), and zinc (Zn) have been proposed for the development of stents that naturally degrade after vessel recovery, as also for bone fixture screws, nails, plates, pins, and scaffolds, thus mitigating long-term complications (Sotoudeh Bagha et al., 2023; Catanio Bortolan et al., 2023; Prasadh et al., 2022). Each of these metals offers unique advantages and challenges when used in implants. Titanium (Ti) and its alloys are commonly used in pacemaker housings, orthopedic implants, dental implants, and surgical instruments (Resnik et al., 2020). Commercially pure titanium offers high strength, excellent biocompatibility, and corrosion resistance, while titanium alloys have superior mechanical properties. Despite their benefits, concerns arise regarding the release of vanadium (Va) and aluminum (Al), which may pose toxicity risks (Zafar et al., 2020).

SS alloys are widely employed in bone implants, stents, dental plates, nails, screws, and surgical instruments due to their low manufacturing cost, ease of machining, good fatigue properties, and biocompatibility (Resnik et al., 2020). However, they may corrode in long-term applications and can cause allergic or inflammatory reactions due to the presence of nickel (Ni) and chromium (Cr) (Bandyopadhyay et al., 2023).

Cobalt-chromium alloys are used in surgical instruments, as well as orthopedic, cardiac, and intracranial implants due to their excellent resistance to fatigue, long-term corrosion, and superior moldability. However, concerns about potential toxicity arise not only from the presence of nickel but also from the main alloy components, cobalt (Co) and chromium (Cr) (Kovochich et al., 2021).

Magnesium and its alloys are used in cardiovascular stents, offering superior strength, ductility, and degradability compared to other biodegradable metals like zinc and iron, both of which are naturally found in the human body and are non-toxic (Loffredo et al., 2022; Gambaro et al., 2021). These biodegradable metal alloys, also known as bioabsorbable materials, represent a promising new class of materials with applications in bone fixation devices such as screws, nails, plates, pins, and scaffolds, holding the potential to revolutionize advanced surgical treatments (Ryu et al., 2021).

### 2.2 Synthetic polymers

#### 2.2.1 Background

Polymers have gained broad attention due to their distinctive combination of chemical versatility, biocompatibility, and ease of modification to meet specific therapeutic needs (Song et al., 2018; Sun et al., 2020). Polymers are utilized in medical implants either as core components of the device or as substrates and protective packaging (Qin et al., 2014). They are often selected for the attractive characteristics they present, such as ease of manufacturing and the adaptability of their mechanical, electrical, chemical, and thermal properties (Ershad-Langroudi et al., 2024a). Polymers can also be combined with other materials to form composites, enhancing their properties and expanding their potential applications in implants (Teo et al., 2016). A significant advantage of their use in medical implants is their ability to incorporate therapeutic biomolecules, such as proteins, peptides, and nucleic acids. This capability allows the implants to serve dual purposes: providing structural support and performing active therapeutic functions like promoting healing, aiding tissue regeneration, or preventing infections (Zhang et al., 2019a).

The development of responsive or “smart” polymers has further expanded the therapeutic potential of medical implants. These polymers can respond to external stimuli, such as pH changes, light, ionic strength, or electric and magnetic fields, by undergoing physical or chemical transformations. Such responsiveness is valuable for mimicking complex biological functions (Li et al., 2015b; Yuan et al., 2022; Liu et al., 2020a). Recent advancements in polymer-based drug delivery systems have also been significant, particularly in improving drug targeting and release control precision. These systems range from non-biodegradable diffusion-controlled membranes to biodegradable systems that combine diffusion with matrix degradation, demonstrating the potential to enhance therapeutic outcomes while reducing adverse effects (Liu et al., 2020b).

#### 2.2.2 Composition

**FIGURE 1:**
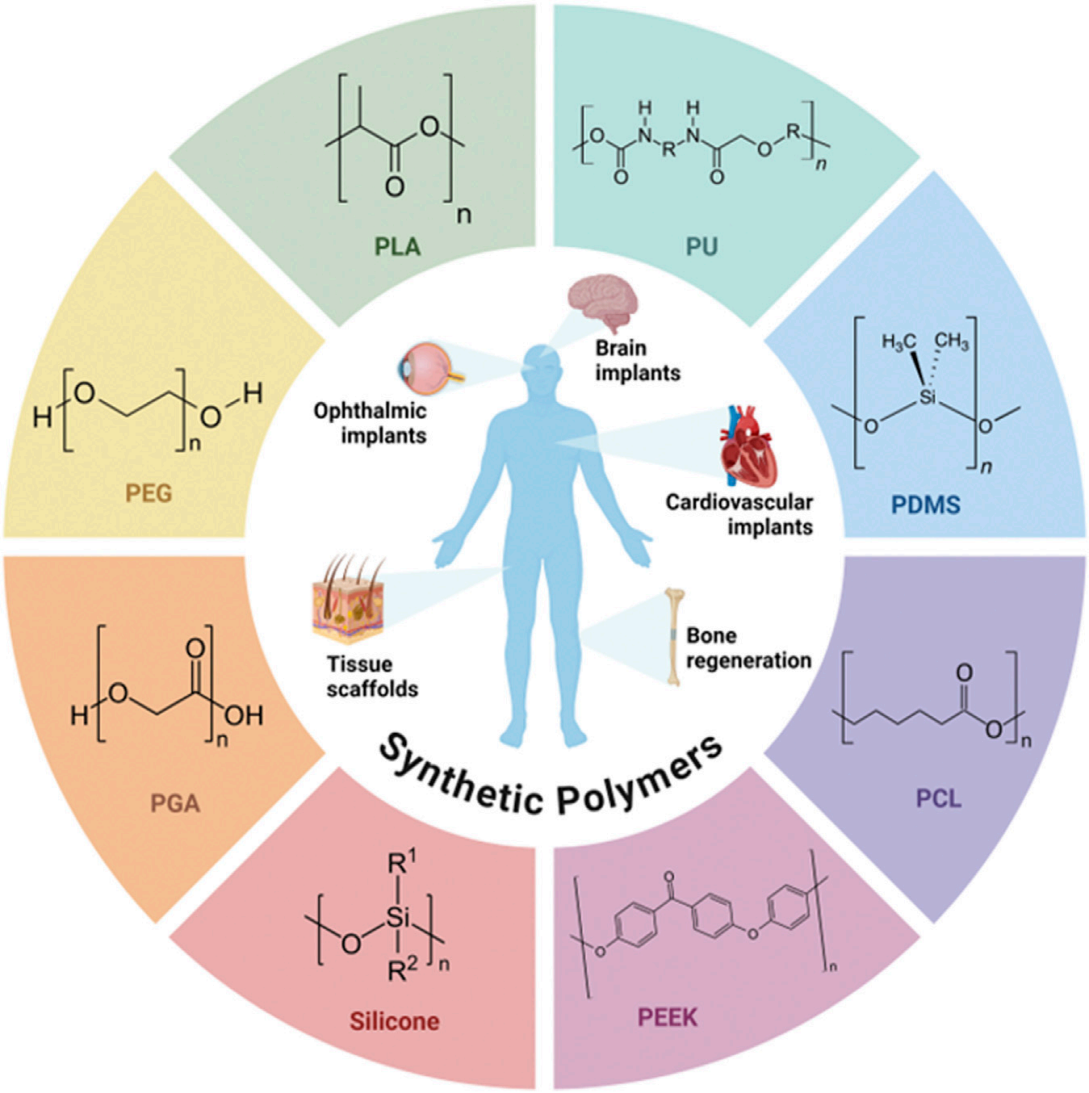
Chemical structure of synthetic polymers used in implants.

Synthetic polymers like polyethylene (PE), polyethylene glycol (PEG), polyurethane (PU), poly(ε-caprolactone) (PCL), polylactic acid (PLA), polyglycolic acid (PGA), poly(lactic-co-glycolic acid) (PLGA), silicone, poly(ethylene-vinyl acetate), polydimethylsiloxane (PDMS), and polyether ether ketone (PEEK) offer greater design flexibility when compared to their natural counterpart. These materials provide customizable mechanical and chemical resistance, controlled biodegradation rates, and surface functionalization, making them suitable for a wide range of applications, such as controlled drug delivery, bone regeneration, and vascular stents (Hu et al., 2018; Hua et al., 2022; Shen et al., 2022; Xia et al., 2022). Figure 1 presents the chemical structure of the synthetic polymers used in implants, highlighting the different compositions and characteristics that grant these materials their functional properties.

Synthetic polymers have become indispensable in modern medicine, particularly in the realm of medical implants. Their versatility and customizable nature make them highly suitable for meeting the rigorous demands of clinical applications (Terzopoulou et al., 2022; Al-Shalawi et al., 2023; Prete et al., 2023). For instance, polymers like PE and PU are chosen for their robust mechanical properties, which can be adjusted to provide the necessary strength in devices supporting physical loads, such as joint prostheses and orthopedic implants (García-Rey and García-Cimbrelo, 2010; Chakrabarty et al., 2015; Ong et al., 2015; Cooke et al., 2020). Additionally, polymers such as PLA and PGA offer advantages in applications requiring biodegradability, as they naturally decompose in the body, minimizing the need for additional surgery to remove the implant (Low et al., 2020; Khouri et al., 2024).

Surface functionalization of synthetic polymers is a valuable technique that enhances biocompatibility and integration with adjacent tissues. Coating implants with biomolecules, such as peptides or proteins, can improve cell adhesion and immune system response (Badv et al., 2020; Jurczak et al., 2020). Furthermore, grafting techniques allow specific functional groups to be added to the surface of polymers, resulting in desirable properties such as resistance to thrombosis in vascular stents or support for cell proliferation in scaffolds for tissue engineering (Fathi-Karkan et al., 2022).

Another significant advantage of synthetic polymers is the potential for controlled drug release. The incorporation of drugs into polymer matrices allows for sustained and controlled release, increasing therapeutic efficacy and reducing side effects. For instance, controlled-release devices based on PLGA have been frequently studied for the treatment of cardiovascular diseases (Kumari et al., 2010; de Souza Ferreira et al., 2023; Lü et al., 2009; Fishbein et al., 2012; Alsaab et al., 2022; Mir et al., 2017), where the gradual release of antiplatelet drugs can prevent restenosis after stent insertion.

The production of implants using synthetic polymers has evolved with the advent of 3D printing, enabling the fabrication of customized devices with complex structures. This technology is particularly useful in creating scaffolds that mimic the architecture of natural tissue, promoting more efficient integration and effective tissue regeneration (Paxton et al., 2023; Moroni et al., 2008; Kim et al., 2012; Gaharwar et al., 2020). Personalization of implants also allows them to be adapted to the specific anatomical characteristics of each patient, thereby improving clinical outcomes.

### 2.3 Biopolymers

#### 2.3.1 Background

Biopolymers are organic substances found in natural sources, being the most abundant macromolecules. They can be obtained from plants or microbial systems, or be chemically produced from basic biological systems (Rebelo et al., 2017; Baranwal et al., 2022). They are often the best alternatives for various applications in the medical field due to certain advantages, such as being biologically renewable, biodegradable, and, most importantly, biocompatible. However, they also present some disadvantages, including low stability, complex structures, wide composition variability, and lower mechanical properties (Rebelo et al., 2017; Baranwal et al., 2022; Bibire et al., 2022). They can be used in various applications, primarily selected for producing and enhancing medical devices, such as temporary prostheses, scaffolds, applications in tissue engineering, and controlled release of therapeutic agents (Escobar et al., 2023b; Bansal et al., 2022). This is possible due to the similarities of biopolymers to biological macromolecules, promoting cell growth and adhesion. Due to the biodegradability of these materials, implants can be gradually replaced by extracellular matrices without causing harmful reactions to the patient (Bansal et al., 2022; Biswas et al., 2022). Moreover, the development of drug delivery systems by integration of biopolymers has become an essential technique for improving the efficacy of bioactive compounds in disease treatment (Baranwal et al., 2022; Ruso and Messina, 2017).

#### 2.3.2 Composition

Natural polymers are commonly chosen for their excellent biocompatibility, enabling seamless integration with biological tissues (Sergi et al., 2020; Zhang H. et al., 2024). These materials are frequently used in biodegradable sutures, grafts, and scaffolds—three-dimensional structures that promote tissue regeneration (Islam et al., 2020; Notario-Pérez et al., 2022; Vach Agocsova et al., 2023). They are commonly used in medicine due to their versatility, and applying these biomaterials in tissue engineering has become an increasingly attractive approach because of their ability to repair and regenerate biological tissues. One of the main requirements for biopolymer scaffolds in tissue engineering is to simulate the extracellular matrix, enabling them to support the structure and biochemistry of the area (Bibire et al., 2022; Biswas et al., 2022). Moreover, they can be modified to enhance properties such as cell adhesion and proliferation, improving the scaffold’s interaction with the surrounding environment and reconstructing the original tissue structure. The most commonly used biopolymers in surgical implants include polysaccharides such as cellulose, chitosan, alginate, starch, and hyaluronic acid; proteins such as collagen, gelatin, and silk fibroin; and polyesters like polylactic acid derivatives (Rebelo et al., 2017; Bibire et al., 2022; Bansal et al., 2022) (Table 3).

### 2.4 Bioceramics and bioglasses

#### 2.4.1 Background

The need for durable, long-lasting implants further raises the demand for materials with increasing life expectancy, such as ceramics. Ceramics are ideal for bone replacement for joint implants due to their excellent properties of biocompatibility, high hardness, and wear resistance that aim to minimize clinical wear and the risk of debris-induced osteolysis (Filip et al., 2022). These materials are attractive due to their large similarities with human body systems (biomimetics) that are also constituted of bioceramics such as bone, teeth, and other calcified tissues (Shanmugam and Sahadevan, 2018). Moreover, all-ceramic implants have attracted some attention in dentistry as an alternative to traditional titanium-based implants due to their better esthetic appeal when compared to metallic materials (Sherman et al., 2008).

#### 2.4.2 Composition

Ceramic materials are inorganic, nonmetallic materials composed of metal, nonmetal, or metalloid atoms held by ionic or covalent bonds (Desante et al., 2023; Sudha et al., 2018). Traditional ceramics include clay, porcelain, and the minerals feldspar, silica, calcite, and nepheline, whereas the most commonly used bioceramic compounds in biomedical applications include metallic oxides (e.g., ZrO_2_, TiO_2_), silicates, carbides, selenides, refractory hydrides, and sulfides and also carbon structures, such as diamond and graphite (Shanmugam and Sahadevan, 2018). Bioceramics can be classified into three types/generations: bioinert (e.g., zirconia, alumina), bioactive (e.g., bioactive glass, hydroxyapatite), and biodegradable (e.g., calcium phosphates and sulfates) (Filip et al., 2022; Shanmugam and Sahadevan, 2018), while zirconia (ZrO_2_) implants are the leader among all-ceramic dental implants. Zirconia has been used as a matrix for inlays, onlays, single crowns, fixed partial bridges, esthetic orthodontic brackets, endodontic posts, and implant fixtures. Failures in these ZrO_2_ devices are usually associated with wrong operator techniques, defects during production, and unbalanced lateral loading (Prakash et al., 2021). Bioceramic components can also be applied pure or to form composite materials in a great number of applications, such as orthopedic, dental, and maxillary prosthetics, otolaryngologic, artificial tendons and ligaments, or coatings, for example. Shanmugam and Sahadevan reviewed several of these applications and the most adequate bioceramic materials for each one (Shanmugam and Sahadevan, 2018). Bioceramic scaffolds can be fabricated by methods such as gas foaming, soluble or volatile porogen processing, phase-mixing, free-form fabrication such as stereolithography, and template coating and casting (Shanmugam and Sahadevan, 2018).

Silica is present in fillers of composite resins or glass ionomer cement, and alongside hydroxyapatite, it has formed bioactive ceramic materials in the form of bioglasses, also capable of mimicking bone material and stimulating growth. Their advantage to HAp is the ability to bond with both hard and soft tissues, while the latter can only connect to hard tissues. Bioglasses are available in multiple formats and can be molded into any form. They are used to fill periodontal osseous defects, augmentation of the atrophic ridge, remineralization for dentinal hypersensitivity, promote antibacterial activity, carrier for drug-delivery when pure or associated with other components and in composite scaffolds for bone tissue engineering (Krishnan and Lakshmi, 2013).

In the class of nanoceramics - and among the bioactive ceramics - hydroxyapatite is largely employed due to its outstanding biocompatibility and osteoconductivity. However, due to the intrinsic brittleness of this class of material, they are not usually applied purely as a coating of traditional metals but in the form of nanostructured composites - usually polymer/ceramic composites (Shanmugam and Sahadevan, 2018). For instance, there are methods available for even 3D-printing polymer-ceramic composites using commercial fused deposition modeling 3D printers from a composed filament (Podgórski et al., 2023).

### 2.5 Composites and hybrid materials

#### 2.5.1 Background

The combination of materials with different physical-chemical properties in the form of layers or phases dispersed in a matrix makes them composite materials (Mattox, 2010). Considering the example of metallic implants, although titanium devices are widely used and successful, some disadvantages are associated with them, such as the possibility of peri-implantitis, prolonged healing time, immune reactions, and inadequate stress distribution. On the other hand, composite materials may present several advantages, including better biocompatibility, faster osseointegration, compatible modulus of elasticity, reduced susceptibility to corrosion, and better esthetics, making them a promising alternative to titanium implants (Roi et al., 2024). For instance, Fujihara et al. (2004b) reported a carbon fiber-reinforced polyether ether ketone composite (CF-PEEK) composite with both a mechanical strength - essential for load-bearing applications - and a fatigue resistance - related to a suitable application for dynamic environments such as those found in the human body - comparable to conventional metallic type devices. The application of biocomposite materials in the field of biomedical engineering ranges from bone regeneration and orthopedic/dental implants to wound healing and tissue engineering. Bones, for instance, are natural composites formed by hydroxyapatite (a ceramic material) associated with fibers of collagen (polymeric material) (Valente et al., 2020).

#### 2.5.2 Composition

The possibilities for composite materials are extensive, and a common tendency of composite bone scaffolds, for example, is using both organic and inorganic elements combined into systems to better mimic natural bone tissue (Valente et al., 2020). The organic components provide flexibility and enhance biocompatibility, while inorganic components contribute with strength and rigidity for weight-bearing applications (Zarrintaj et al., 2023). Vazquez-Silva et al. (2022) reviewed the most recent (2007–2020) composite and hybrid materials applications in implants, while Kazimierczak and Przekora (2020) listed the most commonly employed organic and inorganic components, some of which are reproduced hereafter. In the case of structural composite materials, for example, they are formed by a reinforcing phase and a matrix phase. The first phase, responsible for strength and stiffness, is typically a graphite, glass, ceramic, or polymer fiber, and the second phase, responsible for binding, is typically a polymer but may also be ceramic or metal (Shanmugam and Sahadevan, 2018). Organic components can be obtained from natural polymers such as chitosan, collagen, fibroin, alginate, carrageenan, and hyaluronic acid (HA) and synthetic polymers like polylactic acid (PLA), polycaprolactone (PCL), PGA, polyether ether ketone (PEEK), and polypropylene fumarate. The inorganic components usually include metals and ceramics, including hydroxyapatite (HAp), calcium phosphate, bioglass (SiO_2_/P_2_O_5_/CaO/MgO/Na_2_O), and carbon nanotubes, which contribute to the mechanical stability of the scaffold (Kazimierczak and Przekora, 2020). Their fabrication ranges from very simple techniques such as hand modeling to sophisticated techniques with complex models, 3D-printing, or deposition methods (Knight and Curliss, 2003).

Saad et al. (2018) reviewed the uses of composite polymers in orthopedic implants, whereas Zarrintaj et al. (2023) focused on biopolymers associated with calcium phosphates. Among the developed implantable polymeric composites are those produced with PEEK associated with glass and carbon fibers (Li et al., 2019a), hydroxyapatite (Zheng et al., 2022), or different biomolecules (Zhang et al., 2019b). Other composites include propylene fumarate scaffolds with hydroxyapatite (Vazquez-Silva et al., 2022; Trachtenberg et al., 2017), PLA-HAp (Vazquez-Silva et al., 2022; Wu et al., 2020), or resins such as bisphenol A-glycidyl methacrylate or triethylene glycol dimethacrylate filled either with γ-methacryloxy propyl trimethoxy silane (Hosseinalipour et al., 2010) or bioactive glasses (Abdulmajeed et al., 2014). Among the biopolymeric composites are collagen-hydroxyapatite systems (Wahl and Czernuszka, 2006), methacrylate hyaluronic acid with titanium oxide (TiO_2_) (Liu et al., 2018), and chitosan associated with calcium phosphate (Di Martino et al., 2005) or hydroxyapatite (Buranapanitkit et al., 2004; Kurtz and Devine, 2007), among many others. Vazquez-Silva et al. (2022) listed several metal-composite devices and reported the impact of the combinations on structural and biologic properties (especially osteogenesis and biocompatibility). They referenced materials such as Ti-HAp/SiHAp/SrHAp (Mumith et al., 2020), SS-HAp (Alkaron et al., 2024), Fe-Calcium silicate (Wang et al., 2017), Mg/Al/Zn alloys-modified HAp (Razavi et al., 2020), Ti-nanostructured glass ceramic (Kazimierczak and Przekora, 2020). Other hybrid inorganic materials include carbon nanotubes-HAp (Lawton et al., 2019) or graphene oxide-PLGA/HAp (Vazquez-Silva et al., 2022; Fu et al., 2017).

### 2.6 Advantages and limitations

Synthetic polymers, natural polymers, metals, ceramics, and composites exhibit distinct characteristics that make them suitable for different applications. The selection of an ideal material for biomedical implants is dependent upon an array of specific properties, including biocompatibility, mechanical strength, biodegradability, and functionality. Metals, such as titanium and cobalt-chromium alloys, are preferred for structural implants due to their high strength and durability, although they may impose challenges like corrosion and biocompatibility issues in physiological environments (Ali et al., 2020), such as allergic reactions and intoxication (SS, titanium alloys), interfacial loosening or fast degradation (Mg alloys) (Filip et al., 2022).

Synthetic polymers, such as PLA and PCL, stand out for their biodegradability and flexibility but are limited in mechanical strength (Samir et al., 2022), while some polymers may promote osteolysis and produce wearing debris (Filip et al., 2022). However, natural polymers like collagen and chitosan promote excellent biological integration but may be limited by uncontrollable mechanical, degradation rates or lack of thermal stability (Guo et al., 2021; Sergi et al., 2020).

Ceramics, such as hydroxyapatite and zirconia, possess excellent biocompatibility and wear resistance but are brittle, limiting their use to specific applications (Albayrak and Gul, 2024). A study conducted by Neugebauer et al. (2023) found that one-piece ceramic implants achieve osseointegration similar to titanium implants and that fracture risks are low for current commercially available implants. However, ceramic materials also present some disadvantages, such as lack of reliable long-term data, some materials possess high fracture rates (e.g., alumina), and others may be subjected to defects in manufacturing and application on unbalanced lateral loading (Prakash et al., 2021; Thiem et al., 2022a; Andreiotelli et al., 2009).

Composites combine the properties of two or more materials, such as the flexibility of polymers with the rigidity of ceramics, offering versatile solutions for multifunctional applications (Wu et al., 2022; Chen et al., 2022). A comparative overview of these materials is summarized in Table 1, highlighting their advantages, limitations, and common applications.

**TABLE 1:** Comparative overview of common implant materials.

Material	Advantages	Limitations	Common Applications	Ref.
Metals	High mechanical strength, durable	Susceptible to corrosion, potential adverse reactions	Orthopedic implants, dental prostheses	Saha and Roy (2022)
Synthetic polymers	Biodegradable, moldable, cost-effective	Limited mechanical strength	Temporary scaffolds, controlled release	Ershad-Langroudi et al. (2024b)
Natural polymers	Biocompatible, promote cellular regeneration	Low thermal and mechanical stability	Soft tissue grafts, cartilage repair	Asadi et al. (2020)
Ceramics	Biocompatible, wear-resistant	Brittle, low impact resistance	Bone replacement, implant coatings	Zhai et al. (2021), Rafiq et al. (2023)
Composites	Adjustable properties, combination of strength and biocompatibility	Complex fabrication, higher coast	Multifunctional implants, advanced scaffolds	Jindal et al. (2021)

## 3 Attributed therapeutic functions

### 3.1 Metallic implants

#### 3.1.1 Titanium alloys

Titanium alloys are widely utilized in biomedical applications due to their mechanical strength, corrosion resistance, and biocompatibility (Catanio Bortolan et al., 2021; Yang et al., 2020). Among these, Ti6Al4V is one of the most commonly used biomaterials, offering high strength alongside lower stiffness and density, making it superior to many other metals used in implants. However, the accumulation of titanium, vanadium, and aluminum in the tissues surrounding implants with high wear rates, such as artificial joints, has been observed, as noted by Tüten et al. (2019).

In a related development, Ramskogler et al. (2016a) successfully developed biocompatible ceramic-biopolymer coatings on electron-beam-structured titanium alloy surfaces. They deposited homogeneous single layers of chitosan (CHI) composite coatings containing titania nanoparticles (n-TiO_2_) using electrophoretic deposition onto the three-dimensional (3D)-structured surface of the Ti6Al4V alloy. This technique favored an increase in surface area due to the electron beam structuring process. Moreover, it proved versatile in creating uniform chitosan coatings enriched with active ceramic compounds on structured surfaces. This method allows for the incorporation of various types of nanoparticles into chitosan composite coatings, enabling the modification of the surface roughness of the implants.

Păun et al. (2023) developed a new hybrid coating of silk fibroin (SF) with ZnO, an antimicrobial agent, for Ti dental implants using TiO_2_ nanostructures. The SF was deposited on the surface of the TiO_2_ nanotubes using the electrospinning technique, and the ZnO nanoparticles were incorporated using three different methods. The results showed that all the samples had improved roughness and hydrophilicity, as well as corrosion stability. In addition, the antibacterial tests showed that the hybrid coating had good antibacterial activity, inhibiting proliferation by approximately 54% for *S. aureus* and *E. faecalis* bacteria.

Similarly, Elyada et al. (2014) coated sandblasted and acid-etched titanium plates with multilayers produced from various biomacromolecule blends. These organic coatings were created using Layer-by-Layer (LbL) deposition of poly-L-lysine alternated with poly-L-glutamate, poly-L-aspartate, or chondroitin sulfate (CS). The composition and molecular structure of the terminal layer significantly influenced calcium phosphate nucleation, whereas the LbL technique facilitated the bioactive incorporation of growth factors (GFs) or drugs, enhancing the bioactivity of artificial implants for bone and tooth regeneration.

Furthermore, Yu et al. (2020) developed a titanium implant with antibacterial properties and a potential for osteo/angiogenic differentiation. This was accomplished through the utilization of hyaluronic acid-gentamicin conjugates (HA-Gm) and chitosan multilayers (CHI) on deferoxamine substrates loaded with titania nanotubes using the layer-by-layer (LbL) assembly technique. Their research established a multifaceted drug-device combination strategy, showcasing both antibacterial properties and the promotion of pro-osteo/angiogenic differentiation. The on-demand release of deferoxamine, triggered by exogenous hyaluronidase, exhibited enzymatic and bacterial responsiveness. Additionally, the nanotubes/deferoxamine/HA-Gm system demonstrated effective antibacterial activity against *E. coli* and *S. aureus*, while also enhancing the adhesion and differentiation of mesenchymal stem cells (MSCs), underscoring its potential applications in orthopedics.

#### 3.1.2 Stainless steel alloys

Stainless steel is an iron-based alloy that contains a minimum of 10.5% chromium by weight, along with varying amounts of nickel. It can be categorized based on its composition into chromium and chromium-nickel types, or by its microstructure into four families: martensitic, ferritic, austenitic, and duplex (Taxell and Huuskonen, 2022). Each type of stainless steel has specific applications in medical devices. For example, martensitic stainless steels are commonly used for surgical instruments, such as scissors and scalpels, due to their hardness and durability (Mainier, 2013). Ferritic steels, offering good corrosion resistance, are commonly found in hospital equipment, including solid instrument handles, pins, and fasteners (Bandyopadhyay et al., 2023). In contrast, austenitic steels, known for their excellent corrosion resistance and biocompatibility, are frequently utilized in implants and surgical tools, primarily because of their cost-effectiveness (Manam et al., 2017a). Lastly, duplex stainless steels, which combine the features of both austenitic and ferritic steels, are ideal for high-strength, corrosion-resistant applications, such as mini-screw implants in orthodontic dentistry (Du et al., 2016). Additionally, fine-tuning between the ε-martensite and γ-austenite phases in some iron-based alloys can promote a shape memory effect, which is explored in novel devices (Ferretto et al., 2021).

The design of biomedical implants involves careful consideration of both the bulk and surface properties of biomaterials (Davis et al., 2022). Several studies have investigated the design and production of SS implants, including the work of Chacón et al. (2022). They developed high-precision, low-cost implants using a fused filament fabrication 3D printer with a stainless steel/polymer composite filament. This innovative approach allows for creating customized veterinary implants that better fit fractured bones, thereby improving patient comfort and reducing the risk of further injury.

Moreover, some studies have aimed to impart antimicrobial properties to stainless steel alloys. For example, Jensen et al. (2022) produced an orthopedic antibacterial coating designed to release antibiotics shortly after insertion, aiming to prevent perioperative contamination. The coating was evaluated in infected pigs and applied to stainless steel implants featuring a porous layer of titanium microspheres that increased the surface area and anchoring sites available for the gentamicin-silica/polyethyleneimine (PEI) xerogel application. The rapid release of gentamicin demonstrates its potential to protect surgical procedures against *Staphylococcus aureus* and highlights its applicability in cementless arthroplasty or osteosynthesis. However, while antibacterial efficacy has been established, it remains limited to a single bacterial strain.

Lastly, Wang et al. (2020) developed a new coating approach for stents using 316 L stainless steel. They applied mussel adhesive protein (MAP) to immobilize biomacromolecules and create bioactive films. Vascular endothelial growth factor (VEGF) and cluster of differentiation 34 (CD34) antibodies were immobilized on the MAP-coated surface. The VEGF-functionalized substrates were tested in endothelial cell cultures, while the ability of the coated stents to capture CD34^+^ cells was verified *in vitro*. This simple immersion technique in MAP solution presents a promising strategy for controlling cellular behavior in vascular implant materials.

#### 3.1.3 Cobalt-chromium alloys

Cobalt (Co) is widely utilized in biocompatible alloys that exhibit resistance to corrosion and wear. These alloys often combine cobalt with chromium (Cr) and other elements—such as manganese (Mn), molybdenum (Mo), or nickel (Ni) — to create high-performance materials (Kovochich et al., 2021; Grosgogeat et al., 2022). In dentistry, cobalt-chromium (Co-Cr) alloys have been used for years in removable dental prosthetics and orthodontic devices, like brackets and arch wires, due to their strength, durability, and low cost (Grosgogeat et al., 2022). For instance, the L605 alloy (Co-20Cr-15W-10Ni), commonly found in cardiovascular stents, is non-magnetic and possesses good radiopacity, high strength, and ductility. However, it contains 10% nickel, which can cause allergic reactions, is potentially carcinogenic, and may promote vessel restenosis (Catanio Bortolan et al., 2020). Both Co and Cr exhibit some toxicity, and forming a corrosion-resistant layer can help mitigate metal ion release. Although allergic reactions are rare, the release of ions can lead to adverse effects on the body (Fratila et al., 2023).

Several studies have explored surface modifications to enhance the biocompatibility of Co-Cr alloys. Diaz-Rodriguez et al. (2021a) produced cobalt-chromium discs and stents, coating them with a CD31-mimetic peptide, polyethylene glycol (PEG), and polydopamine. They found that the coatings reduced the formation, activation, and aggregation of leukocytes and platelets, improved endothelialization of the metal surface, and maintained a low release of soluble inflammatory and thrombotic biomarkers compared to bare metal devices. Notably, only the CD31 coatings achieved a combination of these beneficial effects, while PEG and polydopamine also contributed to some degree of biocompatibility.

Johnbosco et al. (2021) further enhanced the hemocompatibility of cobalt-chromium (Co-Cr) vascular stents by developing surface coatings using a four-arm cross-linked poly (ethylene glycol) and heparin biohybrid hydrogel. They created a hydrophilic bonding layer with dual copolymers of silane and poly(ethylene alt-maleic anhydride), ensuring uniform distribution of the coating over the thin stent struts and providing mechanical stability during deployment. The homogeneity and stability of the coating during stent expansion were confirmed, demonstrating a significant reduction in the procoagulant and inflammatory activity compared to bare metal.

In another study, Liu et al. (2015) evaluated the bioactivity of a Co-Cr alloy by immobilizing cross-linked poly-γ-glutamic acid on its surface through self-assembled monolayers of 11-aminoundecylphosphonic acid. The results indicated that the surface became highly hydrophobic following poly-γ-glutamic acid immobilization. Subsequent treatment with CaCl_2_ and immersion in simulated body fluid resulted in the formation of poorly crystalline apatite, demonstrating that chemical modification can effectively enhance the bioactivity of bioinert materials.

#### 3.1.4 Magnesium alloys

Magnesium alloys are lightweight materials with high specific resistance and an elastic modulus similar to natural bone, contributing to their excellent biocompatibility. As the second most abundant intracellular cation in the human body (Hernández-Montes et al., 2023), magnesium (Mg) holds significant potential for use in biodegradable and non-toxic temporary orthopedic and vascular implants (Verma et al., 2024). These alloys are bioactive and biotolerant, providing essential support for tissue regeneration and fully degrading in biological environments, making them promising alternatives to permanent implants (Rahman et al., 2020). For magnesium implants to be considered biocompatible, they must demonstrate high resistance to wear and corrosion while degrading at a rate compatible with bone regeneration. The release of Mg^2^⁺ ions is crucial for activating enzymatic processes and promoting cell growth. However, rapid degradation can compromise their mechanical properties, limiting broader clinical applications in orthopedic implants and cardiovascular stents (Zhao et al., 2023).

In this context, Khatun et al. (2024) investigated the high corrosion rates of magnesium and WE54 Mg alloys, which restrict their use in biomedical applications. Their study focused on developing hybrid coatings combining organic polylactic acid (PLA) and inorganic hydroxyapatite (HAp). These hybrid coatings significantly enhanced corrosion protection and biocompatibility, effectively reducing degradation and *in vitro* corrosion rates in body fluid solutions. Notably, the 1% HAp/PLA hybrid coating on WE54 Mg alloy surfaces demonstrated considerable potential for implant applications, showcasing improved properties across multiple performance indicators.

Zhou et al. (2021) also addressed challenges in magnesium alloys, such as limited manipulation, antibacterial properties, and brittle osteoinductivity, by applying an antimicrobial peptide (AP)-loaded SF composite coating on MgO-coated AZ31 magnesium alloy. The MgO layer, formed using anodizing and electrodeposition methods, enhanced corrosion resistance and overall functionality. The resulting composite coatings provided a smooth, hydrophilic surface and significantly improved corrosion resistance. Notably, *E. coli* colonization was reduced on the MgO-AP coatings, likely due to the synergistic effects of the APs and Mg^2^⁺ ions. Overall, the findings indicate that combining Mg^2^⁺ ions and APs with SF enhances the magnesium alloy surface’s ability to inhibit bacterial adhesion and promote bone regeneration.

Additionally, Hernández-Montes et al. (2023) reviewed the applications of cerium (Ce) coatings in Mg alloys, highlighting their redox properties that grant self-healing capabilities, besides their anti-inflammatory, antioxidant, and osteoconductive properties. Kim et al. (2020) further incorporated Ce during calcium electrodeposition on magnesium implants, resulting in a thicker and denser coating. Over time, Ce(OH)₃ or CeO₂ rapidly formed in areas of film damage. The film underwent hydrothermal treatment with hyaluronic acid (HA) and carboxymethylcellulose, facilitating the diffusion of Ce ions into the coating. This process maximized the self-healing capabilities of the magnesium substrate and oxide film, ultimately improving the initial corrosion resistance of magnesium implants while protecting against corrosion resulting from local damage and biodegradation.

#### 3.1.5 Degradation and ions’ role

Ion release in metallic implants typically occurs due to the electrochemical interaction between the implant material and its surrounding biological environment. This process is influenced by factors such as the implant material’s composition, the local pH, and the presence of biological fluids, and it can be promoted by corrosion or mechanical wear mechanisms. The release of metal ions promotes tissue reactions, in which some ions can positively influence material-tissue interactions, aiding bone regeneration and wound healing (Vasconcelos et al., 2016). In contrast, others can cause adverse tissue reactions, such as inflammation, hypersensitivity, or even toxicity.

Ions like magnesium (Mg^2^⁺), zinc (Zn^2^⁺), iron (Fe^2^⁺), vanadium (V³⁺), and aluminum (Al³⁺) when released in a controlled manner (e.g., in bioresorbable devices) can promote osteoinduction, enzyme activation, and bacterial inhibition (Awais et al., 2022; Glenske et al., 2018). Mg^2^⁺, for example, stimulates cell growth and bone regeneration, especially in biodegradable alloys, which gradually degrade as bone heals (Uppal et al., 2022).

However, excessive ion release can lead to toxicity, such as aluminum or vanadium accumulation in tissues, posing long-term risks (Costa et al., 2019; Zaffe et al., 2004). Additionally, degradation of metallic components may compromise implant stability. In stainless steel and cobalt-chromium alloys, nickel (Ni) and chromium (Cr) can cause allergic reactions and corrosion, releasing toxic ions (Manam et al., 2017b). Controlling ion release and preventing corrosion are vital for improving implant biocompatibility and longevity. Advances in biomimetic coatings and surface modification techniques like layer-by-layer (LbL) deposition show promise in enhancing implant safety and therapeutic efficacy (Liu et al., 2024; Liu et al., 2010).

Table 2 synthesizes the therapeutic functions of biomacromolecular systems integrated into metallic implants.

**TABLE 2:** Biomedical applications of metallic implants integrated with biomacromolecular systems.

Alloys	Biomacromolecules	Application	Therapeutic effect	References
Titanium	Type I collagen, RGD peptide, and type I collagen/chondroitin sulfate	Orthopedic implants	Early bone remodeling	Rammelt et al. (2006)
Titanium	Glycine–phenylalanine–hydroxyproline–glycine–glutamate–arginine	Orthopedic implants	Enhances bone repair and osseointegration	Reyes et al. (2007)
Titanium	Silk fibroin	Implants in diabetics	Biocompatibility and optimal anti-diabetic effects	Ma et al. (2021)
Titanium	Rhamnolipid	Orthodontic implants	Reduction of *Staphylococcus* spp. biofilm formation	Tambone et al. (2021)
Titanium	N-halamine polymeric	Orthodontic implants	Prevention and treatment of peri-implant infection	Wu et al. (2021)
Stainless steel	Cinnamon oil and chitosan	Orthopedic implants	Protection against the formation of microorganism biofilm	Magetsari et al. (2014)
Stainless steel	Amoxicillin-doped hyaluronic acid/fucoidan	Orthopedic implants	Biocompatibility, improvement of osseointegration, and antimicrobial effects	Tack et al. (2015)
Stainless steel	Sulfonated chitosan and dopamine	Metallic implants in contact with blood	Calcification resistance and antithrombogenic properties	Campelo et al. (2017)
Stainless steel	Cellulose acetate fibers loaded with daptomycin	Orthopedic implants	Protective coating and drug delivery vehicle	Faria et al. (2022)
Stainless steel	Chitosan and gelatin	Permanent implants	Improved surface characteristics and enhanced corrosion resistance	Improvement of corrosion resistance of (2023)
Cobalt-chromium	Elastin-like recombinamers genetically modified	Cardiovascular stents	Promotes endothelial cell adhesion and spreading	Castellanos et al. (2015)
Cobalt-chromium	Hyaluronic acid	Orthopedic implants	Interferes with corrosion resistance	Radice et al. (2019)
Cobalt-chromium	Bone morphogenetic protein-7	Orthopedic implants	Reduces fibrosis on tissue-implant interface	Tan et al. (2013)
Cobalt-chromium	CD31-mimetic coating	Coronary stent	Vascular homeostasis and arterial wall healing	Diaz-Rodriguez et al. (2021b)
Nitinol	Chitosan–tungsten composite	Orthopedic implants	Increases corrosion resistance	Oliveira et al. (2023)
Tantalum	Polyhydroxyalkanoates	Orthopedic implants	Shows antibacterial properties	Rodríguez-Contreras et al. (2019)
Magnesium	Heparin and carboxymethyl chitosan	Vascular implants	Exhibites antithrombotic and antibacterial activities	Pei et al. (2019)
Magnesium	Simvastatin-loaded gelatin nanosphere/chitosan	Orthopedic implants	Promotes osteogenic differentiation and vascularization	Qi et al. (2021)
Magnesium	Silk fibroin and cellulose nanocrystal	Orthopedic implants	Enhances corrosion resistance and improves cytocompatibility	Asadi and Ramasamy (2022)
Magnesium	Chitosan	Biological implants	Protects mg from corrosion and tribocorrosion	Xu et al. (2022)
Magnesium	Tannic acid and hyaluronic acid	Orthopedic implants	Reduces the corrosion rate	Salsabila et al. (2023)
Zinc-magnesium	Biomimetic zwitterionic phosphorylcholine chitosan coating	Biological implants	Increases the resistance against corrosion attack	Sheng et al. (2020)
Iron‐manganese	Collagen	Biological implants	Increases the initial cell viability and adhesion	Huang et al. (2020)

### 3.2 Synthetic-polymer implants

#### 3.2.1 Ophthalmic implants

Ophthalmic implants provide tools for the management of a diverse range of ocular conditions, with synthetic polymers being often used due to their biocompatibility, flexibility, and favorable optical properties (Ferraz, 2022). Intraocular lenses (IOLs) exemplify this application, serving as substitutes for the natural lens, particularly in individuals with cataracts (Traian and Damien, 2016). Poly (methyl methacrylate) (PMMA), silicone, PCL, and PLA are commonly employed in the fabrication of these lenses owing to their transparency and capacity to maintain a consistent form within the eye. IOLs allow patients to restore vision without reliance on corrective eyewear such as glasses or contact lenses.

PMMA was one of the first materials used in IOLs due to its excellent clear optics and biocompatibility, as well as its durability and ability to maintain structural integrity in the eye, allowing for stable and clear visual correction (Karayilan et al., 2021). However, Karaaslan Tunç et al. (2024) emphasize that the rigidity of PMMA IOLs requires larger incisions during surgery, resulting in prolonged recovery periods.

To overcome this limitation, silicone was introduced as a more flexible alternative. Silicone lenses can be folded and inserted through smaller incisions, leading to less invasive procedures and quicker recovery (Sheardown et al., 2020). Studies have demonstrated that silicone IOLs exhibit excellent elasticity and resistance to deformation, ensuring they adapt well to the ocular environment while maintaining high optical transparency with a refractive index range from 1.41 to 1.46 (Lloyd et al., 2001; Čanović et al., 2019).

**FIGURE 2:**
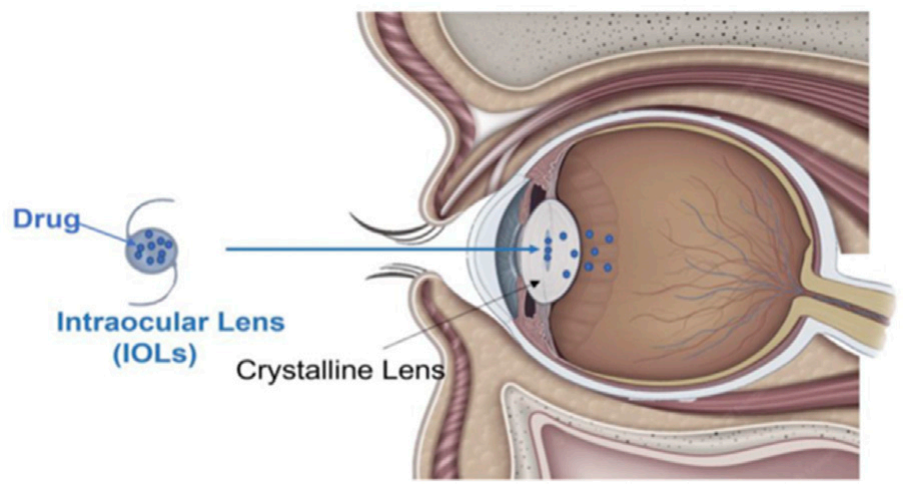
Schematic representation of drug-loaded IOLs: sustained intraocular release via diffusion following cataract surgery. Reprinted from Pelusi et al. (2023)/CC BY 4.0.

Furthermore, advances in silicone IOL surface modification enable the development of implants with additional therapeutic capabilities. Modifying these surfaces aiming to incorporate controlled drug delivery systems (Figure 2), may be especially useful in short- and long-term treatment after surgeries (Wu et al., 2023). These modified silicone IOLs can gradually release therapeutic agents, such as anti-inflammatories or antibiotics, directly into the eye, helping to prevent postoperative complications such as infection or inflammation (Topete et al., 2021). Besides that, the incorporation of medications directly into the IOL eliminates the need for additional topical treatments. This can significantly improve patient recovery, making silicone IOLs a platform not only for vision correction but also for ongoing therapeutic care postoperatively.

According to Ferraz (2022), hydrophobic or hydrophilic hydrogels and acrylics can be combined with silicone to create more flexible lenses. They can be bent at room temperature and, when inserted into the eye, return to their original shape and size. When compared to silicone IOLs, their implantation is slower and provides greater control during the procedure.

PCL and PLA are biodegradable polymers that are being researched for use in temporary lens implants or drug-releasing IOLs in the field of ophthalmology. These materials degrade over time, potentially reducing the need for additional surgeries (García-Estrada et al., 2021; Sakpal et al., 2022). PCL offers slow degradation and is suitable for long-term applications where gradual bio-resorption is required (Tajvar et al., 2023), while PLA provides a controlled degradation rate and is being investigated for its ability to release therapeutic agents after surgery to prevent infection or inflammation (Liu et al., 2020a; Çağlar et al., 2024; DeStefano et al., 2020).

Boia et al. (2019) developed porous PCL IOLs using supercritical carbon dioxide (scCO_2_), which exhibits faster degradation than conventional implants. Their greater porosity also allows greater dexamethasone release rates. Furthermore, they did not cause toxicity to retinal cells and, in *in vivo* tests, preserved the function and structure of retinas in Wistar rats. The results suggest that these PCL implants can be inserted into the vitreous cavity through a minimally invasive procedure, highlighting their potential for prolonged drug delivery in ocular diseases.

Recent advancements in additive manufacturing have introduced the possibility of fabricating 3D-printed IOLs, offering even greater customization and precision in ophthalmic implants. 3D printing allows for the creation of IOLs with complex designs and personalized geometries tailored to individual patients, addressing specific visual impairments more effectively. Shiblee et al. (2018) used a custom optical 3D printer to create strong and thermoresponsive shape memory gels of various formats and sizes. By adjusting the monomer composition, they were able to control the elasticity, thermal resistance, and transparency of the shape memory gels. These gels demonstrated excellent fixation, recovery, and deformation properties, making them suitable for use in biomedicine, robotics, and sensors. One notable application is their potential use as IOLs due to their high transparency, ability to adjust the refractive index, and shape memory properties, allowing for compressed insertion and expansion after implantation. These customizable features suggest that these gels could enhance comfort and visual performance, offering a viable alternative for developing IOLs for ophthalmic surgeries.

In summary, the progression of ophthalmic implants, particularly intraocular lenses, illustrates the significant impact of synthetic polymers in advancing ocular healthcare. The continuous developments in materials such as PMMA, silicone, PCL, and PLA, coupled with innovative methods like 3D printing and surface modifications for precise drug delivery, present new opportunities for tailored treatment approaches. As these technologies continue to evolve, they hold the potential not only to enhance vision correction but also to mitigate postoperative complications and optimize patient outcomes.

#### 3.2.2 Cardiac prostheses and vascular stents

Cardiovascular diseases figure as a significant global health concern, leading to high morbidity and mortality rates (Gaidai et al., 2023). Various implants, including heart valve prostheses, vascular stents, vascular grafts, cardiac adhesives, and pacemakers, play a major role in restoring normal cardiovascular function and improving patient quality of life. These materials must possess basic properties for a successful application in cardiovascular devices, including biocompatibility to avoid adverse reactions, hemocompatibility, durability to withstand mechanical stress and resistance to fatigue and degradation (Ershad-Langroudi et al., 2024a; Ammann et al., 2021). Elasticity and flexibility are essential for stents and grafts, enabling them to adapt to the dynamic nature of blood vessels (Seidman et al., 2020; Gupta and Mandal, 2021). Additionally, cardiac adhesives must ensure effective tissue bonding while promoting healing, requiring optimal adhesive strength and performance in wet environments (Nam and Mooney, 2021). Synthetic polymer-based implants have gained prominence due to their biocompatibility, flexibility, and customizable properties. Materials such as PLA, PCL, PE, PLGA, PEG, and PLLA are widely used in the fabrication of vascular stents and grafts, offering durability and the potential for drug delivery to prevent complications like restenosis. As illustrated in Table 3, different synthetic polymers have been applied in cardiovascular devices using various manufacturing methods such as electrospinning, melt mixing, and casting. These materials offer specific therapeutic advantages such as enhancing endotheliogenesis, preventing thrombosis, and reducing post-surgical complications such as inflammation and adhesions.

**TABLE 3:** Applications of different polymers in cardiovascular devices and their therapeutic effects.

Polymer	Application	Manufacturing methods	Therapeutic effect	Reference
PLLA/PDA^a^	Cardiovascular stents	Solvent casting/coating	Increasing endothelialization; anti-inflammatory properties	Zhang et al. (2023)
PPC^b^/PCL	Self-expandable stents	Melt-blending	Blood pressure reduction; angina treatment; stroke prevention; control of arrhythmias; treatment of heart failure	Zheng et al. (2017)
PCL	Vascular grafts for bypass surgeries	Dip-spinning/solution blow spinning	Minimizing complications such as thrombosis and intimal hyperplasia	Akentjew et al. (2019)
PU/PCL	Cardiovascular stents	Melt blending	Artery dilation and reduced abnormal stress in vascular tissues	Ajili et al. (2009)
PEG/PLCL^c^	Bioabsorbable cardiovascular stents	Copolymerization/functionalization	Increasing endothelialization; reduction of the risk of thrombosis	Pacharra et al. (2020)
PEG	Anti-adhesion membrane in cardiac surgery	Functionalization	Prevention of postoperative adhesions; inhibits the initial formation of fibrin networks and reduces inflammation	Hashimoto et al. (2022)
PLA/PGS^d^	Cardiac tissue	Electrospinning	Tissue regeneration; neovascularization	Flaig et al. (2020)
PANI^e^	Cardiac Biomarkers	Electrochemical deposition	Diagnostic tools	Lee et al. (2012)
pHMGCL/PCL	Cardiac tissue	Electrospinning	Better biocompatibility; retention and guiding the growth of cardiac cells	Castilho et al. (2017)
PLLA/PCL/PA-6	Cardiac implants	Electrospinning	Prevention of complications such as late stent thrombosis	Matschegewski et al. (2022)
PU/PDMS/PTMO	Adjustable cardiovascular devices	Chain extension	Hemocompatibility, antithrombogenicity reduced inflammation; antimicrobial activity	Silvestri et al. (2011)

^a^ Polydopamine.

^b^ Poly(propylene carbonate).

^c^ Poly-L-lactide-co-ε-caprolactone.

^d^ Poly(glycerol sebacate).

^e^ Polyaniline.

#### 3.2.3 Orthopedic implants

Orthopedic implants are medical devices designed to replace, support, or enhance the function of bones, joints, and other skeletal structures in individuals with orthopedic conditions, such as fractures, arthritis, or deformities (Shen and Shukla, 2020). These implants are directly responsible for restoring mobility, alleviating pain, and improving the overall quality of life for patients. Common types of orthopedic implants include joint prostheses (e.g., hip and knee replacements), bone plates, screws, and spinal devices. They are often required when natural healing is insufficient or when the structure of the bone or joint has been severely compromised due to injury or disease (Hallab et al., 2020; Meng et al., 2023; Tapscott and Wottowa, 2023).

For orthopedic implants to be successful, they must possess several key properties. Biocompatibility is essential to prevent immune reactions and ensure the long-term stability of the implant within the body (Liu et al., 2020c). Mechanical strength and durability are also critical, as the implants need to withstand the substantial forces and stresses exerted on bones and joints during daily activities (Huo et al., 2022). Additionally, materials used for orthopedic implants must exhibit good wear resistance and corrosion resistance to maintain their integrity over time (Ramya, 2024).

Ferroni et al. (2022) reported a 3D-printed PEEK scaffold coated with methacrylated hyaluronic acid - hydroxyapatite hydrogel by a dip-cure process. Hyaluronic acid is by itself a therapeutic agent, being able to promote cell adhesion, proliferation, migration, angiogenesis, and wound healing as a constituent of the extracellular matrix (ECM). However, its modified version also allows the physical embedding of hydroxyapatite, which also presents biological activity. The hyaluronic acid present in the coating promoted MSCs adhesion and proliferation and contributed to osteogenic differentiation, while the hydroxyapatite promoted ECM mineralization (Ferroni et al., 2022).

The study by Feerick et al. (2013) explores the use of fracture fixation devices made of metal materials and CF-PEEK. The focus is on their applications in treating proximal humerus fractures, particularly three-part fractures, which are prevalent among older individuals. CF-PEEK, produced through 3D printing, demonstrates superior stress distribution compared to metal implants due to its elastic modulus being closer to that of cortical bone. This characteristic reduces stress in the screw tip area. Additionally, the incorporation of calcium phosphate cement as a therapeutic agent enhances fixation stability by filling bone voids and minimizing displacement between bone fragments, ultimately leading to improved treatment outcomes.

According to Hallab et al. (2020), highly crosslinked ultra-high molecular weight polyethylene (UHMWPE) is extensively utilized in arthroplasties because of its capacity to endure loads with minimal friction. Its greater crystallinity, compared to other types of PE, provides better mechanical performance, although it somewhat compromises its ductility and fracture toughness. However, due to its limitations, such as lower wear resistance, tendency to oxidative degradation, and lower impact strength, the use of UHMWPE in some biomedical applications is restricted. These challenges can be overcome by reinforcing the matrix with hydroxyapatite (HAp), which improves its mechanical properties, such as stiffness and wear resistance, as well as promoting osseointegration, making it more suitable for long-term orthopedic implants.

Kang et al. (2016) investigated composites of UHMWPE reinforced with HAp in different proportions of micro and nano-HAp, aiming to enhance their mechanical and tribological properties for use in joint implants, such as hip and knee prostheses. The results show that the addition of HAp increases the compressive strength, hardness, crystallinity, and melting temperature of UHMWPE, with nano-HAp-containing composites exhibiting better mechanical performance compared to those with micro-HAp. The study also identified improvements in wettability and wear resistance, especially in composites with 10% nano-HAp, which displayed a low coefficient of friction. However, when the HAp content exceeds certain limits, changes occur in the wear mechanisms, such as surface rupture and delamination.

Furthermore, both PEEK and UHMWPE are non-degradable polymers, which may limit their use in some orthopedic applications where resorption of the material may be desirable. Alternatives such as PLA have gained prominence in the development of orthopedic implants due to their biodegradability. PLA promotes integration with bone tissue during its degradation, which reduces the need for a second surgery to remove the implant (Feng et al., 2021). This property makes PLA a promising option for applications focused on bone tissue restoration, offering a sustainable solution that better adapts to patients’ needs (Chen et al., 2020). A recent study by Heidari et al. (2017) investigated the production of biodegradable polylactic acid (PLA) bone screws by injection molding, evaluating three formulations: PLA0 (neat), PLA3 (5% PLA nanoparticles with Triclosan), and PLA9 (5% Triclosan and 5% nanohydroxyapatite). Characterization included thermal, mechanical, and rheological properties, with computational models for process optimization. Triclosan provided antibacterial properties, while nano-hydroxyapatite (nHAp) improved bioactivity. The PLA9 screw showed the highest strength to force (3484 N) and is seen as promising for biomedical applications, although further research is needed to evaluate its *in vivo* performance and biocompatibility.

#### 3.2.4 Controlled drug release devices

Controlled drug-release devices based on synthetic polymers have been widely explored due to their ability to modulate drug release precisely and efficiently (Sung and Kim, 2020). These systems are designed to maintain adequate therapeutic concentrations for prolonged periods, avoiding unwanted peaks and fluctuations, as well as reducing the adverse effects common in conventional drug administration (Adepu and Ramakrishna, 2021). Synthetic polymers may be chosen in this context due to their ability to form stable, tunable, and highly versatile matrices.

These synthetic polymers can be applied in several medical areas, such as bone implants for tissue regeneration, antibiotic delivery in chronic infections, and subcutaneous devices for hormone release (Jagur-Grodzinski, 2006). Do et al. (2015) created an implantable material able to combat periodontitis, using a combination of different types of PLGA as drug release rate controlling polymers, hydroxypropyl methylcellulose as adhesive polymer, and doxycycline or metronidazole as drugs. The components were mixed in an appropriate solvent and cast in agarose gel molds for solvent extraction, creating effective locally controlled drug delivery systems.

Eğri and Eczacıoğlu (2017) developed PLA-PEG-PLA scaffolds for bone tissue engineering applications, emphasizing the sequential release of two growth factors: VEGF and bone morphogenic protein 2 (BMP-2). The PLA-PEG-PLA copolymer, which has biocompatible and biodegradable properties, was used in the fabrication of the scaffolds through the freeze-drying/cryogelation technique combined with salt leaching, resulting in a porous structure ideal for three-dimensional cell growth. VEGF, which promotes angiogenesis, showed rapid release, while BMP-2, responsible for stimulating osteogenesis, was released in a controlled manner for up to 100 days. *In vitro* tests demonstrated that the scaffolds presented high biocompatibility without significant cytotoxic effects, in addition to favoring the adhesion and proliferation of osteoblastic cells.

Drug delivery implants in oncology have shown great potential for localized treatments, such as in brain and breast tumors, where the controlled release of chemotherapeutic agents directly into the tumor tissue increases therapeutic efficacy and minimizes adverse effects.

Kaetsu et al. (1987) synthesized biodegradable polymers that were molded together with drugs for cancer therapy into needles and buttons that can be implanted under the skin or directly into tumors. For instance, poly (D,L-lactic acid) was produced by radiation polymerization, and a Luteinizing hormone-releasing hormone agonist was used as the active compound, both disappearing after the release process.

The study by Hao et al. (2021) describes the development of a drug-loaded implantable prosthesis using PDMS as a matrix. They incorporated Poly(lactic-co-glycolic acid) (PLGA) microparticles into PDMS for the controlled release of the chemotherapeutics paclitaxeland doxorubicin (DOX). The prosthesis was fabricated using 3D printing, allowing for customization of the patient’s anatomy. *In vitro* results demonstrated continuous release of paclitaxel and DOX for over 3 weeks, inhibiting tumor cell growth. The combination of paclitaxel and DOX in microparticles exhibited a synergistic effect, increasing cytotoxicity by 30% in breast cancer cells compared to the individual drugs. Additionally, the prosthesis displayed good biocompatibility and reduced tumor recurrence and metastasis by 60% in mice, leading to a 40% increase in the survival rate compared to control groups. The authors suggest that this approach holds promise as an alternative for breast reconstruction and the treatment of breast cancer following conservative surgery.

Recently, stimuli-sensitive controlled release systems have received considerable attention. These devices can be designed to respond to changes in the physiological environment, such as pH (Bazban-Shotorbani et al., 2017), UV radiation (Fan et al., 2016), electric (Seyfoddin et al., 2015), reactive oxygen species (Wang et al., 2018) or the presence of specific enzymes (Zhang et al., 2017), and release the drug in a controlled manner only in response to these stimuli (Wells et al., 2019). These advances, particularly with stimulus-sensitive synthetic polymers, promise to increase the precision of treatments, making therapies more personalized and effective.

Table 4 exemplifies the smart polymers used in stimuli-responsive drug delivery systems, detailing their applications, therapeutic agents, and outcomes. It summarizes how these materials have the potential to improve specific treatments, such as cancer therapy and intraocular drug delivery.

**TABLE 4:** Stimuli-sensitive controlled drug release systems from synthetic polymers, their therapeutic applications, and results achieved.

Stimuli type	Polymer	Application	Therapeutic agent	Result	Reference
pH	PLA-PEI^a^	Fluorescent nanoparticles for intracellular imaging and drug delivery	Doxorubicin	Superior fluorescent properties; efficacy in suppressing MCF-7 cell proliferation	Li et al. (2014)
	PAAm^b^/PMMA-MAA^c^	Intra-articular administration of drugs	Fluorescein	Hydrogel’s elastic modulus control; drug release at low volume; low cytotoxicity to human chondrocyte cells	Pafiti et al. (2016)
Light	mPEG^d^-PLGA^e^	Cancer treatment	BNN6/Doxorubicin	Greater effectiveness of DOX; Significantly greater cytotoxicity against resistant tumor cells	Fan et al. (2016)
pH and Light	CD^f^-MA^g^/NIPAM^h^	Cancer treatment	Methotrexate	High drug loading capacity and controlled release; biocompatibility and hemocompatibility; *In vivo* antitumor efficacy	Das et al. (2019)
Electric	PPy^i^/PMMA	Intraocular administration of corticosteroids	Dexamethasone	Adjustable DexP release rate; better therapeutic efficacy	Seyfoddin et al. (2015)
Reactive oxygen species	PVA^j^	Immunotherapy	Gemcitabine and aPDL1	Tumor regression; increased nontumor T cell infiltration; inhibiting distant tumor growth; formation of memory T cells	Wang et al. (2018)
Specific enzymes	PEG	Cancer treatment	Gemcitabine	Improved antitumor efficacy *in vivo; low toxicity in normal tissues*	Zhang et al. (2017)

^a^ Polyethyleneimine.

^b^ Poly(acrylamide).

^c^ Methyl methacrylate-methacrylic acid copolymer.

^d^ Polyethylene glycol monomethoxycaprolactone.

^e^ Poly(lactic acid-co-glycolic acid).

^f^ Cyclodextrin.

^g^ Methacrylic acid.

^h^ N-isopropylacrylamide.

^i^ Polypyrrole.

^j^ Poly(Vinyl Alcohol).

### 3.3 Biopolymeric implants

#### 3.3.1 Cellulose-based devices

Cellulose is a polysaccharide with the molecular formula (C_6_H_10_O_5_), consisting of a linear chain of D-glucose units connected by β(1→4) bonds. It is a natural polymer found as the main constituent of plants and natural fibers, and some bacteria, such as *Komagataeibacter xylinus*, can also synthesize cellulose (Maia et al., 2021; Eslahi et al., 2020). In both its pure and chemically modified forms, cellulose exhibits specific mechanical and biological properties, including biocompatibility, biodegradability, and low toxicity (Biswas et al., 2022). However, to enhance its properties, cellulose-based composites have been developed, benefiting from the synergistic effects of their components. These composites can also incorporate therapeutic agents, expanding their applications in fields such as osteoinduction, osteoconduction, and anti-inflammatory treatments (Janmohammadi et al., 2023).

Tabary et al. (2014) produced a chlorhexidine (CHX)-loaded biodegradable cellulosic device for periodontal pocket treatment. In this study, cellulosic (paper) absorbent points were first oxidized and grafted with β-cyclodextrin or maltodextrin, capable of forming reversible inclusion complexes with different organic compounds. The CHX digluconate antiseptic agent was then incorporated into this system by its interaction with β-cyclodextrin/maltodextrin carboxylic groups. The authors highlighted the release results obtained by the maltodextrin system that sustained antibacterial activity against four periodontal pathogens and the possibility of trying other active agents such as ciprofloxacin.

Various nanocellulose materials can be derived from different sources of cellulose through chemical, physical, and biological methods (Deng et al., 2022). A notable example is the study by Rakib Hasan Khan et al. (2022), which developed cellulose nanofiber scaffolds for prolonged drug release systems using doxorubicin (DOX) as an anticancer agent. The incorporation of additives such as gelatin was also tested to enhance the material’s properties. *In vitro* evaluations using pancreatic cancer cells (MIA PaCa-2 and PANC-1) demonstrated the scaffold’s ability to suppress cancer cell proliferation. In *ex vivo* analyses using a patient-derived xenograft model, the released DOX successfully reduced Ki-67-positive pancreatic cancer cells.

In another study, Tarrahi et al. (2021) developed carboxymethyl-diethylaminoethyl cellulose scaffolds for the sustained delivery of curcumin. This novel cellulose derivative, featuring positively and negatively charged functional groups, enhanced cellulose’s versatility as a drug carrier. Curcumin, a well-known chemotherapeutic agent, demonstrated over 99% antibacterial activity and no toxicity in L929 cells. Similarly, Ashraf et al. (2020) developed multifunctional CNF scaffolds incorporating TiO_2_ via electrospinning and silver nanoparticles (AgNPs) through *in situ* deposition to improve antibacterial properties. These nanofibers exhibited significant antibacterial efficiency against *Escherichia coli* and *Staphylococcus aureus*, and their biocompatibility was confirmed through fibroblast testing, highlighting their potential for use in tissue regeneration.

Bacterial cellulose (BC) has garnered scientific interest in bone regeneration applications due to its three-dimensional nanofiber structure, biocompatibility, biodegradability, high surface area, excellent mechanical properties, and flexibility for surface modifications (Maia et al., 2021; Rastogi and Banerjee, 2020). Several studies have incorporated nanomaterials into BC scaffolds to introduce antibacterial properties. For instance, Heydari et al. (2021) incorporated zinc oxide (ZnO) nanoparticles into BC/polypyrrole composite aerogels, enhancing both the antibacterial properties and strength of the material. Sknepnek et al. (2024) produced BC hydrogels functionalized with hydroxyapatite and titanium dioxide (HAp/TiO_2_), demonstrating superior antimicrobial activity when the nanocomposites were incorporated during BC synthesis. Additionally, Yang et al. (2023) developed biomimetic BC hydrogels with silver nanoparticles (AgNPs) and HAp for temporary canthoplasty applications. These hydrogels exhibited antimicrobial properties against *S. aureus* and *E. coli*, attributed to the release of silver ions, which disrupt bacterial membranes.

BC has also been explored as a drug delivery system. Inoue et al. (2020) developed bioabsorbable and bactericidal oxidized BC membranes loaded with CHX for prolonged release in dental applications. These membranes inhibited the growth of *S. aureus*, *E. coli*, and *C. albicans*. Similarly, Stumpf et al. (2020) developed BC membranes biosynthesized with growth factors (GFs), aiming for use as duraplasty membranes in brain applications.

#### 3.3.2 Chitosan-based devices

CHI is also widely used in biomedical applications due to its variety of uses (films, particles, gels, scaffolds, membranes) (Baranwal et al., 2022; López-Valverde et al., 2022b). It is a non-toxic, hydrophilic, biocompatible, biodegradable polymer with good hydration capacity, which means that it can be widely exploited as a biomaterial for tissue engineering. As with cellulose, structural modifications can be made to chitosan’s functional groups, introducing new properties (Kantak and Bharate, 2022; Salave et al., 2022). The properties of chitosan-based implantable devices may also be altered by blending the polysaccharide with other substances (Nair et al., 2015; Lee et al., 2004) or by applying it as composites (Buranapanitkit et al., 2004; Yu et al., 2021; Wang et al., 2021a; Mohan Raj et al., 2018a; Zhang and Zhang, 2002a; Bhowmick et al., 2018a; Barroso et al., 2014; Xu et al., 2019a) during the device production.

Sanpo et al. (2009) developed an antibacterial powder for application as a coating for biomaterials using the cold spray technique. The powders were composed of CHI-Cu (chitosan-copper complex) and CHI-Cu based on Al (aluminum) and were investigated against *E. coli* bacteria, showing that the antibacterial activity increased with increasing CHI-Cu concentration. Shen et al. (2008) also investigated the antibacterial properties through a preliminary study of a chitosan sponge cross-linked with tripolyphosphate containing tetracycline for controlled release in periodontal applications. As in the previous study, chitosan can function as a vehicle for therapeutic agents since this biomaterial showed a controlled release for tetracycline and maintained antimicrobial effects against *S. aureus* and *E. coli* bacteria for up to 11 days.

In addition to its use as an antibacterial agent, chitosan has also shown great potential as a vehicle for the release of growth factors. Soran et al. (2012) developed chitosan scaffolds with alginate microspheres, promoting periodontal tissue engineering, loaded with bone morphogenetic protein-6 (BMP-6) using the electrospray technique. The porous and interconnected structure of the scaffold provided a controlled release of BMP-6, promoting the osteogenic differentiation of mesenchymal stem cells derived from rat bone marrow.

Similarly, Li et al. (2019b) developed a sponge with transforming growth factor-β/chitosan (TGF-β3/CHI) for the repair of periodontal hard and soft tissue defects. The proliferation and osteogenic differentiation behavior of primary human periodontal ligament stem cells were investigated to determine the bioactivity and potential application of TGF-β3 in periodontal disease at different concentrations of TGF-β3/CHIS. Mineralization of the osteogenically differentiated stem cells was confirmed by measuring alkaline phosphatase activity and calcium content, and calcium content in each group increased significantly after 21 and 28 days. Thus, this material showed that TGF-β3/CHI promotes osteogenic differentiation of the stem cells, with potential for application in the repair of incomplete alveolar bone defects.

These studies highlight the versatility of chitosan as a support material for tissue regeneration and its multifunctional properties. While previous studies have focused on drug delivery, these findings on the use of growth factors offer new possibilities for tissue engineering.

The biocompatibility of chitosan was also investigated by Lai (2012) who studied chitosan cross-linked with genipin (GP-CHI) in the anterior chamber of rabbit eye models *in vivo*. Lai observed that GP-CHI implants did not exhibit ocular inflammation, improving the preservation of cell density, anti-inflammatory activities, and biocompatibility with corneal endothelial cells. Thus, it was concluded that choosing cross-linking agents, such as GP, can strongly influence responses to chitosan implants.

#### 3.3.3 Alginate-based devices

Alginate stands out as a versatile polymer due to its biocompatibility, non-toxicity, and structural simplicity. These properties give alginate a wide range of applications in the biomedical field, especially in the regeneration of soft and hard tissues (Lee and Mooney, 2012; Abka-khajouei et al., 2022). Several research groups have been investigating alginate-based hydrogels and scaffolds, in particular controlled release systems for bioactive compounds, approaches to cancer treatment, and advanced strategies for the regeneration of damaged tissues (Tomić et al., 2023).

Yoon et al. (2018) studied a sustained-release system for transforming growth factor-β1 (TGF-β1) using an alginate scaffold and evaluated the effects of its delivery on rotator cuff healing in a rabbit model. Initially, the scaffolds were studied for their proliferation and cellular metabolic activity *in vivo*, as well as their capacity for sustained release of TGF-β1. The scaffold was confirmed to show no cytotoxicity and even increased cell proliferation. Based on its *in vivo* biomechanical and histological properties, after 12 weeks of rotator cuff repair, the authors found better results for sustained release of TGF-β1, concluding that the alginate-bound delivery system for this growth factor may improve rotator cuff healing.

Mata et al. (2017) developed a preliminary study on the *in vivo* articular cartilage regeneration capacity of stem cells cultured in alginate scaffolds. After 3 months of analysis, it was observed that significant cartilage regeneration occurred, especially in animals implanted with hDPSCs, suggesting that alginate scaffolds containing hDPSCs may be useful for articular cartilage regeneration.

Zhao et al. (2022) developed a multifunctional sodium alginate scaffold incorporating modified PLLA microspheres loaded with ibuprofen based on cryogenic 3D printing combined with Sr^2+^ crosslinking. This material is intended to present mechanical stability, osteogenic activities, and anti-inflammatory activity. The results showed that the PLLA microspheres presented homogeneity in the alginate scaffold, which triggered a controlled drug release, improving the anti-inflammatory effects. *In vitro*, cell analysis indicated good proliferation and mineralization of osteoblastic cells, and cross-linking with Sr^2+^ improved the material’s mechanical properties and osteogenic activities.

Ariaudo et al. (2023) developed alginate microsponge scaffolds for administering a therapeutic peptide (CIGB814) for the treatment of rheumatoid arthritis. This biomaterial showed a loading capacity of 80% and a sustained release of peptides through partial erosion of the scaffold. Furthermore, the alginate’s edible and biocompatible capacity opens up new possibilities for new generations of carriers for the controlled administration of peptide drugs, exploring alternative routes to intravenous administration.

Mohammadpour et al. (2021) developed a hydrogel based on alginate and nHAp loaded with purified phenolic extracts from *Linum usitatissimum L.* as a scaffold for bone tissue engineering. The nHAp impregnation was synthesized using the precipitation technique and incorporated into the alginate hydrogel employing physical cross-linking. This material showed an interconnectivity of pores, with a porosity of 80%–90%, with an average size in the range of 100–200 μm. It also showed a 90% release of the drug in the first 12 h, followed by a controlled release over 48 h. *In vitro* analyses showed that the hydrogel has antioxidant activity, promoting bone regeneration. Viability/proliferation analyses confirmed biocompatibility, inducing proliferative effects in a dose-dependent manner.

#### 3.3.4 Collagen/gelatin

He et al. (2024a) studied mineralized collagen (MC) porous scaffolds, producing both blended-matrix scaffolds (He et al., 2024a) as also single components matrices (He et al., 2020). For their pure MC scaffolds, they studied the incorporation of PLGA microspheres encapsulating two synthetic antibacterial peptides, Pac-525 or KSL-W for application in the repair of infectious bone defects. In addition to presenting an MC structure incorporated with microspheres, it also obtained characteristics in cell growth and antibacterial properties, presenting excellent biocompatibility, osteogenic activity and a controlled release of the antibacterial agents in the long term.

Najafloo et al. (2021) developed a niosomal nanocarrier incorporated into a collagen/β-tricalcium phosphate (β-TCP) scaffold for the sustained release of thymol as a natural antibacterial agent, as a bone substitute for the treatment of osteomyelitis. The scaffold showed a thymol release profile where 66% of the drug was released over 30 days, as well as significantly higher cell viability values when compared to the control sample. The antibacterial activity results showed that the scaffolds containing thymol had greater antibacterial efficacy against gram-positive and gram-negative bacteria.

Zhang Y. et al. (2024) developed multifunctional scaffolds simulating natural bone nanostructures by incorporating silver nanowires (AgNWs) into a hierarchical intrafibrillar mineralized collagen matrix/AgNWs for the treatment of infectious vertical bone defects. A concentration of 0.5 mg mL^−1^ AgNWs was used, balancing the biocompatibility and antibacterial properties of the material. The intrafibrillar MC matrix/AgNWs scaffolds showed excellent biocompatibility and osteoinduction, as well as antibacterial and anti-inflammatory properties, due to the characteristics of the AgNWs.

Iimaa et al. (2019) developed a hydrogel as a biomaterial base for the artificial bile duct that can replace tissue without any risk of infection. Initially, the antibacterial agent Finibax, used for the clinical treatment of biliary tract infections, was immobilized in gelatin using a cross-linking agent, and the drug-gelatin hydrogel was prepared as a 3D scaffold. Analyses were carried out using subcutaneous implantation in rats, and it was considered viable for use in tissue engineering applications due to its good bacterial capacity, cell adhesion, and proliferation, as well as its lack of cytotoxicity for cells.

#### 3.3.5 Starch

Esfahani et al. (2022) developed a two-stage study. Initially, implants composed of different types of starch in combination with glycerol monostearate were investigated, to identify the best type of starch for the development of an implant carrying antimalarial agents (artesunate and artemether). This pre-study demonstrated that the system composed of starch with a high amylose content and glycerol monostearate formed a controlled release system, providing a sustained release of artemether over 6 days, the desired time for the treatment of severe cases of malaria. In addition, the starch-based implant showed appropriate mechanical properties when produced.

Subsequently, Esfahani et al. (2023a) deepened the study through *in vivo* and *in vitro* characterizations, also carrying out release kinetics studies through 3D reconstruction of the implants, comparing them with previous versions. The developed subcutaneous implants showed no signs of inflammation or adverse effects at the implantation site in any mice and were completely degraded, proving the potential of these biodegradable and biocompatible starch-based implants.

In another study, Esfahani et al. (2023b) developed a starch-based implant for the controlled release of nimodipine for use in antispasmodic and neuroprotective therapies in the brain. The implants, prepared by hot melt extrusion, were loaded with 20% and 40% nimodipine, and the analyses carried out confirmed the stability and homogeneity of the system developed. The *in vitro* release study demonstrated sustained release of nimodipine for more than 3 months at both concentrations. The works of Estahani et al. provided alternatives for parenteral devices previously produced with synthetic polymers with undesirable biodegradation side effects.

Starch-based implants have also been used to treat cancer. Lee (2022) developed an injectable needle-shaped starch implant loaded with indocyanine green for a photothermal treatment of tumors. The choice of this format is due to the fact that direct intratumoral administration allows the drug to act on the tumor, minimizing systemic absorption and side effects. However, the treatment faces the challenge that high interstitial fluid pressure makes it difficult to retain drugs inside the tumor, so the use of needle-shaped can overcome the pressure and make the drug remain in the tumor. The implant produced a hydrogel structure after absorbing water, facilitating application and providing sufficient strength to be injected into certain areas, as well as demonstrating effective cytotoxicity and anti-cancer effects. *In vivo*, the needle-shaped implant performed continuous drug delivery to the tumor effectively and uniformly, demonstrating the advantages of needle-shaped implants and their effectiveness in treating tumors.

Finally, Saboktakin et al. (2012) developed a starch-based hydrogel using carboxymethyl starch and dextran sulfate to encapsulate a porphyrin-based photosensitizing agent for cancer treatment. The combination of carboxymethyl starch and dextran sulfate improved the encapsulation and stability of the drug, allowing controlled release at the desired sites. The authors suggest that hydrogels can be applied to cancer treatment, showing controlled release at tumor sites, followed by degradation of the hydrogel and release of the drug.

These studies present starch-based systems as emerging versatile platforms for sustained and effective drug delivery, demonstrating promising results across various therapeutic applications, as a biodegradable alternative to synthetic polymers.

#### 3.3.6 Devices based on blended biomacromolecules

For their scaffold blended version, He et al. (2024b) associated the mineralized collagen (MC) with polycaprolactone loaded with bi-layer microspheres with antibacterial and osteogenic functions. The microspheres were composed of PLGA and CHI and were loaded with bone morphogenetic protein-2 (BMP-2), an important osteogenic growth factor with biological activities in osteogenesis and proliferation. For long-term antibacterial activity, Pac-525 was used, a synthetic amino acid sequence with broad-spectrum antibacterial activity and low drug resistance. The PLGA (BMP-2)/CHI (PAC-525) composite microspheres were manufactured using electrospinning and emulsion crosslinking methods. The scaffolds had a porous structure with porosity and pore diameter similar to cancellous bone. The release curve of the microspheres showed a two-stage release of Pac-525 and BMP-2 over 30 days, as well as antibacterial activity, inhibiting *S. aureus* and *E. coli* and promoting alkaline phosphatase activity.

Lee et al. (2004) produced blended collagen/chitosan porous scaffolds by a freeze-drying method in the presence of chondroitin sulfate, a glycosaminoglycan component. Transforming growth factor-beta1 (TGF-β1) was incorporated into chitosan microspheres using an emulsion-crosslinking method; the microspheres were then encapsulated into the scaffold using dispersion by ethanol. The scaffold was populated with chondrocytes, and both proliferation rate and glycosaminoglycan production were significantly higher in the presence of the TGF-β1 microspheres, besides a higher observation of ECM and collagen II, highlighting the potential of these bioactive scaffolds to enhance cartilage formation.

Nair et al. (2015) developed a hydrogel for nucleus pulposus (NP) tissue engineering, produced from chitosan-poly(hydroxybutyrate-co-valerate) with chondroitin sulfate (CS) nanoparticles, without using a crosslinker. The use of CS is because this material has great potential for increasing the biocompatibility of hydrogels, as well as being able to electrostatically bind to growth factors and induce cell differentiation. The hydrogels can withstand variable stresses corresponding to daily activities such as lying down (0.01 MPa), sitting down (0.5 MPa), and standing up (1.0 MPa) under dynamic conditions, as well as being stable for 2 weeks without altering their mechanical properties. In addition, they were able to aid the viability and adhesion of adipose tissue-derived rat mesenchymal stem cells, and the presence of CS nanoparticles significantly increased the viability and chondrogenic differentiation of MSCs.

Concheiro and Alvarez-Lorenzo (2013) reviewed chemically cross-linked and grafted cyclodextrin hydrogels to produce drug-eluting medical devices with different crosslinkers and hydrophilic polymers and polyelectrolytes, whereas Barroso et al. (2014) prepared chitosan–collagen crosslinked scaffolds for drug delivery purposes and coated them with poly(N,N′-diethylacrylamide) to confer a thermoresponsive behavior. Ibuprofen and a model protein (bovine serum albumin) were loaded in the biomacromolecular matrix during casting.

Cheng et al. (2020) produced bioactive blended electrospun fibers for improved anti-infection properties and osseointegration in bone therapy. The fibers were produced with a mixture of gelatin, PLA, and AgNP. The nanoparticles acted as nucleation sites for mineralization and provided high antibacterial effects against the four studied bacteria strains - *M. albicans, E. coli, S. aureus, and P. aeruginosa*. Bone-marrow MSCs adhered and proliferated well on the fibers’ surface.

Zarrintaj et al. (2023) reviewed neuro conduits based on different biopolymers (chitosan, gelatin, collagen, cellulose, and PLA), in which active substances and soft tissues were loaded for neural repairing, e.g., alpha-lipoic acid, bone marrow, tricalcium phosphate, cerebrospinal fluid, Schwann cells and pyrroloquinoline quinone. They presented diverse outcomes, such as improving the nerve repair process, bridging long gaps, mimicking ECM, increasing motor function, and secreting bioactive biomolecules.

Table 5 summarizes the therapeutic functions achieved by implants with different biopolymeric matrices and bioactive agents.

**TABLE 5:** Therapeutic functions of biomacromolecular devices.

Device matrix	Cellulose-based (cellulose, nanocellulose, bacterial cellulose, modified celluloses)	Chitosan	Alginate	Collagen	Starch
Application site	Tumor tissue; bone tissue; canthoplasty; dental; duraplasty; periodontal pockets	Devices coating; periodontal hard and soft tissue; ophthalmic biomaterial	Joints; cartilage; bone	Bone	Subcutaneous and other parenteral depot systems; intratumoral
Application form	Cellulose nanofiber scaffolds; hydrogels; aerogels; membranes	Films, particles, gels, scaffolds, membranes, sponges	Scaffold; microsponge; hydrogel	Porous scaffold; niosomal nanocarrier	Extruded cylinder; needle-shaped hydrogel
Therapeutic agent	Doxorubicin; curcumin; TiO_2_; AgNP; ZnO; HAp; CHX; GFs	Cu/Al; tetracycline; TGF; BMP-6; GP	TGF; ibuprofen; therapeutic peptide (CIGB314); nHAp; phenolic extracts	Antibiotics (Pac-525, KSL-W), thymol)	Artesunate and artemether; nimodipine; indocyanine green; porphyrin
Therapeutic effect	Cancer treatment; antibacterial activity	Antibacterial; mineralization and osteoinduction; anti-inflammatory	Osteoinduction; cartilage regeneration; mineralization; arthritis combat; antioxidant	Repair infectious bone defects, antibacterial properties, osteogenic activity, osteomyelitis treatment	Malaria; antispasmodic and neuroprotective; cancer therapies
Reference	Tabary et al. (2014), Rakib Hasan Khan et al. (2022), Tarrahi et al. (2021), Ashraf et al. (2020), Heydari et al. (2021), Sknepnek et al. (2024), Yang et al. (2023), Inoue et al. (2020), Stumpf et al. (2020)	Sanpo et al. (2009), Shen et al. (2008), Soran et al. (2012), Li et al. (2019b), Lai (2012)	Yoon et al. (2018), Mata et al. (2017), Zhao et al. (2022), Ariaudo et al. (2023), Mohammadpour et al. (2021)	He et al. (2024a), He et al. (2020), Najafloo et al. (2021), Zhang Y. et al. (2024), Iimaa et al. (2019)	Esfahani et al. (2022), Esfahani et al. (2023a), Esfahani et al. (2023b), Lee (2022), Saboktakin et al. (2012)

### 3.4 Bioceramic-like materials

Metal surfaces have been modified to adjust roughness and improve bone-implant contact to accelerate osseointegration through several physical and fewer chemical methods, such is often the case with titanium; however, ceramics possess a high hardness that hinders several of these processes. Nonetheless, surface treatment processes such as mechanical grinding, physical and chemical modifications, and applications of hard (Sherman et al., 2008) or soft coatings have been successfully employed in these materials; using techniques such as plasma-based methods (Khelifa et al., 2016; Nathanael and Oh, 2020) sandblasting, acid etching, or application of calcium, phosphate, bisphosphonate, or collagen layers (Prakash et al., 2021). Although these treatments may produce positive effects in terms of osseointegration, for example, some studies claim they tend to create small defects that may lead to structural failure upon stress. Although coatings for all-ceramic implants are not fairly common and soft coatings are even more scarce, these treatments have been explored in matrices such as calcium phosphate, hydroxyapatite, and zirconia.

#### 3.4.1 Calcium phosphate

Some studies investigated using hard coatings for ceramic implants based on nanoceramics. For instance, Mangaraj et al. (2024) developed a new biomaterial by incorporating zinc oxide (ZnO) into β-TCP scaffolds using wet chemical techniques, to overcome mechanical strength limitations and promote bone growth in β-TCP scaffolds. The authors reported that adding ZnO significantly improved the biophysical properties, increasing cell adhesion and proliferation with higher concentrations of ZnO. These new materials also showed an ability to promote bone growth and regeneration, due to the formation of bone-like apatite.

To the same end, Filipov et al. (2024) also developed TCP-HAp scaffolds with antimicrobial interfaces, using ZnO layers. Using pulsed laser deposition of ZnO, micropatterns were created and the surface roughness of the materials was altered. It was observed that metabolic activity was reduced and cell morphology was impaired in the presence of ZnO, indicating that a ZnO surface could provide an antimicrobial interface for implants used in bone regeneration.

Feng et al. (2014) developed a β-TCP scaffold by selective laser synthesis and improved its mechanical and biological properties by doping it with ZnO. The group observed that a concentration of 2.5% ZnO led to an increase in compressive strength and toughness; however, higher concentrations resulted in a decrease in these properties, possibly due to the size of the ZnO particles. The scaffolds with 2.5% ZnO also showed excellent cell adhesion and proliferation, as well as the ability to form apatite when incubated in SBF solution, suggesting their potential for osteoinduction and osteoconduction.

#### 3.4.2 Hydroxyapatite

Other authors managed to produce soft and composed coatings. Using a mesoporous hydroxyapatite scaffold as the implant matrix, Yu et al. (2021) developed a coating using ursolic acid-loaded chitosan. This allowed the device to be applied in the treatment of bone defects due to the anti-inflammatory effects of ursolic acid. The resulting material showed controlled release due to its nanometric pore sizes, which led to inhibiting macrophage polarization toward pro-inflammatory macrophages (M1 type), and promoting the expression of osteogenesis-related genes. The activated scaffolds showed anti-inflammatory, osseointegration, osteoinductivity, and bone regeneration characteristics.

Similarly, Zhongxing et al. (2021) produced a 3D-printed porous hydroxyapatite scaffold with surface-specific binding of peptides for conferring to it antibacterial and osteogenic ability. HAp binding domain was linked to the C-terminal of bone morphogenetic protein 2 mimetic peptide and the PSI10 antimicrobial peptide. The BMP significantly improved the gene expression and protein translation levels of type I collagen, osteocalcin (OCN), and Runx2, while PSI10 promoted strong antibacterial inhibition against *S. aureus* and *E. coli* in the grafted devices.

Similarly, Heidari et al. (2020a) investigated ZnO particles in HAp scaffolds. These HAp/ZnO biomaterials had higher compressive strength, fracture toughness, and density, although lower hardness when compared to pure HAp scaffolds. In addition, the scaffolds showed an ability to promote apatite on their surface when immersed in simulated body fluid solution, resulting in a rougher and rougher surface. *In vitro* analyses revealed that the scaffolds had biocompatibility and adequate alkaline phosphatase activity, making them promising for application in bone regeneration.

Heidari et al. (2020b) also developed a HAp/ZnO/Palladium (Pd) scaffold focusing on its mechanical, antibacterial, biocompatibility and bioactivity properties. The results indicated that the scaffolds had greater compressive strength and toughness compared to pure HAp scaffolds, as well as showing the formation of apatite on the surface. However, although they showed antibacterial activity in deactivating microorganisms *in vitro*, the biocompatibility tests indicated lower cell proliferation, which was attributed to the concentration of ZnO and Pd used, which seems to have an adverse effect on cell proliferation.

#### 3.4.3 Zirconia

Zirconia (ZrO_2_) ceramics with high mechanical properties have been used as load-bearing implants in various surgical areas. Aiming to improve the mechanical and bioactive properties of these materials, Sadiasa et al. (2013) coated a biphasic calcium phosphate/ZrO_2_ scaffold with a PLGA/biphasic calcium phosphate composite with incorporated simvastatin. This coated material showed a significant increase in resistance compared to its original matrix. In addition, with the increase of PLGA in the coating composition, there was a decrease in porosity, degradation rate, and weight loss of the scaffolds after 4 weeks. The release of simvastatin proved to be sustained and the material showed improved biocompatibility, making it a promising approach for bone regeneration.

In a related study, Shi et al. (2016) developed a porous bioceramic artificial vertebral biomaterial based on HAp/ZrO_2_, which contained a CHI hydrogel associated with recombinant human bone morphogenic protein-2 (rhBMP-2) controlled release for bone defect repair. In *in vivo* analysis (Beagle dogs), the material showed an encapsulation rate and drug load of approximately 92% and 40 ng/mg, respectively. At 24 weeks post-operation, it was observed that the scaffold implanted in group A (artificial vertebral biomaterial/rhBMP-2) had completely fused with the host bone, showing a significantly greater newly formed bone volume than in group B (non-drug-loaded sample), as well as showing greater resistance to compression. It was also observed that the artificial vertebral biomaterial of group A had been filled with almost mature bone, structuring itself similarly to a trabecular structure, which was not apparent in Group B. These results show that the biomaterial can promote the repair of bone defects and induce the growth of bone tissue in the pores.

Khoshaim et al. (2024) also developed a nanocomposite with different concentrations of HAp, cordierite, and ZrO_2_. It was observed that lowering the sintering temperature and increasing the concentration of ZrO_2_ increased the porosity of the nanocomposites. In addition, increasing the concentration of ZrO_2_ and cordierite increased microhardness and compressive strength, although electrical conductivity decreased. However, the increase in porosity and the presence of Mg^2^⁺ ions in the cordierite composition reduced bioactivity as the concentration of ZrO_2_ and cordierite increased. Even so, all the samples exhibited a strong antibacterial effect against the bacterium *Staphylococcus epidermidis*.

Desante et al. (2023) proposed a functionalization method of inert ceramic (zirconia) implant surfaces with calcium phosphate and antibiotic-loaded degradable polymer nanoparticles for biomimetic and antibacterial effects. Gentamicin or bacitracin was loaded on PLGA NPs, created by the double emulsion and solvent evaporation method. The NPs were then coprecipitated within the phosphates (hydroxyapatite and octacalcium phosphate) coating in a layer deposited over ZrO_2_.

#### 3.4.4 Bioglass

Bioglass coatings were also explored, for instance, Ding et al. (2021) investigated the incorporation of ZrO_2_ into calcium silicate (CaSi) ceramics and evaluated their mechanical properties, long-term stability, *in vitro* osteogenic activity, and antibacterial capacity. It was observed that the three-point flexural strength of the CaSi-ZrO_2_ samples exceeded that of cortical bone and that their flexural strength was very close to that reported for cortical bone. In addition, biomaterials with a higher CaSi content significantly increased cell growth, differentiation, and mineralization of hMSCs. The composites with the highest CaSi content also showed greater antibacterial activity against *E. coli* and *S. aureus*.

Qiu et al. (2022) developed a scaffold from borosilicate bio-glass and chitosan, whose surface was modified with ZnO NPs. The results showed that the scaffold had good degradation and osteogenic properties, as well as improved antibacterial properties provided by the ZnO, indicating its potential for future applications in bone regeneration.

### 3.5 Composite and hybrid materials implants

Due to the nature of composites consisting of combined materials of different categories, many of the materials cited within this review fall into this category once a biomacromolecular system is incorporated for loading or releasing biofunctional components. However, this section will specifically cover materials whose original matrices were already composed of different categories of substances, regardless of the biomacromolecular system incorporated.

#### 3.5.1 Macromolecular-based composites

Wang et al. (2021b) developed a chitosan-based scaffold for use in bone tissue engineering incorporating either natural diatomite or modified diatomite, which was synthesized by grafting polyethyleneimine (PEI) onto the surface of diatomite via hydroxyl groups. The scaffold showed an improvement in its properties with the incorporation of PEI, the protein absorption capacity and cytocompatibility of modified diatomite were improved compared to natural diatomite, as well as its mechanical strength. The growth factor rhBMP-2 was incorporated to study the possibility of its controlled release, resulting in a positive impact on the proliferation and osteogenic differentiation of bone mesenchymal stem cells, and indicating the scaffold’s capacity for bone regeneration.

Changing both the biomacromolecule and the calcium origin, Wong et al. (2023) developed alginate and cockle shell powder bone nanobiocomposites loaded with pure ciprofloxacin and tested its encapsulation and drug release as well as antimicrobial properties with bacterial strains of *S. aureus* and *P. aeruginosa*. Although the results showed a low encapsulation and drug release performance, bacterial inhibition studies showed some inhibitory effects on the growth of both strains tested. An additional mineralization study was also performed and indicated that the characteristics of the scaffolds were not compromised by the addition of the drug, providing some insights into the potential use of the scaffold for applications in bone regeneration and drug delivery.

Zhang and Zhang (2002b) developed macroporous chitosan scaffolds reinforced by β-tricalcium phosphate (β-TCP) and calcium phosphate invert glass. These scaffolds were designed to act as drug carriers for controlled release in bone regeneration applications against osteomyelitis. The drug used was the antibiotic gentamicin sulfate (GS). It was observed that, compared to pure chitosan scaffolds loaded with GS, the initial release of GS was decreased by incorporating β-TCP, but it was possible to obtain a release for more than 3 weeks. Furthermore, in cell morphological analyses (MG63 cell line), there was no apparent differentiation for the cultured cells, and there was also growth and migration towards the scaffolds, suggesting good cell biocompatibility of the composite scaffolds.

Following the tendency of inorganic-reinforced biopolymeric implants, Buranapanitkit et al. (2004) presented a skeletal drug delivery composite system composed of chitosan with hydroxyapatite and calcium sulfate hemihydrate (plaster of Paris). In sequence, different antibiotics (vancomycin, fosfomycin, or sodium fusidate) were used to impregnate a HAp/plaster of Paris powder mixture. Then, the powder was dispersed into a chitosan gel and the resulting mixture was cast in a mold, forming a cement tablet after drying. The drug delivery system was able to combat methicillin-resistant *Staphylococcus aureus* through antibiotic release, and the produced cement constituted a local biodegradable delivery system that could be applied to treat chronic osteomyelitis, especially with the antibiotics vancomycin and fosfomycin.

Bhowmick et al. (2018b) also designed a biomimetic nanocomposite for bone tissue engineering. A chitosan matrix was used to support both organically modified montmorillonite clay and a blend of hydroxyapatite and zinc oxide. They observed strong antibacterial properties against different bacteria strains aligned with the proliferation of osteoblastic MG-63 cells, which were enhanced due to the presence of the modified clay. On the other hand, Ng et al. (2022) produced 3D-printed scaffolds made of a composite of self-assembled photo-crosslinkable methacrylate SF with hollow mesoporous silica microcapsules, in which they incorporated a fluoroquinolone antibiotic (ciprofloxacin) able to produce antibacterial action through its sustained release. Besides this effect, the devices presented osteoconductive, -inductive properties due to the presence of the mesoporous silica particles, promoting osteoblastic differentiation by inducing the expression of osteogenic markers and matrix mineralization.

Ardakani et al. (2022) developed a polyvinylidene fluoride hybrid scaffold incorporating zinc oxide (ZnO) nanorods and PCL nanofibers containing chitosan nanoparticles loaded with dexamethasone (DEX). It was observed that the addition of 3% ZnO nanorods and PCL with 0.8% chitosan nanoparticles loaded with DEX was suitable for cell adhesion and proliferation, as well as showing potential for cell differentiation. The mechanical properties achieved also indicated that this material could be a promising option for bone regeneration.

Although more common in bone applications, composites have attracted attention in other fields. Radmansouri et al. (2018), for example, studied doxorubicin hydrochloride-loaded electrospun chitosan/cobalt ferrite/titanium oxide nanofibers’ effect on hyperthermia and chemotherapy against melanoma cancer B16F10 cell lines. Chitosan was used as the matrix for both drug and particle incorporation. The cobalt ferrite nanoparticles were synthesized via a microwave heating method. The titanium oxide nanoparticles were mixed with cobalt ferrite to control the rise in tissue temperature. The work demonstrated the produced material can be applied externally for localized cancer therapy. Similarly, Xu et al. (2019b) produced membranes and artificial bone coatings, using electrostatically assembled chitosan/alginate multilayers deposited by the LbL technique to encapsulate fulvestrant-loaded modified silica nanocapsules. Fulvestrant is an FDA-approved selective estrogen receptor down-regulator agent used for the treatment of breast cancer. The encapsulation allowed for a pH-controlled release of the drug in pHs similar to the ones found in ill tissues.

#### 3.5.2 Metal-based composites

In addition to the materials presented in Section 3.1, other composites were prepared based on metal alloy matrices. Karacan et al. (2019), for example, coated Ti6Al4V discs with Poly-Lactic Acid (PLA) containing Gentamicin (Gm) antibiotic-loaded coralline hydroxyapatite (HAp) and demonstrated their capability to combat post-operative infections, preventing bacteria from growing in the alloy surface. They also demonstrated the PLA coating uniformly distributed the components on the device surface and could survive handling and insertion. Similarly, Mohan Raj et al. (2018b) produced TiO_2_-SiO_2_ mixtures on a Ti alloy by anodization method and coated the composites with a blend of chitosan-lysine biopolymers by an electrodeposition method. Gentamicin sulfate was loaded on the biopolymers as a model drug. The composite allowed a good adhesion and growth of osteoblasts, while the loaded Gm promoted the combat of osteomyelitis, and its combination with the composite promoted the repair of the bone defect initiated by the infection.

## 4 Viability assays for implants

Analyzing biomaterials’ viability for implants requires a comprehensive approach considering physicochemical, biological, and mechanical properties. Corrosion and degradation tests are essential to evaluate the chemical stability and degradation rate of bioabsorbable materials, particularly in simulated physiological media (Mei et al., 2020; Dong et al., 2021; Luo et al., 2014). These analyses monitor mass and ionic composition changes over time, indicating the material’s durability in biological environments. Additionally, antimicrobial tests assess the ability of biomaterials to inhibit the growth of microorganisms such as *Staphylococcus aureus* and *Escherichia coli*, using methods like the inhibition zone and colony count (Pańtak et al., 2024; Ibrahim et al., 2024; Agrawal and Minhas, 2024; Wei et al., 2024).

Cytotoxicity assays, such as MTT (Dornelas et al., 2024) or Alamar Blue (Podgórski et al., 2022), are widely used to investigate the cellular compatibility of biomaterials, ensuring they do not exhibit toxic effects on human or animal cells. From a mechanical perspective, compression tests provide insights into the structural resistance and load-bearing capacity of the material, which are critical parameters for bone implants (Bakhtiari et al., 2023). Simultaneously, the controlled release profile of therapeutic agents embedded in the biomaterial, such as drugs or growth factors, is evaluated to ensure clinical performance efficiency (Oliveira et al., 2021; Bhatnagar et al., 2022).

Detailed surface characterization is equally important as surface properties (e.g., composition or roughness) influence cellular interaction. Techniques such as scanning electron microscopy (SEM) allow high-resolution analysis of surface morphology, while spectroscopies like FTIR and XPS provide information on chemical composition and functional groups. The degree of crystallinity is investigated using X-ray diffraction (XRD), whereas roughness, evaluated with profilometers or AFM, is correlated with cellular adhesion (Jayawardena et al., 2021; Ali et al., 2023; Kravanja and Finšgar, 2021). These integrated analyses ensure a thorough understanding of biomaterials’ performance and viability for specific applications.

## 5 Outlook

Biomacromolecules offer specific advantages for the incorporation of therapeutic agents into implants, as they have functional groups capable of interacting with these compounds (e.g., growth factors, antibiotics, drugs, nanoceramics), serving as effective carrier agents to allow controlled drug release or specific biological action at the target site.

### 5.1 Metals

The application of biomolecules in metallic implants represents an innovative strategy that enhances the mechanical properties (against interfacial loosening) and biointegration of medical devices. Incorporating biopolymer coatings, such as CHI and SF, into metal alloys, such as SS, titanium, and magnesium, not only improves corrosion resistance but also facilitates bioactivity and cell adhesion (Yu et al., 2020; Zhou et al., 2021; Ramskogler et al., 2016b). These coatings act as interfaces between the metal and biological tissue, promoting a favorable environment for bone regeneration and implant integration (Elyada et al., 2014). Furthermore, the use of layered deposition techniques, such as LbL, allows for precise modification of surface properties, incorporating growth factors and antimicrobial agents that further increase the effectiveness of the implant (Păun et al., 2023; Jensen et al., 2022; Zhou et al., 2021).

Additionally, biomolecules provide properties that help mitigate complications associated with traditional metal implants (Chacón et al., 2022; Jensen et al., 2022; Johnbosco et al., 2021). For example, the ability of some biopolymers to release drugs, such as antibiotics, in a controlled manner can reduce bacterial colonization in implants, minimizing the risk of perioperative infections (Jensen et al., 2022). Research into antibacterial coatings, particularly those that combine antimicrobial agents with biopolymers, demonstrates promising results in protecting implants against common pathogens (Yu et al., 2020; Jensen et al., 2022; Zhou et al., 2021). Thus, the use of biopolymers not only improves the mechanical performance of metallic implants but also promotes a more harmonious interaction with biological tissues, facilitating healing and reducing clinical complications.

### 5.2 Synthetic polymers

The continuous development of polymers for medical implants presents promising prospects as new technological and biomedical advances emerge. Polymer engineering is increasingly focused on “smart” and adaptive materials that can respond to specific stimuli from the human body, such as changes in pH, temperature, or even biochemical signals, paving the way for more effective and less invasive therapeutic solutions (Li et al., 2015b; Bratek-Skicki, 2021; Balcerak-Woźniak et al., 2024).

### 5.3 Biopolymers

Biopolymers are excellent options for use in implantable materials due to their characteristics, such as biocompatibility, biodegradation, and non-cytotoxicity (Rebelo et al., 2017), often allowing devices based on this type of matrix to be gradually absorbed by the body without requiring surgery for their removal. The ability of biopolymers to form scaffolds that facilitate cell adhesion and proliferation is also noteworthy, as this helps tissue regeneration around the implant site. Moreover, the versatility of biopolymer properties, in addition to the possibility of structural modification and combination between them to obtain desired properties, allows researchers to adjust the release profiles of the therapeutic agent to the specific demands of an application. In a manner in which several recent studies focus on an effort to match these substances rather than focusing on a single biopolymeric matrix (Fredi and Dorigato, 2024). This trend is observed due to the ability of blended systems to enhance the properties of individual components, such as adjusting degradation rates, increasing mechanical strength, or biological functionality when adding a bioactive compound, improving the versatility of these systems for diverse biomedical applications.

In addition, the creation of biopolymer composites by the incorporation of nanoparticles or other additives increases their therapeutic potential. For example, studies demonstrated that the combination of biopolymers with nanoparticles such as silver or zinc oxide can impart antibacterial properties to scaffolds (Ashraf et al., 2020; Sknepnek et al., 2024; Yang et al., 2023), while materials such as hydroxyapatite can promote osseointegration in bone therapy.

### 5.4 Bioceramics

Despite the recent use of bioinert and bioactive ceramics as implants and medical devices, there is a recent trend of bioabsorbable ceramics application in tissue engineering of calcified tissue, highlighting its regeneration efficiency (Shanmugam and Sahadevan, 2018). Zirconia dental all-ceramic implants are slowly gaining importance in the dentistry market, which is still led by titanium-based materials due to a lack of long-term efficacy studies, competitive costs, and professional schooling choices for this all-ceramic material (Bansal et al., 2022; Thiem et al., 2022b). However, implant composites with incorporated nanoceramic materials (such as calcium phosphate and hydroxyapatite) are frequently observed for bone and dental applications. The nanoceramics interact better with cells, generating a better regeneration of calcified tissue. They act on osteoblasts and osteoclasts, resulting in the maintenance of calcified tissue and improvement of the performance of the orthopedic/dental implant (Shanmugam and Sahadevan, 2018).

### 5.5 Composites

Composite technology in implants is highly promising as it combines organic and inorganic materials of different categories to address the limitations of traditional materials while introducing new functions. In composites, the organic matrix (e.g., biomacromolecules) is frequently used as a carrier of bioactive molecules such as growth factors and antibiotics, enhancing healing and infection control, as they can also function as binders or substrates for the inorganic phase. Biocomposites use biocompatible materials that can be applied to living tissues due to low toxicity, biodegradability, and high biocompatibility (Valente et al., 2020). In these materials, the inorganic phase provides structural stability or nuclei for biomineralization; oxides such as hydroxyapatite, can enhance biomineralization for faster bone regeneration (Elyada et al., 2014), whereas metal and alloys can act as the substrate for higher mechanical properties. However, significant challenges remain, osseointegration, cell proliferation, and biocompatibility - from a biological point of view - are some of the main obstacles in pursuing an ideal material for human bone implants.

## 6 Future trends

### 6.1 Metals

Combining biopolymers with metal alloys holds great promise for therapeutic applications, especially in orthopedic, dental, and cardiovascular fields. In the future, the application of biomolecules in metallic implants is expected to advance toward creating functionalized surfaces that release drugs and molecules to promote tissue regeneration, reduce infection risk, and increase implant longevity. These advancements should also focus on developing implants with biomolecules that support self-regeneration and adapt to changes in the body. Therefore, the pursuit of more durable and functional technologies is essential for the next-generation of medical devices.

### 6.2 Synthetic polymers

One of the areas with the greatest potential for expansion is the combination of synthetic polymers with fabrication techniques, such as 3D printing and layered production. These processes allow the creation of personalized implants, adjusted to the anatomical and functional needs of each patient, increasing the chances of clinical success. The functionalization of polymers with biomolecules also tends to gain prominence, allowing greater control over tissue regeneration and the integration of the material with the body. Furthermore, the incorporation of controlled drug release technologies, particularly with the use of biodegradable polymers, will continue to play a crucial role in improving treatments for chronic diseases and bone regeneration (Jana et al., 2021).

Future challenges include optimizing these materials’ mechanical and biocompatible properties to ensure greater durability and minimize complications, such as inflammation or rejection. As research advances, the expectation is that new polymers and combinations with nanomaterials and biomacromolecules will be able to offer greater longevity and functionality, significantly reducing the need for replacement and maintenance surgeries. In this way, the application of synthetic polymers in medical implants tends to evolve towards more personalized, efficient, and safe solutions, transforming clinical practice. This scenario highlights the possibilities for the development of “smart” biomaterials that respond to changes in the body, such as pH, temperature, or biochemical signals. These materials could provide dynamic and adaptive therapeutic responses, such as releasing drugs when inflammation or infection is detected.

### 6.3 Biopolymers

The biopolymers’ versatility of combinations allows them to be tailored for specific medical applications, from bone regeneration to drug delivery, presenting a promising approach for improving the efficacy and functionality of different biomedical devices. Future research will likely continue to explore the functionalization of the devices with bioactive molecules, such as antibiotics, growth factors, and other therapeutic agents, especially by integration with biopolymers. A major trend is observed for developing targeted and sustained drug delivery systems within implants, allowing more precise release profiles tailored to individual patient needs. It is equally expected that implants that degrade and are absorbed by the body over time will be favored compared to their counterparts, eliminating the need for surgical removal. For this reason, research will focus on biodegradable materials capable of maintaining mechanical strength during the healing period.

### 6.4 Bioceramics and composites

The incorporation of nanoceramics and the use of composites in implants have demonstrated great potential in improving both the mechanical and biological properties of devices and will likely expand. Personalized medical solutions, including materials and designs to meet each patient’s specific clinical needs, are set to instigate growth and innovation within the biomedical field, especially regarding implantable devices. Despite advancements in composites and a high predominance of nanoceramics such as nanohydroxyapatite or ZrO_2_ in bone and dental implants (Nasar, 2019), there is no single “ideal” material, as the complex biological environments and patient-specific factors make a one-size-fits-all solution impractical (Vazquez-Silva et al., 2022). With the integration of nanohydroxyapatite and innovations in drug delivery, the opportunities over the coming few years will involve the development of composite implants for therapeutic applications, providing systems for controlled drug delivery and patient-specific design to improve biological and mechanical performance. Composite implants will play a central role in the future of personalized regenerative medicine. The synergistic combination of organic and inorganic phases addresses the limitations of traditional implants, such as low biocompatibility and mechanical strength, while complementing them with therapeutic functionalities such as controlled drug release or infection prevention.

## Abbreviations

3D, three-dimensional; AgNWs, silver nanowires; AP, antimicrobial peptide; BC, bacterial cellulose; BMP, bone morphogenetic protein; CD, cluster of differentiation; CF, carbon fiber; CHI, chitosan; CHX, chlorhexidine; CS, chondroitin sulfate; DOX, doxorubicin; ECM, extracellular matrix; Gm, gentamicin; GFs, Growth factors; GP, genipin; HA, hyaluronic acid; HAp, Hydroxyapatite; IOL, Intraocular lens; LbL, Layer-by-Layer; MC, mineralized collagen; MSCs, Mesenchymal stem cells; nHAp, nano-hydroxyapatite; NPs, nanoparticles; OCN, osteocalcin; PCL, poly(ε-caprolactone); PDMS, polydimethylsiloxane; PE, polyethylene; PEEK, polyether ether ketone; PEG, polyethylene glycol; PEI, polyethyleneimine; PGA, polyglycolic acid; PLA, polylactic acid; PLGA, poly(lactic-co-glycolic) acid; PLLA, Poly-L-lactic acid; PMMA, Poly(methyl methacrylate); PU, polyurethane; SF, silk fibroin; SS, stainless steel; TCP, tricalcium phosphate; TGF, transforming growth factor; UA, ursolic acid; UHMWPE, ultra-high molecular weight polyethylene; VEGF, vascular endothelial growth factor.

## References

[B1] Abdulmajeed A. A. Kokkari A. K. Käpylä J. Massera J. Hupa L. Vallittu P. K. (2014). *In vitro* blood and fibroblast responses to BisGMA–TEGDMA/bioactive glass composite implants. J. Mater Sci. Mater Med. 25, 151–162. 10.1007/s10856-013-5040-0 24022800

[B2] Abka-khajouei R. Tounsi L. Shahabi N. Patel A. K. Abdelkafi S. Michaud P. (2022). Structures, properties and applications of alginates. Mar. Drugs 20, 364. 10.3390/md20060364 35736167 PMC9225620

[B3] Adepu S. Ramakrishna S. (2021). Controlled drug delivery systems: current status and future directions. Molecules 26, 5905. 10.3390/molecules26195905 34641447 PMC8512302

[B4] Agrawal D. Minhas A. P. (2024). “Antimicrobial coatings in dental implants,” in Functional coatings for biomedical, energy, and environmental applications (Wiley), 51–75. 10.1002/9781394263172.ch3

[B5] Ajili S. H. Ebrahimi N. G. Soleimani M. (2009). Polyurethane/polycaprolactane blend with shape memory effect as a proposed material for cardiovascular implants. Acta Biomater. 5, 1519–1530. 10.1016/j.actbio.2008.12.014 19249261

[B6] Akentjew T. L. Terraza C. Suazo C. Maksimuck J. Wilkens C. A. Vargas F. (2019). Rapid fabrication of reinforced and cell-laden vascular grafts structurally inspired by human coronary arteries. Nat. Commun. 10, 3098. 10.1038/s41467-019-11090-3 31308369 PMC6629634

[B7] Albayrak S. Gul C. (2024). Ceramic coatings for biomedical applications. Compos. Sci. Technol., 233–256. 10.1007/978-981-97-3909-7_12

[B8] Ali A. Zhang N. Santos R. M. (2023). Mineral characterization using scanning electron microscopy (SEM): a review of the fundamentals, advancements, and research directions. Appl. Sci. 13, 12600. 10.3390/app132312600

[B9] Ali S. Abdul Rani A. M. Baig Z. Ahmed S. W. Hussain G. Subramaniam K. (2020). Biocompatibility and corrosion resistance of metallic biomaterials. Corros. Rev. 38, 381–402. 10.1515/corrrev-2020-0001

[B10] Alkaron W. Almansoori A. Balázsi K. Balázsi C. (2024). Hydroxyapatite-based natural biopolymer composite for tissue regeneration. Materials 17, 4117. 10.3390/ma17164117 39203295 PMC11356673

[B11] Alsaab H. O. Alharbi F. D. Alhibs A. S. Alanazi N. B. Alshehri B. Y. Saleh M. A. (2022). PLGA-based nanomedicine: history of advancement and development in clinical applications of multiple diseases. Pharmaceutics 14, 2728. 10.3390/pharmaceutics14122728 36559223 PMC9786338

[B12] Al-Shalawi F. D. Mohamed A. A. H. Jung D. W. Mohd Ariffin M. K. A. Seng Kim C. L. Brabazon D. (2023). Biomaterials as implants in the orthopedic field for regenerative medicine: metal versus synthetic polymers. Polym. (Basel) 15, 2601. 10.3390/polym15122601 PMC10303232 37376247

[B13] Ammann K. R. Hossainy S. F. A. Hossainy S. Slepian M. J. (2021). Hemocompatibility of polymers for use in vascular endoluminal implants. J. Appl. Polym. Sci. 138. 10.1002/app.51277

[B14] Andreiotelli M. Wenz H. J. Kohal R. (2009). Are ceramic implants a viable alternative to titanium implants? A systematic literature review. Clin. Oral Implants Res. 20, 32–47. 10.1111/j.1600-0501.2009.01785.x 19663947

[B15] Ardakani F. F. Mohammadi M. Mashayekhan S. (2022). ZnO-incorporated polyvinylidene fluoride/poly(ε-caprolactone) nanocomposite scaffold with controlled release of dexamethasone for bone tissue engineering. Appl. Phys. A 128, 654. 10.1007/s00339-022-05762-z

[B16] Ariaudo D. Cavalieri F. Rinaldi A. Aguilera A. Lopez M. Perez H. G. (2023). Alginate microsponges as a scaffold for delivery of a therapeutic peptide against rheumatoid arthritis. Nanomaterials 13, 2709. 10.3390/nano13192709 37836350 PMC10574729

[B17] Asadi H. Ramasamy R. (2022). Silk-cellulose nanocrystal composite coatings for enhanced corrosion protection and cytocompatibility. ECS Meet. Abstr. MA2022-02, 683. 10.1149/MA2022-0210683mtgabs

[B18] Asadi N. Del Bakhshayesh A. R. Davaran S. Akbarzadeh A. (2020). Common biocompatible polymeric materials for tissue engineering and regenerative medicine. Mater Chem. Phys. 242, 122528. 10.1016/j.matchemphys.2019.122528

[B19] Ashraf R. Sofi H. S. Akram T. Rather H. A. Abdal‐hay A. Shabir N. (2020). Fabrication of multifunctional cellulose/TiO _2_/Ag composite nanofibers scaffold with antibacterial and bioactivity properties for future tissue engineering applications. J. Biomed. Mater Res. A 108, 947–962. 10.1002/jbm.a.36872 31894888

[B20] Awais M. Aizaz A. Nazneen A. Bhatti Q. ul A. Akhtar M. Wadood A. (2022). A review on the recent advancements on therapeutic effects of ions in the physiological environments. Prosthesis 4, 263–316. 10.3390/prosthesis4020026

[B21] Badv M. Bayat F. Weitz J. I. Didar T. F. (2020). Single and multi-functional coating strategies for enhancing the biocompatibility and tissue integration of blood-contacting medical implants. Biomaterials 258, 120291. 10.1016/j.biomaterials.2020.120291 32798745

[B22] Bakhtiari H. Nouri A. Khakbiz M. Tolouei-Rad M. (2023). Fatigue behaviour of load-bearing polymeric bone scaffolds: a review. Acta Biomater. 172, 16–37. 10.1016/j.actbio.2023.09.048 37797705

[B23] Balcerak-Woźniak A. Dzwonkowska-Zarzycka M. Kabatc-Borcz J. (2024). A comprehensive review of stimuli-responsive smart polymer materials—recent advances and future perspectives. Materials 17, 4255. 10.3390/ma17174255 39274645 PMC11396725

[B24] Bandyopadhyay A. Mitra I. Goodman S. B. Kumar M. Bose S. (2023). Improving biocompatibility for next generation of metallic implants. Prog. Mater Sci. 133, 101053. 10.1016/j.pmatsci.2022.101053 36686623 PMC9851385

[B25] Bansal P. Katiyar D. Prakash S. Rao N. G. R. Saxena V. Kumar V. (2022). Applications of some biopolymeric materials as medical implants: an overview. Mater Today Proc. 65, 3377–3381. 10.1016/j.matpr.2022.05.480

[B26] Baranwal J. Barse B. Fais A. Delogu G. L. Kumar A. (2022). Biopolymer: a sustainable material for food and medical applications. Polym. (Basel) 14, 983. 10.3390/polym14050983 PMC8912672 35267803

[B27] Barclay T. G. Day C. M. Petrovsky N. Garg S. (2019). Review of polysaccharide particle-based functional drug delivery. Carbohydr. Polym. 221, 94–112. 10.1016/j.carbpol.2019.05.067 31227171 PMC6626612

[B28] Barroso T. Viveiros R. Casimiro T. Aguiar-Ricardo A. (2014). Development of dual-responsive chitosan–collagen scaffolds for pulsatile release of bioactive molecules. J. Supercrit. Fluids 94, 102–112. 10.1016/j.supflu.2014.07.005

[B29] Bazban-Shotorbani S. Hasani-Sadrabadi M. M. Karkhaneh A. Serpooshan V. Jacob K. I. Moshaverinia A. (2017). Revisiting structure-property relationship of pH-responsive polymers for drug delivery applications. J. Control. Release 253, 46–63. 10.1016/j.jconrel.2017.02.021 28242418

[B30] Bhatnagar P. Law J. X. Ng S.-F. (2022). Delivery systems for platelet derived growth factors in wound healing: a review of recent developments and global patent landscape. J. Drug Deliv. Sci. Technol. 71, 103270. 10.1016/j.jddst.2022.103270

[B31] Bhowmick A. Banerjee S. L. Pramanik N. Jana P. Mitra T. Gnanamani A. (2018a). Organically modified clay supported chitosan/hydroxyapatite-zinc oxide nanocomposites with enhanced mechanical and biological properties for the application in bone tissue engineering. Int. J. Biol. Macromol. 106, 11–19. 10.1016/j.ijbiomac.2017.07.168 28774805

[B32] Bhowmick A. Banerjee S. L. Pramanik N. Jana P. Mitra T. Gnanamani A. (2018b). Organically modified clay supported chitosan/hydroxyapatite-zinc oxide nanocomposites with enhanced mechanical and biological properties for the application in bone tissue engineering. Int. J. Biol. Macromol. 106, 11–19. 10.1016/j.ijbiomac.2017.07.168 28774805

[B33] Bibire T. Yilmaz O. Ghiciuc C. M. Bibire N. Dănilă R. (2022). Biopolymers for surgical applications. Coatings 12, 211. 10.3390/coatings12020211

[B34] Biswas M. C. Jony B. Nandy P. K. Chowdhury R. A. Halder S. Kumar D. (2022). Recent advancement of biopolymers and their potential biomedical applications. J. Polym. Environ. 30, 51–74. 10.1007/s10924-021-02199-y

[B35] Boia R. Dias P. A. N. Martins J. M. Galindo-Romero C. Aires I. D. Vidal-Sanz M. (2019). Porous poly(ε-caprolactone) implants: a novel strategy for efficient intraocular drug delivery. J. Control. Release 316, 331–348. 10.1016/j.jconrel.2019.09.023 31715277

[B36] Braem A. Kamarudin N. H. N. Bhaskar N. Hadzhieva Z. Mele A. Soulié J. (2023). Biomaterial strategies to combat implant infections: new perspectives to old challenges. Int. Mater. Rev. 68, 1011–1049. 10.1080/09506608.2023.2193784

[B37] Bratek-Skicki A. (2021). Towards a new class of stimuli-responsive polymer-based materials – recent advances and challenges. Appl. Surf. Sci. Adv. 4, 100068. 10.1016/j.apsadv.2021.100068

[B38] Buranapanitkit B. Srinilta V. Ingviga N. Oungbho K. Geater A. Ovatlarnporn C. (2004). The efficacy of a hydroxyapatite composite as a biodegradable antibiotic delivery system. Clin. Orthop. Relat. Res. 424, 244–252. 10.1097/01.blo.0000130268.27024.c1 15241172

[B39] Çağlar E. Ş. Yoltaş A. Özhan Y. Sipahi H. Aydın A. Üstündağ Okur N. (2024). Mucoadhesive electrospun nanofibrous poly(ε-caprolactone)/poly(lactic acid) matrices for the ocular delivery of moxifloxacin: a novel application of hyaluronic acid and xanthan gum blend as mucoadhesive coating agent. Int. J. Polym. Mater. Polym. Biomaterials, 1–16. 10.1080/00914037.2024.2335180

[B40] Campelo C. S. Chevallier P. Vaz J. M. Vieira R. S. Mantovani D. (2017). Sulfonated chitosan and dopamine based coatings for metallic implants in contact with blood. Mater. Sci. Eng. C 72, 682–691. 10.1016/j.msec.2016.11.133 28024638

[B41] Čanović S. Konjevoda S. Pavičić A. D. Stanić R. (2019). in Intraocular lens (IOL) materials. Editors Wang X. Ferreri F. M. Lens I. (Rijeka: IntechOpen). Ch. 2. 10.5772/intechopen.89985

[B42] Castellanos M. I. Zenses A.-S. Grau A. Rodríguez-Cabello J. C. Gil F. J. Manero J. M. (2015). Biofunctionalization of REDV elastin-like recombinamers improves endothelialization on CoCr alloy surfaces for cardiovascular applications. Colloids Surf. B Biointerfaces 127, 22–32. 10.1016/j.colsurfb.2014.12.056 25637794

[B43] Castilho M. Feyen D. Flandes‐Iparraguirre M. Hochleitner G. Groll J. Doevendans P. A. F. (2017). Melt electrospinning writing of poly‐hydroxymethylglycolide‐ *co* ‐ε‐Caprolactone‐Based scaffolds for cardiac tissue engineering. Adv. Healthc. Mater 6. 10.1002/adhm.201700311 PMC7116102 28699224

[B44] Catanio Bortolan C. Contri Campanelli L. Paternoster C. Giguère N. Brodusch N. Bolfarini C. (2021). Effect of oxygen content on the mechanical properties and plastic deformation mechanisms in the TWIP/TRIP Ti–12Mo alloy. Mater. Sci. Eng. A 817, 141346. 10.1016/j.msea.2021.141346

[B45] Catanio Bortolan C. Copes F. Shekargoftar M. de Oliveira Fidelis Sales V. Paternoster C. Contri Campanelli L. (2023). Electrochemical and *in vitro* biological behaviors of a Ti-Mo-Fe alloy specifically designed for stent applications. Biomaterials Biosyst. 10, 100076. 10.1016/j.bbiosy.2023.100076 PMC10240522 37284655

[B46] Catanio Bortolan C. Paternoster C. Turgeon S. Paoletti C. Cabibbo M. Lecis N. (2020). Plasma-immersion ion implantation surface oxidation on a cobalt-chromium alloy for biomedical applications. Biointerphases 15, 041004. 10.1116/6.0000278 32689805

[B47] Chacón J. M. Núñez P. J. Caminero M. A. García-Plaza E. Vallejo J. Blanco M. (2022). 3D printing of patient-specific 316L–stainless–steel medical implants using fused filament fabrication technology: two veterinary case studies. Biodes Manuf. 5, 808–815. 10.1007/s42242-022-00200-8

[B48] Chakrabarty G. Vashishtha M. Leeder D. (2015). Polyethylene in knee arthroplasty: a review. J. Clin. Orthop. Trauma 6, 108–112. 10.1016/j.jcot.2015.01.096 25983517 PMC4411358

[B49] Chen S. Chen Y. Zhao Y. Zhang L. Zhu C. Zhang Y. (2022). Status and strategies for fabricating flexible oxide ceramic micro-nanofiber materials. Mater. Today 61, 139–168. 10.1016/j.mattod.2022.11.004

[B50] Chen X. Chen G. Wang G. Zhu P. Gao C. (2020). Recent progress on 3D-printed polylactic acid and its applications in bone repair. Adv. Eng. Mater 22. 10.1002/adem.201901065

[B51] Cheng X. Wei Q. Ma Y. Shi R. Chen T. Wang Y. (2020). Antibacterial and osteoinductive biomacromolecules composite electrospun fiber. Int. J. Biol. Macromol. 143, 958–967. 10.1016/j.ijbiomac.2019.09.156 31739052

[B52] Concheiro A. Alvarez-Lorenzo C. (2013). Chemically cross-linked and grafted cyclodextrin hydrogels: from nanostructures to drug-eluting medical devices. Adv. Drug Deliv. Rev. 65, 1188–1203. 10.1016/j.addr.2013.04.015 23631979

[B53] Cooke M. E. Ramirez-GarciaLuna J. L. Rangel-Berridi K. Park H. Nazhat S. N. Weber M. H. (2020). 3D printed polyurethane scaffolds for the repair of bone defects. Front. Bioeng. Biotechnol. 8, 557215. 10.3389/fbioe.2020.557215 33195122 PMC7644785

[B54] Copes F. Fiocchi J. Gambaro S. Bregoli C. Tuissi A. Biffi C. A. (2024). Biological evaluation of femtosecond-laser-textured Fe–Mn alloys for vascular applications. Nanomater. Energy 13, 45–53. 10.1680/jnaen.23.00009

[B55] Costa B. C. Tokuhara C. K. Rocha L. A. Oliveira R. C. Lisboa-Filho P. N. Costa P. J. (2019). Vanadium ionic species from degradation of Ti-6Al-4V metallic implants: *in vitro* cytotoxicity and speciation evaluation. Mater. Sci. Eng. C 96, 730–739. 10.1016/j.msec.2018.11.090 30606586

[B56] Danckwerts M. Fassihi A. (1991). Implantable controlled release drug delivery systems: a review. Drug Dev. Ind. Pharm. 17, 1465–1502. 10.3109/03639049109026629

[B57] Das M. Solanki A. Joshi A. Devkar R. Seshadri S. Thakore S. (2019). β-cyclodextrin based dual-responsive multifunctional nanotheranostics for cancer cell targeting and dual drug delivery. Carbohydr. Polym. 206, 694–705. 10.1016/j.carbpol.2018.11.049 30553374

[B58] Dash A. Cudworth G. (1998). Therapeutic applications of implantable drug delivery systems. J. Pharmacol. Toxicol. Methods 40, 1–12. 10.1016/S1056-8719(98)00027-6 9920528

[B59] Davis R. Singh A. Jackson M. J. Coelho R. T. Prakash D. Charalambous C. P. (2022). A comprehensive review on metallic implant biomaterials and their subtractive manufacturing. Int. J. Adv. Manuf. Technol. 120, 1473–1530. 10.1007/s00170-022-08770-8 35228769 PMC8865884

[B60] de Andrade L. M. Paternoster C. Chevallier P. Gambaro S. Mengucci P. Mantovani D. (2022). Surface processing for iron-based degradable alloys: a preliminary study on the importance of acid pickling. Bioact. Mater 11, 166–180. 10.1016/j.bioactmat.2021.09.026 34938921 PMC8665346

[B61] Deng J. Song Q. Liu S. Pei W. Wang P. Zheng L. (2022). Advanced applications of cellulose-based composites in fighting bone diseases. Compos B Eng. 245, 110221. 10.1016/j.compositesb.2022.110221

[B62] Desante G. Pudełko I. Krok-Borkowicz M. Pamuła E. Jacobs P. Kazek-Kęsik A. (2023). Surface multifunctionalization of inert ceramic implants by calcium phosphate biomimetic coating doped with nanoparticles encapsulating antibiotics. ACS Appl. Mater Interfaces 15, 21699–21718. 10.1021/acsami.3c03884 37083334

[B63] de Souza Ferreira J. N. Vasconcelos V. V. V. Figueiredo B. S. Alves D. P. de Abreu ALLV de Souza P. P. (2023). “Chapter 10 - PLGA nanoparticles for treatment of cardiovascular diseases,” in Poly(lactic-co-glycolic acid) (PLGA) nanoparticles for drug delivery. Editor. Kesharwani P. (Elsevier), 267–302. 10.1016/B978-0-323-91215-0.00015-7

[B64] DeStefano V. Khan S. Tabada A. (2020). Applications of PLA in modern medicine. Eng. Regen. 1, 76–87. 10.1016/j.engreg.2020.08.002 38620328 PMC7474829

[B65] Diaz-Rodriguez S. Rasser C. Mesnier J. Chevallier P. Gallet R. Choqueux C. (2021a). Coronary stent CD31-mimetic coating favours endothelialization and reduces local inflammation and neointimal development *in vivo* . Eur. Heart J. 42, 1760–1769. 10.1093/eurheartj/ehab027 33580685 PMC8106951

[B66] Diaz-Rodriguez S. Rasser C. Mesnier J. Chevallier P. Gallet R. Choqueux C. (2021b). Coronary stent CD31-mimetic coating favours endothelialization and reduces local inflammation and neointimal development *in vivo* . Eur. Heart J. 42, 1760–1769. 10.1093/eurheartj/ehab027 33580685 PMC8106951

[B67] Di Martino A. Sittinger M. Risbud M. V. (2005). Chitosan: a versatile biopolymer for orthopaedic tissue-engineering. Biomaterials 26, 5983–5990. 10.1016/j.biomaterials.2005.03.016 15894370

[B68] Ding S.-J. Chu Y.-H. Chen P.-T. (2021). Mechanical biocompatibility, osteogenic activity, and antibacterial efficacy of calcium silicate–zirconia biocomposites. ACS Omega 6, 7106–7118. 10.1021/acsomega.1c00097 33748624 PMC7970563

[B69] Do M. P. Neut C. Metz H. Delcourt E. Mäder K. Siepmann J. (2015). *In-situ* forming composite implants for periodontitis treatment: how the formulation determines system performance. Int. J. Pharm. 486, 38–51. 10.1016/j.ijpharm.2015.03.026 25791762

[B70] Dong H. Lin F. Boccaccini A. R. Virtanen S. (2021). Corrosion behavior of biodegradable metals in two different simulated physiological solutions: comparison of Mg, Zn and Fe. Corros. Sci. 182, 109278. 10.1016/j.corsci.2021.109278

[B71] Dong H. Liu H. Zhou N. Li Q. Yang G. Chen L. (2020). Surface modified techniques and emerging functional coating of dental implants. Coatings 10, 1012. 10.3390/coatings10111012

[B72] Dornelas J. Dornelas G. Rossi A. Piattelli A. Di Pietro N. Romasco T. (2024). The incorporation of zinc into hydroxyapatite and its influence on the cellular response to biomaterials: a systematic review. J. Funct. Biomater. 15, 178. 10.3390/jfb15070178 39057300 PMC11277605

[B73] Du J.-K. Chao C.-Y. Jhong Y.-T. Wu C.-H. Wu J.-H. (2016). Effects of Cr2N precipitation on the antibacterial properties of AISI 430 stainless steel. Met. (Basel) 6, 73. 10.3390/met6040073

[B74] Eftekhar Ashtiani R. Alam M. Tavakolizadeh S. Abbasi K. (2021). The role of biomaterials and biocompatible materials in implant-supported dental prosthesis. Evidence-Based Complementary Altern. Med. 2021, 1–9. 10.1155/2021/3349433 PMC8360736 34394378

[B75] Eğri S. Eczacıoğlu N. (2017). Sequential VEGF and BMP-2 releasing PLA-PEG-PLA scaffolds for bone tissue engineering: I. Design and *in vitro* tests. Artif. Cells Nanomed Biotechnol. 45, 321–329. 10.3109/21691401.2016.1147454 26912262

[B76] Elyada A. Garti N. Füredi-Milhofer H. (2014). Polyelectrolyte multilayer-calcium phosphate composite coatings for metal implants. Biomacromolecules 15, 3511–3521. 10.1021/bm5006245 25105729

[B77] Ershad-Langroudi A. Babazadeh N. Alizadegan F. Mehdi Mousaei S. Moradi G. Polymers for implantable devices (2024a). J. Industrial Eng. Chem. 137, 61–86. 10.1016/j.jiec.2024.03.030

[B78] Ershad-Langroudi A. Babazadeh N. Alizadegan F. Mehdi Mousaei S. Moradi G. (2024b). Polymers for implantable devices. J. Industrial Eng. Chem. 137, 61–86. 10.1016/j.jiec.2024.03.030

[B79] Escobar K. Carrera I. Naveas N. Pulido R. Manso M. Guarnieri J. P. de O. (2023b). Functionalization of breast implants by cyclodextrin *in-situ* polymerization: a local drug delivery system for augmentation mammaplasty. Front. Bioeng. Biotechnol. 11, 1254299. 10.3389/fbioe.2023.1254299 37811378 PMC10557261

[B80] Escobar K. Garrido-Miranda K. A. Pulido R. Naveas N. Manso-Silván M. Hernandez-Montelongo J. (2023a). Coatings of cyclodextrin/citric-acid biopolymer as drug delivery systems: a review. Pharmaceutics 15, 296. 10.3390/pharmaceutics15010296 36678924 PMC9865107

[B81] Esfahani G. Häusler O. Mäder K. (2022). Controlled release starch-lipid implant for the therapy of severe malaria. Int. J. Pharm. 622, 121879. 10.1016/j.ijpharm.2022.121879 35649475

[B82] Esfahani G. Lucas H. Syrowatka F. Mäder K. (2023a). A starch-based implant as a controlled drug release system: non-invasive *in vivo* characterization using multispectral fluorescence imaging. J. Control. Release 358, 358–367. 10.1016/j.jconrel.2023.05.006 37156301

[B83] Esfahani G. Trutschel M.-L. Reichert D. Mäder K. (2023b). Characterization of controlled release starch-nimodipine implant for antispasmodic and neuroprotective therapies in the brain. Mol. Pharm. 20, 5753–5762. 10.1021/acs.molpharmaceut.3c00618 37750866

[B84] Eslahi N. Mahmoodi A. Mahmoudi N. Zandi N. Simchi A. (2020). Processing and properties of nanofibrous bacterial cellulose-containing polymer composites: a review of recent advances for biomedical applications. Polym. Rev. 60, 144–170. 10.1080/15583724.2019.1663210

[B85] Faccio E. Aquili L. Rocelli M. (2022). What is therapeutic? Analysis of the narratives available on Italian Addiction Rehab Centres’ Sites to present the therapeutic programme. Res. Psychotherapy Psychopathol. Process Outcome 26, 613. 10.4081/ripppo.2022.613 PMC10204598 36052880

[B86] Fan J. He Q. Liu Y. Zhang F. Yang X. Wang Z. (2016). Light-responsive biodegradable nanomedicine overcomes multidrug resistance via NO-enhanced chemosensitization. ACS Appl. Mater Interfaces 8, 13804–13811. 10.1021/acsami.6b03737 27213922 PMC5233726

[B87] Faria J. Dionísio B. Soares Í. Baptista A. C. Marques A. Gonçalves L. (2022). Cellulose acetate fibres loaded with daptomycin for metal implant coatings. Carbohydr. Polym. 276, 118733. 10.1016/j.carbpol.2021.118733 34823769

[B88] Fathi-Karkan S. Banimohamad-Shotorbani B. Saghati S. Rahbarghazi R. Davaran S. (2022). A critical review of fibrous polyurethane-based vascular tissue engineering scaffolds. J. Biol. Eng. 16, 6. 10.1186/s13036-022-00286-9 35331305 PMC8951709

[B89] Feerick E. M. Kennedy J. Mullett H. FitzPatrick D. McGarry P. (2013). Investigation of metallic and carbon fibre PEEK fracture fixation devices for three-part proximal humeral fractures. Med. Eng. Phys. 35, 712–722. 10.1016/j.medengphy.2012.07.016 22989528

[B90] Feng P. Jia J. Liu M. Peng S. Zhao Z. Shuai C. (2021). Degradation mechanisms and acceleration strategies of poly (lactic acid) scaffold for bone regeneration. Mater Des. 210, 110066. 10.1016/j.matdes.2021.110066

[B91] Feng P. Wei P. Shuai C. Peng S. (2014). Characterization of mechanical and biological properties of 3-D scaffolds reinforced with zinc oxide for bone tissue engineering. PLoS One 9, e87755. 10.1371/journal.pone.0087755 24498185 PMC3909231

[B92] Ferraz M. P. (2022). Biomaterials for ophthalmic applications. Appl. Sci. Switz. 12, 5886. 10.3390/app12125886

[B93] Ferretto I. Kim D. Della Ventura N. M. Shahverdi M. Lee W. Leinenbach C. (2021). Laser powder bed fusion of a Fe–Mn–Si shape memory alloy. Addit. Manuf. 46, 102071. 10.1016/j.addma.2021.102071

[B94] Ferroni L. D’Amora U. Leo S. Tremoli E. Raucci M. G. Ronca A. (2022). PEEK and hyaluronan-based 3D printed structures: promising combination to improve bone regeneration. Molecules 27, 8749. 10.3390/molecules27248749 36557882 PMC9787780

[B95] Filip N. Radu I. Veliceasa B. Filip C. Pertea M. Clim A. (2022). Biomaterials in orthopedic devices: current issues and future perspectives. Coatings 12, 1544. 10.3390/coatings12101544

[B96] Filipov E. Yildiz R. Dikovska A. Sotelo L. Soma T. Avdeev G. (2024). Design of laser activated antimicrobial porous tricalcium phosphate-hydroxyapatite scaffolds for orthopedic applications. J. Funct. Biomater. 15, 36. 10.3390/jfb15020036 38391889 PMC10889241

[B97] Fishbein I. Chorny M. Alferiev I. S. Levy R. J. (2012) “Site specific controlled release for cardiovascular disease: translational directions,” in Fundamentals and applications of controlled release drug delivery (Boston, MA: Springer US), 445–492. 10.1007/978-1-4614-0881-9_14

[B98] Flaig F. Ragot H. Simon A. Revet G. Kitsara M. Kitasato L. (2020). Design of functional electrospun scaffolds based on poly(glycerol sebacate) elastomer and poly(lactic acid) for cardiac tissue engineering. ACS Biomater. Sci. Eng. 6, 2388–2400. 10.1021/acsbiomaterials.0c00243 33455317

[B99] Food and Drug Administration (FDA) (2018). USP therapeutic categories model guidelines. Available at: https://www.fda.gov/regulatory-information/fdaaa-implementation-chart/usp-therapeutic-categories-model-guidelines (Accessed July 19, 2024).

[B100] Fratila A. Jimenez-Marcos C. Mirza-Rosca J. C. Saceleanu A. (2023). Mechanical properties and biocompatibility of various cobalt chromium dental alloys. Mater Chem. Phys. 304, 127867. 10.1016/j.matchemphys.2023.127867

[B101] Fredi G. Dorigato A. (2024). Compatibilization of biopolymer blends: a review. Adv. Industrial Eng. Polym. Res. 7, 373–404. 10.1016/j.aiepr.2023.11.002

[B102] Fu C. Yang X. Tan S. Song L. (2017). Enhancing cell proliferation and osteogenic differentiation of mc3t3-E1 pre-osteoblasts by BMP-2 delivery in graphene oxide-incorporated PLGA/HA biodegradable microcarriers. Sci. Rep. 7, 12549. 10.1038/s41598-017-12935-x 28970533 PMC5624967

[B103] Fujihara K. Teo K. Gopal R. Loh P. L. Ganesh V. K. Ramakrishna S. (2004a). Fibrous composite materials in dentistry and orthopaedics: review and applications. Compos Sci. Technol. 64, 775–788. 10.1016/j.compscitech.2003.09.012

[B104] Fujihara K. Teo K. Gopal R. Loh P. L. Ganesh V. K. Ramakrishna S. (2004b). Fibrous composite materials in dentistry and orthopaedics: review and applications. Compos Sci. Technol. 64, 775–788. 10.1016/j.compscitech.2003.09.012

[B105] Gaharwar A. K. Singh I. Khademhosseini A. (2020). Engineered biomaterials for *in situ* tissue regeneration. Nat. Rev. Mater 5, 686–705. 10.1038/s41578-020-0209-x

[B106] Gaidai O. Cao Y. Loginov S. (2023). Global cardiovascular diseases death rate prediction. Curr. Probl. Cardiol. 48, 101622. 10.1016/j.cpcardiol.2023.101622 36724816

[B107] Gambaro S. Paternoster C. Occhionero B. Fiocchi J. Biffi C. A. Tuissi A. (2021). Mechanical and degradation behavior of three Fe-Mn-C alloys for potential biomedical applications. Mater Today Commun. 27, 102250. 10.1016/j.mtcomm.2021.102250

[B108] García-Estrada P. García-Bon M. A. López-Naranjo E. J. Basaldúa-Pérez D. N. Santos A. Navarro-Partida J. (2021). Polymeric implants for the treatment of intraocular eye diseases: trends in biodegradable and non-biodegradable materials. Pharmaceutics 13, 701. 10.3390/pharmaceutics13050701 34065798 PMC8151640

[B109] García-Rey E. García-Cimbrelo E. (2010). Polyethylene in total hip arthroplasty: half a century in the limelight. J. Orthop. Traumatology 11, 67–72. 10.1007/s10195-010-0091-1 PMC2896572 20505976

[B110] Ghasemi-Mobarakeh L. Kolahreez D. Ramakrishna S. Williams D. (2019). Key terminology in biomaterials and biocompatibility. Curr. Opin. Biomed. Eng. 10, 45–50. 10.1016/j.cobme.2019.02.004

[B111] Glenske K. Donkiewicz P. Köwitsch A. Milosevic-Oljaca N. Rider P. Rofall S. (2018). Applications of metals for bone regeneration. Int. J. Mol. Sci. 19, 826. 10.3390/ijms19030826 29534546 PMC5877687

[B112] Grosgogeat B. Vaicelyte A. Gauthier R. Janssen C. Le Borgne M. (2022). Toxicological risks of the cobalt–chromium alloys in dentistry: a systematic review. Materials 15, 5801. 10.3390/ma15175801 36079183 PMC9457507

[B113] Guo L. Liang Z. Yang L. Du W. Yu T. Tang H. (2021). The role of natural polymers in bone tissue engineering. J. Control. Release 338, 571–582. 10.1016/j.jconrel.2021.08.055 34481026

[B114] Gupta P. Mandal B. B. (2021). Tissue-engineered vascular grafts: emerging trends and technologies. Adv. Funct. Mater 31. 10.1002/adfm.202100027

[B115] Hallab N. J. Jacobs J. J. (2020). 2.5.4 - orthopedic applications,” in Biomaterials science. Fourth Edition, Editors. Wagner W. R. Sakiyama-Elbert S. E. Zhang G. Yaszemski M. J. (Academic Press), 1079–1118. 10.1016/B978-0-12-816137-1.00070-2

[B116] Hao W. Zheng Z. Zhu L. Pang L. Ma J. Zhu S. (2021). 3D printing‐based drug-loaded implanted prosthesis to prevent breast cancer recurrence post‐conserving surgery. Asian J. Pharm. Sci. 16, 86–96. 10.1016/j.ajps.2020.06.002 33613732 PMC7878459

[B117] Harman M. K. (2020). “Medical device failure—implant retrieval, evaluation, and failure analysis,” in Biomaterials science (Elsevier), 1485–1495. 10.1016/B978-0-12-816137-1.00096-9

[B118] Hashimoto Y. Yamashita A. Negishi J. Kimura T. Funamoto S. Kishida A. (2022). 4-Arm PEG-functionalized decellularized pericardium for effective prevention of postoperative adhesion in cardiac surgery. ACS Biomater. Sci. Eng. 8, 261–272. 10.1021/acsbiomaterials.1c00990 34937336

[B119] He Y. Jin Y. Ying X. Wu Q. Yao S. Li Y. (2020). Development of an antimicrobial peptide-loaded mineralized collagen bone scaffold for infective bone defect repair. Regen. Biomater. 7, 515–525. 10.1093/rb/rbaa015 33149940 PMC7597801

[B120] He Y. Wang Q. Liu Y. Zhang Z. Cao Z. Wang S. (2024a). Composite mineralized collagen/polycaprolactone scaffold-loaded microsphere system with dual osteogenesis and antibacterial functions. Polym. (Basel) 16, 2394. 10.3390/polym16172394 PMC11397082 39274026

[B121] He Y. Wang Q. Liu Y. Zhang Z. Cao Z. Wang S. (2024b). Composite mineralized collagen/polycaprolactone scaffold-loaded microsphere system with dual osteogenesis and antibacterial functions. Polym. (Basel) 16, 2394. 10.3390/polym16172394 PMC11397082 39274026

[B122] Heidari B. S. Oliaei E. Shayesteh H. Davachi S. M. Hejazi I. Seyfi J. (2017). Simulation of mechanical behavior and optimization of simulated injection molding process for PLA based antibacterial composite and nanocomposite bone screws using central composite design. J. Mech. Behav. Biomed. Mater 65, 160–176. 10.1016/j.jmbbm.2016.08.008 27572233

[B123] Heidari F. Bazargan‐Lari R. Razavi M. Fahimipour F. Vashaee D. Tayebi L. (2020a). Nano‐hydroxyapatite and nano‐hydroxyapatite/zinc oxide scaffold for bone tissue engineering application. Int. J. Appl. Ceram. Technol. 17, 2752–2761. 10.1111/ijac.13596

[B124] Heidari F. Tabatabaei F. S. Razavi M. Lari R. B. Tavangar M. Romanos G. E. (2020b). 3D construct of hydroxyapatite/zinc oxide/palladium nanocomposite scaffold for bone tissue engineering. J. Mater Sci. Mater Med. 31, 85. 10.1007/s10856-020-06409-2 33000320

[B125] Hernández-Montes V. Buitrago-Sierra R. Echeverry-Rendón M. Santa-Marín J. F. (2023). Ceria-based coatings on magnesium alloys for biomedical applications: a literature review. RSC Adv. 13, 1422–1433. 10.1039/D2RA06312C 36712919 PMC9829028

[B126] Heydari S. Asefnejad A. Hassanzadeh Nemati N. Goodarzi V. Vaziri A. (2021). Fabrication of nanocomposite scaffold based on bacterial cellulose/zinc oxide nanoparticles/polypyrrole with antibacterial and cytotoxicity assessment. J. Appl. Res. Chem. -Polymer Eng. 5, 37–50.

[B127] Hosseinalipour M. Javadpour J. Rezaie H. Dadras T. Hayati A. N. (2010). Investigation of mechanical properties of experimental bis-GMA/TEGDMA dental composite resins containing various mass fractions of silica nanoparticles. J. Prosthodont. 19, 112–117. 10.1111/j.1532-849X.2009.00530.x 19895426

[B128] Hu T. Yang C. Lin S. Yu Q. Wang G. (2018). Biodegradable stents for coronary artery disease treatment: recent advances and future perspectives. Mater. Sci. Eng. C 91, 163–178. 10.1016/j.msec.2018.04.100 30033243

[B129] Hu Y. Li S. Dong H. Weng L. Yuwen L. Xie Y. (2023a). Environment‐responsive therapeutic platforms for the treatment of implant infection. Adv. Healthc. Mater 12, e2300985. 10.1002/adhm.202300985 37186891

[B130] Hu Y. Li S. Dong H. Weng L. Yuwen L. Xie Y. (2023b). Environment‐responsive therapeutic platforms for the treatment of implant infection. Adv. Healthc. Mater 12, e2300985. 10.1002/adhm.202300985 37186891

[B131] Hua W. Shi W. Mitchell K. Raymond L. Coulter R. Zhao D. 3D printing of biodegradable polymer vascular stents: a review (2022). Chin. J. Mech. Eng. Addit. Manuf. Front. 1, 100020. 10.1016/j.cjmeam.2022.100020

[B132] Huang S. Ulloa A. Nauman E. Stanciu L. (2020). Collagen coating effects on Fe–Mn bioresorbable alloys. J. Orthop. Res. 38, 523–535. 10.1002/jor.24492 31608487

[B133] Huo Y. Lyu Y. Bosiakov S. Han F. (2022). A critical review of the design, manufacture, and evaluation of bone joint replacements for bone repair. Materials 15, 153. 10.3390/ma15010153 PMC8746215 35009299

[B134] Ibrahim R. M. Kamoun E. A. Badawi N. M. EL-Moslamy S. H. kh M. Salim S. A. (2024). Cutting-edge biomaterials for advanced biomedical uses: self-gelation of l -arginine-loaded chitosan/PVA/vanillin hydrogel for accelerating topical wound healing and skin regeneration. RSC Adv. 14, 31126–31142. 10.1039/D4RA04430D 39351417 PMC11441373

[B135] Iimaa T. Hirayama T. Shirakigawa N. Imai D. Yamao T. Yamashita Y. (2019). Antibacterial-agent-immobilized gelatin hydrogel as a 3D scaffold for natural and bioengineered tissues. Gels 5, 32. 10.3390/gels5020032 31212711 PMC6630779

[B136] Implants and Prosthetics (2019). Food and Drug Administration(FDA). Available at: https://www.fda.gov/medical-devices/products-and-medical-procedures/implants-and-prosthetics (Accessed July 24, 2024).

[B137] Improvement of corrosion resistance of 316L stainless steel substrate with a composite coating of biopolymer produced by electrophoretic deposition (2023). Int. J. Corros. Scale Inhibition 12. 10.17675/2305-6894-2023-12-3-7

[B138] Inoue B. S. Streit S. dos Santos Schneider A. L. Meier M. M. (2020). Bioactive bacterial cellulose membrane with prolonged release of chlorhexidine for dental medical application. Int. J. Biol. Macromol. 148, 1098–1108. 10.1016/j.ijbiomac.2020.01.036 31917984

[B139] Islam M. M. Shahruzzaman Md Biswas S. Nurus Sakib Md Rashid T. U. (2020). Chitosan based bioactive materials in tissue engineering applications-A review. Bioact. Mater 5, 164–183. 10.1016/j.bioactmat.2020.01.012 32083230 PMC7016353

[B140] Jagur-Grodzinski J. (2006). Polymers for tissue engineering, medical devices, and regenerative medicine. Concise general review of recent studies. Polym. Adv. Technol. 17, 395–418. 10.1002/pat.729

[B141] Jana P. Shyam M. Singh S. Jayaprakash V. Dev A. (2021). Biodegradable polymers in drug delivery and oral vaccination. Eur. Polym. J. 142, 110155. 10.1016/j.eurpolymj.2020.110155

[B142] Janmohammadi M. Nazemi Z. Salehi A. O. M. Seyfoori A. John J. V. Nourbakhsh M. S. (2023). Cellulose-based composite scaffolds for bone tissue engineering and localized drug delivery. Bioact. Mater 20, 137–163. 10.1016/j.bioactmat.2022.05.018 35663339 PMC9142858

[B143] Jayawardena H. S. N. Liyanage S. H. Rathnayake K. Patel U. Yan M. (2021). Analytical methods for characterization of nanomaterial surfaces. Anal. Chem. 93, 1889–1911. 10.1021/acs.analchem.0c05208 33434434 PMC7941215

[B144] Jensen L. K. Jensen H. E. Blirup-Plum S. A. Bue M. Hanberg P. Kvich L. (2022). Coating of bone implants with silica, hyperbranched polyethyleneimine, and gentamicin prevents development of osteomyelitis in a porcine model. Mater. (Oxf) 24, 101473. 10.1016/j.mtla.2022.101473

[B145] Jindal S. Manzoor F. Haslam N. Mancuso E. (2021). 3D printed composite materials for craniofacial implants: current concepts, challenges and future directions. Int. J. Adv. Manuf. Technol. 112, 635–653. 10.1007/s00170-020-06397-1

[B146] Johnbosco C. Zschoche S. Nitschke M. Hahn D. Werner C. Maitz M. F. (2021). Bioresponsive starPEG-heparin hydrogel coatings on vascular stents for enhanced hemocompatibility. Mater. Sci. Eng. C 128, 112268. 10.1016/j.msec.2021.112268 34474827

[B147] Jurczak P. Witkowska J. Rodziewicz-Motowidło S. Lach S. (2020). Proteins, peptides and peptidomimetics as active agents in implant surface functionalization. Adv. Colloid Interface Sci. 276, 102083. 10.1016/j.cis.2019.102083 31887572

[B148] Kaetsu I. Yoshida M. Asano M. Yamanaka H. Imai K. Yuasa H. (1987). Biodegradable implant composites for local therapy. J. Control. Release 6, 249–263. 10.1016/0168-3659(87)90080-0

[B149] Kang X. Zhang W. Yang C. (2016). Mechanical properties study of micro- and nano-hydroxyapatite reinforced ultrahigh molecular weight polyethylene composites. J. Appl. Polym. Sci. 133. 10.1002/app.42869

[B150] Kantak M. N. Bharate S. S. (2022). Analysis of clinical trials on biomaterial and therapeutic applications of chitosan: a review. Carbohydr. Polym. 278, 118999. 10.1016/j.carbpol.2021.118999 34973801

[B151] Karaaslan Tunç M. G. Karadaş Gedik K. Yumuşak A. İ. Yılmaz İ. Ateş B. (2024). “Chapter 10 - polymers for IOL systems,” in Polymeric materials for biomedical implants, Editors S. Thomas A. Tharayil (London, United Kingdom: Woodhead Publishing). 253–270. 10.1016/B978-0-323-99690-7.00017-0

[B152] Karacan I. Ben-Nissan B. Wang H. A. Juritza A. Swain M. V. Müller W. H. (2019). Mechanical testing of antimicrobial biocomposite coating on metallic medical implants as drug delivery system. Mater. Sci. Eng. C 104, 109757. 10.1016/j.msec.2019.109757 31499987

[B153] Karayilan M. Clamen L. Becker M. L. (2021). Polymeric materials for eye surface and intraocular applications. Biomacromolecules 22, 223–261. 10.1021/acs.biomac.0c01525 33405900

[B154] Kazimierczak P. Przekora A. (2020). Osteoconductive and osteoinductive surface modifications of biomaterials for bone regeneration: a concise review. Coatings 10, 971. 10.3390/coatings10100971

[B155] Kharkar P. B. Talkar S. S. Kadwadkar N. A. Patravale V. B. (2017). “Nanosystems for oral delivery of immunomodulators,” in Nanostructures for oral medicine (Elsevier), 295–334. 10.1016/B978-0-323-47720-8.00012-2

[B156] Khatun H. Rahman M. Mahmud S. Rahaman M. Ali O. Ali Y. (2024). Synthesis and characterization of biocompatible hybrid coating on WE54 Mg alloy for implant applications. Results Eng. 21, 101784. 10.1016/j.rineng.2024.101784

[B157] Khelifa F. Ershov S. Habibi Y. Snyders R. Dubois P. (2016). Free-radical-induced grafting from plasma polymer surfaces. Chem. Rev. 116, 3975–4005. 10.1021/acs.chemrev.5b00634 26943005

[B158] Khoshaim A. B. Moustafa E. B. Youness R. A. (2024). Antibacterial, mechanical, and dielectric properties of hydroxyapatite cordierite/zirconia porous nanocomposites for use in bone tissue engineering applications. Nanotechnol. Rev. 13. 10.1515/ntrev-2023-0175

[B159] Khouri N. G. Bahú J. O. Blanco-Llamero C. Severino P. Concha V. O. C. Souto E. B. (2024). Polylactic acid (PLA): properties, synthesis, and biomedical applications – a review of the literature. J. Mol. Struct. 1309, 138243. 10.1016/j.molstruc.2024.138243

[B160] Kim T. G. Shin H. Lim D. W. (2012). Biomimetic scaffolds for tissue engineering. Adv. Funct. Mater 22, 2446–2468. 10.1002/adfm.201103083

[B161] Kim Y.-K. Kim S.-Y. Jang Y.-S. Park I.-S. Lee M.-H. (2020). Bio-corrosion behaviors of hyaluronic acid and cerium multi-layer films on degradable implant. Appl. Surf. Sci. 515, 146070. 10.1016/j.apsusc.2020.146070

[B162] Knight M. Curliss D. (2003) “Composite materials,” in Encyclopedia of physical science and technology. Elsevier, 455–468. 10.1016/B0-12-227410-5/00128-9

[B163] Kovochich M. Monnot A. Kougias D. G. More S. L. Wilsey J. T. Qiu Q.-Q. (2021). Carcinogenic hazard assessment of cobalt-containing alloys in medical devices: review of *in vivo* studies. Regul. Toxicol. Pharmacol. 122, 104910. 10.1016/j.yrtph.2021.104910 33662479

[B164] Kravanja K. A. Finšgar M. (2021). Analytical techniques for the characterization of bioactive coatings for orthopaedic implants. Biomedicines 9, 1936. 10.3390/biomedicines9121936 34944750 PMC8698289

[B165] Krishnan V. Lakshmi T. (2013). Bioglass: a novel biocompatible innovation. J. Adv. Pharm. Technol. Res. 4, 78. 10.4103/2231-4040.111523 23833747 PMC3696226

[B166] Kumari A. Yadav S. K. Yadav S. C. (2010). Biodegradable polymeric nanoparticles based drug delivery systems. Colloids Surf. B Biointerfaces 75, 1–18. 10.1016/j.colsurfb.2009.09.001 19782542

[B167] Kurtz S. M. Devine J. N. (2007). PEEK biomaterials in trauma, orthopedic, and spinal implants. Biomaterials 28, 4845–4869. 10.1016/j.biomaterials.2007.07.013 17686513 PMC2040108

[B168] Lai J.-Y. (2012). Biocompatibility of genipin and glutaraldehyde cross-linked chitosan materials in the anterior chamber of the eye. Int. J. Mol. Sci. 13, 10970–10985. 10.3390/ijms130910970 23109832 PMC3472724

[B169] Lawton K. Le H. Tredwin C. Handy R. D. (2019). Carbon nanotube reinforced hydroxyapatite nanocomposites as bone implants: nanostructure, mechanical strength and biocompatibility. Int. J. Nanomedicine 14, 7947–7962. 10.2147/IJN.S218248 31632010 PMC6779593

[B170] Lee C. (2022). Development of injectable and biodegradable needle-type starch implant for effective intratumoral drug delivery and distribution. Int. J. Nanomedicine 17, 4307–4319. 10.2147/IJN.S370194 36147547 PMC9488191

[B171] Lee I. Luo X. Huang J. Cui X. T. Yun M. (2012). Detection of cardiac biomarkers using single polyaniline nanowire-based conductometric biosensors. Biosens. (Basel) 2, 205–220. 10.3390/bios2020205 PMC4263570 25585711

[B172] Lee J. E. Kim K. E. Kwon I. C. Ahn H. J. Lee S.-H. Cho H. (2004). Effects of the controlled-released TGF-β1 from chitosan microspheres on chondrocytes cultured in a collagen/chitosan/glycosaminoglycan scaffold. Biomaterials 25, 4163–4173. 10.1016/j.biomaterials.2003.10.057 15046906

[B173] Lee K. Y. Mooney D. J. (2012). Alginate: properties and biomedical applications. Prog. Polym. Sci. 37, 106–126. 10.1016/j.progpolymsci.2011.06.003 22125349 PMC3223967

[B174] Lee W. H. Cha G. D. Kim D.-H. (2021). Flexible and biodegradable electronic implants for diagnosis and treatment of brain diseases. Curr. Opin. Biotechnol. 72, 13–21. 10.1016/j.copbio.2021.07.027 34425329

[B175] Li J. Stachowski M. Zhang Z. (2015a) “Application of responsive polymers in implantable medical devices and biosensors,” in Switchable and responsive surfaces and materials for biomedical applications (Elsevier), 259–298. 10.1016/B978-0-85709-713-2.00011-0

[B176] Li J. Stachowski M. Zhang Z. (2015b). “11 - application of responsive polymers in implantable medical devices and biosensors,” in Switchable and responsive surfaces and materials for biomedical applications, Editor. Z. Zhang (Oxford: Woodhead Publishing), 259–298. 10.1016/B978-0-85709-713-2.00011-0

[B177] Li M. Jiang M. Gao Y. Zheng Y. Liu Z. Zhou C. (2022). Current status and outlook of biodegradable metals in neuroscience and their potential applications as cerebral vascular stent materials. Bioact. Mater 11, 140–153. 10.1016/j.bioactmat.2021.09.025 34938919 PMC8665265

[B178] Li S. Hu K. Cao W. Sun Y. Sheng W. Li F. (2014). PH-responsive biocompatible fluorescent polymer nanoparticles based on phenylboronic acid for intracellular imaging and drug delivery. Nanoscale 6, 13701–13709. 10.1039/c4nr04054f 25278283

[B179] Li Y. Qiao Z. Yu F. Hu H. Huang Y. Xiang Q. (2019b). Transforming growth factor-β3/chitosan sponge (TGF-β3/CS) facilitates osteogenic differentiation of human periodontal ligament stem cells. Int. J. Mol. Sci. 20, 4982. 10.3390/ijms20204982 31600954 PMC6834328

[B180] Li Y. Wang D. Qin W. Jia H. Wu Y. Ma J. (2019a). Mechanical properties, hemocompatibility, cytotoxicity and systemic toxicity of carbon fibers/poly(ether-ether-ketone) composites with different fiber lengths as orthopedic implants. J. Biomater. Sci. Polym. Ed. 30, 1709–1724. 10.1080/09205063.2019.1659711 31464157

[B181] Liu S. Qin S. He M. Zhou D. Qin Q. Wang H. (2020b). Current applications of poly(lactic acid) composites in tissue engineering and drug delivery. Compos B Eng. 199, 108238. 10.1016/j.compositesb.2020.108238

[B182] Liu S. Zhu Y. Gao H. Ge P. Ren K. Gao J. (2018). One-step fabrication of functionalized poly(etheretherketone) surfaces with enhanced biocompatibility and osteogenic activity. Mater. Sci. Eng. C 88, 70–78. 10.1016/j.msec.2018.03.003 29636140

[B183] Liu X. Zhou C. Xie Q. Xia L. Liu L. Bao W. (2024). Recent advances in layer-by-layer assembly scaffolds for co-delivery of bioactive molecules for bone regeneration: an updated review. J. Transl. Med. 22, 1001. 10.1186/s12967-024-05809-0 39501263 PMC11539823

[B184] Liu Y. Rath B. Tingart M. Eschweiler J. (2020c). Role of implants surface modification in osseointegration: a systematic review. J. Biomed. Mater Res. A 108, 470–484. 10.1002/jbm.a.36829 31664764

[B185] Liu Y. Wu G. de Groot K. (2010). Biomimetic coatings for bone tissue engineering of critical-sized defects. J. R. Soc. Interface 7, S631–S647. 10.1098/rsif.2010.0115.focus 20484228 PMC2952178

[B186] Liu Y. Yin P. Chen J. Cui B. Zhang C. Wu F. (2020a). Conducting polymer-based composite materials for therapeutic implantations: from advanced drug delivery system to minimally invasive electronics. Int. J. Polym. Sci. 2020, 1–16. 10.1155/2020/5659682

[B187] Liu C. Matsunami C. Shirosaki Y. Miyazaki T. (2015). Bioactive Co-Cr alloy for biomedical applications prepared by surface modification using self-assembled monolayers and poly-γ-glutamic acid. Dent. Mater J. 34, 707–712. 10.4012/dmj.2015-064 26438996

[B188] Lloyd A. W. Faragher R. G. A. Denyer S. P. (2001). Ocular biomaterials and implants. Biomaterials 22, 769–785. 10.1016/S0142-9612(00)00237-4 11246945

[B189] Loffredo S. Gambaro S. Copes F. Paternoster C. Giguère N. Vedani M. (2022). Effect of silver in thermal treatments of Fe-Mn-C degradable metals: implications for stent processing. Bioact. Mater 12, 30–41. 10.1016/j.bioactmat.2021.10.020 35087961 PMC8777259

[B190] Lopes J. H. Tabary N. Hernandez-Montelongo J. (2022). Editorial: biomacromolecules systems applied to medical implants for the release of therapeutic agents. Front. Bioeng. Biotechnol. 10, 910203. 10.3389/fbioe.2022.910203 35573241 PMC9091649

[B191] López-Valverde N. Aragoneses J. López-Valverde A. Rodríguez C. Macedo de Sousa B. Aragoneses J. M. (2022a). Role of chitosan in titanium coatings. trends and new generations of coatings. Front. Bioeng. Biotechnol. 10, 907589. 10.3389/fbioe.2022.907589 35935477 PMC9354072

[B192] López-Valverde N. Aragoneses J. López-Valverde A. Rodríguez C. Macedo de Sousa B. Aragoneses J. M. (2022b). Role of chitosan in titanium coatings. trends and new generations of coatings. Front. Bioeng. Biotechnol. 10, 907589. 10.3389/fbioe.2022.907589 35935477 PMC9354072

[B193] Low Y. J. Andriyana A. Ang B. C. Zainal Abidin N. I. (2020). Bioresorbable and degradable behaviors of PGA: current state and future prospects. Polym. Eng. Sci. 60, 2657–2675. 10.1002/pen.25508

[B194] Lü J.-M. Wang X. Marin-Muller C. Wang H. Lin P. H. Yao Q. (2009). Current advances in research and clinical applications of PLGA-based nanotechnology. Expert Rev. Mol. Diagn 9, 325–341. 10.1586/erm.09.15 19435455 PMC2701163

[B195] Luo Q. Liu X. Li Z. Huang C. Zhang W. Meng J. (2014). Degradation model of bioabsorbable cardiovascular stents. PLoS One 9, e110278. 10.1371/journal.pone.0110278 25365310 PMC4217724

[B196] Ma X.-Y. Ma T.-C. Feng Y.-F. Xiang G. Lei W. Zhou D.-P. (2021). Promotion of osteointegration under diabetic conditions by a silk fibroin coating on 3D-printed porous titanium implants via a ROS-mediated NF-κB pathway. Biomed. Mater. 16, 035015. 10.1088/1748-605X/abaaa1 32726758

[B197] Magetsari R. Dewo P. Saputro B. Lanodiyu Z. (2014). Cinnamon oil and chitosan coating on orthopaedic implant surface for prevention of Staphylococcus epidermidis biofilm formation. Malays Orthop. J. 8, 11–14. 10.5704/MOJ.1411.003 26401229 PMC4536393

[B198] Maia M. T. Luz ÉPCG Andrade F. K. Rosa M. de F. Borges M. de F. Arcanjo M. R. A. (2021). Advances in bacterial cellulose/strontium apatite composites for bone applications. Polym. Rev. 61, 736–764. 10.1080/15583724.2021.1896543

[B199] Mainier F. B. (2013). Corrosion in surgical instruments. IOSR J. Eng. 3, 25–31. 10.9790/3021-031032531

[B200] Major M. R. Wong V. W. Nelson E. R. Longaker M. T. Gurtner G. C. (2015). The foreign body response. Plast. Reconstr. Surg. 135, 1489–1498. 10.1097/PRS.0000000000001193 25919260

[B201] Manam N. S. Harun W. S. W. Shri D. N. A. Ghani S. A. C. Kurniawan T. Ismail M. H. (2017a). Study of corrosion in biocompatible metals for implants: a review. J. Alloys Compd. 701, 698–715. 10.1016/j.jallcom.2017.01.196

[B202] Manam N. S. Harun W. S. W. Shri D. N. A. Ghani S. A. C. Kurniawan T. Ismail M. H. (2017b). Study of corrosion in biocompatible metals for implants: a review. J. Alloys Compd. 701, 698–715. 10.1016/j.jallcom.2017.01.196

[B203] Mangaraj S. Priyadarshini I. Swain S. Rautray T. R. (2024). ZnO-doped β-TCP ceramic-based scaffold promotes osteogenic and antibacterial activity. Bioinspired, Biomim. Nanobiomaterials 13, 37–44. 10.1680/jbibn.23.00065

[B204] Mata M. Milian L. Oliver M. Zurriaga J. Sancho-Tello M. Llano J. J. M. de (2017). *In vivo* articular cartilage regeneration using human dental pulp stem cells cultured in an alginate scaffold: a preliminary study. Stem Cells Int. 2017, 1–9. 10.1155/2017/8309256 PMC5603743 28951745

[B205] Matschegewski C. Kohse S. Markhoff J. Teske M. Wulf K. Grabow N. (2022). Accelerated endothelialization of nanofibrous scaffolds for biomimetic cardiovascular implants. Materials 15, 2014. 10.3390/ma15062014 35329466 PMC8955317

[B206] Mattox D. M. (2010). “Atomistic film growth and some growth-related film properties,” in Handbook of physical vapor deposition (PVD) processing (Elsevier), 333–398. 10.1016/B978-0-8155-2037-5.00010-1

[B207] Mei D. Lamaka S. V. Lu X. Zheludkevich M. L. (2020). Selecting medium for corrosion testing of bioabsorbable magnesium and other metals – a critical review. Corros. Sci. 171, 108722. 10.1016/j.corsci.2020.108722

[B208] Meng M. Wang J. Huang H. Liu X. Zhang J. Li Z. (2023). 3D printing metal implants in orthopedic surgery: methods, applications and future prospects. J. Orthop. Transl. 42, 94–112. 10.1016/j.jot.2023.08.004 PMC10480061 37675040

[B209] Mezher M. AlAbbas A. R. (2022). Performance analysis of hard decision and soft decision algorithms over *in vivo* radio channel, 288–300. 10.1007/978-3-031-04112-9_22

[B210] Mezher M. Ilyas M. Bayat O. Abbasi Q. H. (2020). “Bit error rate performance of in-vivo radio channel using maximum likelihood sequence estimation,” in 2020 International Conference on Electrical, Communication, and Computer Engineering (ICECCE), Istanbul, Turkey, June 12–13, 2020 (IEEE), 1–4. 10.1109/ICECCE49384.2020.9179248

[B211] Mir M. Ahmed N. Rehman A. ur (2017). Recent applications of PLGA based nanostructures in drug delivery. Colloids Surf. B Biointerfaces 159, 217–231. 10.1016/j.colsurfb.2017.07.038 28797972

[B212] Mohammadpour M. Samadian H. Moradi N. Izadi Z. Eftekhari M. Hamidi M. (2021). Fabrication and characterization of nanocomposite hydrogel based on alginate/nano-hydroxyapatite loaded with Linum usitatissimum extract as a bone tissue engineering scaffold. Mar. Drugs 20, 20. 10.3390/md20010020 35049874 PMC8781792

[B213] Mohan Raj R. Priya P. Raj V. (2018a). Gentamicin-loaded ceramic-biopolymer dual layer coatings on the Ti with improved bioactive and corrosion resistance properties for orthopedic applications. J. Mech. Behav. Biomed. Mater 82, 299–309. 10.1016/j.jmbbm.2017.12.033 29649658

[B214] Mohan Raj R. Priya P. Raj V. (2018b). Gentamicin-loaded ceramic-biopolymer dual layer coatings on the Ti with improved bioactive and corrosion resistance properties for orthopedic applications. J. Mech. Behav. Biomed. Mater 82, 299–309. 10.1016/j.jmbbm.2017.12.033 29649658

[B215] Moroni L. de Wijn J. R. van Blitterswijk C. A. (2008). Integrating novel technologies to fabricate smart scaffolds. J. Biomater. Sci. Polym. Ed. 19, 543–572. 10.1163/156856208784089571 18419938

[B216] Mumith A. Cheong V. S. Fromme P. Coathup M. J. Blunn G. W. (2020). The effect of strontium and silicon substituted hydroxyapatite electrochemical coatings on bone ingrowth and osseointegration of selective laser sintered porous metal implants. PLoS One 15, e0227232. 10.1371/journal.pone.0227232 31923253 PMC6953817

[B217] Nagel B. Dellweg H. Gierasch L. M. (1992). Glossary for chemists of terms used in biotechnology (IUPAC Recommendations 1992). Pure Appl. Chem. 64, 143–168. 10.1351/pac199264010143

[B218] Nair M. B. Baranwal G. Vijayan P. Keyan K. S. Jayakumar R. (2015). Composite hydrogel of chitosan–poly(hydroxybutyrate-co -valerate) with chondroitin sulfate nanoparticles for nucleus pulposus tissue engineering. Colloids Surf. B Biointerfaces 136, 84–92. 10.1016/j.colsurfb.2015.08.026 26363270

[B219] Najafloo R. Baheiraei N. Imani R. (2021). Synthesis and characterization of collagen/calcium phosphate scaffolds incorporating antibacterial agent for bone tissue engineering application. J. Bioact. Compat. Polym. 36, 29–43. 10.1177/0883911520966692

[B220] Nam S. Mooney D. (2021). Polymeric tissue adhesives. Chem. Rev. 121, 11336–11384. 10.1021/acs.chemrev.0c00798 33507740

[B221] Nasar A. (2019). “Hydroxyapatite and its coatings in dental implants,” in Applications of nanocomposite materials in dentistry (Elsevier), 145–160. 10.1016/B978-0-12-813742-0.00008-0

[B222] Nathanael A. J. Oh T. H. (2020). Biopolymer coatings for biomedical applications. Polym. (Basel) 12, 3061. 10.3390/polym12123061 PMC7767366 33371349

[B223] Neugebauer J. Schoenbaum T. Pi-Anfruns J. Yang M. Lander B. Blatz M. (2023). Ceramic dental implants: a systematic review and meta-analysis. Int. J. Oral Maxillofac. Implants 38, 30–36. 10.11607/jomi.10500 37436947

[B224] Ng P. Pinho A. R. Gomes M. C. Demidov Y. Krakor E. Grume D. (2022). Fabrication of antibacterial, osteo‐inductor 3D printed aerogel‐based scaffolds by incorporation of drug laden hollow mesoporous silica microparticles into the self‐assembled silk fibroin biopolymer. Macromol. Biosci. 22, e2100442. 10.1002/mabi.202100442 35029037

[B225] Notario-Pérez F. Martín-Illana A. Cazorla-Luna R. Ruiz-Caro R. Veiga M. D. (2022). Applications of chitosan in surgical and post-surgical materials. Mar. Drugs 20, 396. 10.3390/md20060396 35736199 PMC9228111

[B226] Oliveira É. R. Nie L. Podstawczyk D. Allahbakhsh A. Ratnayake J. Brasil D. L. (2021). Advances in growth factor delivery for bone tissue engineering. Int. J. Mol. Sci. 22, 903. 10.3390/ijms22020903 33477502 PMC7831065

[B227] Oliveira J. A. M. Araujo T. G. Costa J. D. Almeida A. F. de Campos A. R. N. Santana R. A. C. de (2023). Effect of chitosan–tungsten composite coating obtained by electrophoretic deposition on the corrosion resistance of Ni–Ti alloy in physiological medium. J. Eng. Exact Sci. 9, 16237–01e. 10.18540/jcecvl9iss6pp16237-01e

[B228] Ong K. L. Yun B. M. White J. B. (2015). New biomaterials for orthopedic implants. Orthop. Res. Rev. 7, 107–130. 10.2147/ORR.S63437

[B229] Oprea T. I. Hasselgren C. (2017). “Predicting target and chemical druggability,” in Comprehensive medicinal Chemistry III (Elsevier), 429–439. 10.1016/B978-0-12-409547-2.12342-X

[B230] Pacharra S. McMahon S. Duffy P. Basnett P. Yu W. Seisel S. (2020). Cytocompatibility evaluation of a novel series of PEG-functionalized lactide-caprolactone copolymer biomaterials for cardiovascular applications. Front. Bioeng. Biotechnol. 8, 991. 10.3389/fbioe.2020.00991 32903548 PMC7438451

[B231] Pafiti K. Cui Z. Adlam D. Hoyland J. Freemont A. J. Saunders B. R. (2016). Hydrogel composites containing sacrificial collapsed hollow particles as dual action pH-responsive biomaterials. Biomacromolecules 17, 2448–2458. 10.1021/acs.biomac.6b00593 27267971

[B232] Pańtak P. Czechowska J. P. Belcarz A. Zima A. (2024). The influence of titanium and cooper on physiochemical and antibacterial properties of bioceramic-based composites for orthopaedic applications. Ceram. Int. 10.1016/j.ceramint.2024.11.102

[B233] Patel D. K. Biswas A. Maiti P. (2016). “Nanoparticle-induced phenomena in polyurethanes,” in Advances in polyurethane biomaterials (Elsevier), 171–194. 10.1016/B978-0-08-100614-6.00006-8

[B234] Păun A. G. Dumitriu C. Ungureanu C. Popescu S. (2023). Silk fibroin/ZnO coated TiO2 nanotubes for improved antimicrobial effect of Ti dental implants. Materials 16, 5855. 10.3390/ma16175855 37687548 PMC10488414

[B235] Paxton N. C. Tetsworth K. Woodruff M. A. (2023). Chapter 33 - personalization for surgical implants. In: G., Paul M. Hamdy Doweidar Editors Digital human modeling and medicine, Academic Press; p. 849–874. 10.1016/B978-0-12-823913-1.00019-1

[B236] Pei Y. Zhang G. Zhang C. Wang J. Hang R. Yao X. (2019). Corrosion resistance, anticoagulant and antibacterial properties of surface-functionalized magnesium alloys. Mater Lett. 234, 323–326. 10.1016/j.matlet.2018.09.140

[B237] Pelusi L. Mandatori D. Mastropasqua L. Agnifili L. Allegretti M. Nubile M. (2023). Innovation in the development of synthetic and natural ocular drug delivery systems for eye diseases treatment: focusing on drug-loaded ocular inserts, contacts, and intraocular lenses. Pharmaceutics 15, 625. 10.3390/pharmaceutics15020625 36839947 PMC9961328

[B238] Podgórski R. Wojasiński M. Ciach T. (2022). Nanofibrous materials affect the reaction of cytotoxicity assays. Sci. Rep. 12, 9047. 10.1038/s41598-022-13002-w 35641539 PMC9156782

[B239] Podgórski R. Wojasiński M. Trepkowska-Mejer E. Ciach T. (2023). A simple and fast method for screening production of polymer-ceramic filaments for bone implant printing using commercial fused deposition modelling 3D printers. Biomater. Adv. 146, 213317. 10.1016/j.bioadv.2023.213317 36738523

[B240] Prakash M. Audi K. Vaderhobli R. M. (2021). Long-term success of all-ceramic dental implants compared with titanium implants. J. Long. Term. Eff. Med. Implants 31, 73–89. 10.1615/JLongTermEffMedImplants.2021037400 33822537

[B241] Prasadh S. Raguraman S. Wong R. Gupta M. (2022). Current status and outlook of temporary implants (Magnesium/Zinc) in cardiovascular applications. Met. (Basel) 12, 999. 10.3390/met12060999

[B242] Prete S. Dattilo M. Patitucci F. Pezzi G. Parisi O. I. Puoci F. (2023). Natural and synthetic polymeric biomaterials for application in wound management. J. Funct. Biomater. 14, 455. 10.3390/jfb14090455 37754869 PMC10531657

[B243] Qi H. Wang K. Li M. Zhang Y. Dong K. Heise S. (2021). Co-culture of BMSCs and HUVECs with simvastatin-loaded gelatin nanosphere/chitosan coating on Mg alloy for osteogenic differentiation and vasculogenesis. Int. J. Biol. Macromol. 193, 2021–2028. 10.1016/j.ijbiomac.2021.11.032 34767883

[B244] Qin Y. Howlader M. M. R. Deen M. J. Haddara Y. M. Selvaganapathy P. R. (2014). Polymer integration for packaging of implantable sensors. Sens. Actuators B Chem. 202, 758–778. 10.1016/j.snb.2014.05.063

[B245] Qiu D. Zhou P. Kang J. Chen Z. Xu Z. Yang H. (2022). ZnO nanoparticle modified chitosan/borosilicate bioglass composite scaffold for inhibiting bacterial infection and promoting bone regeneration. Biomed. Mater. 17, 065023. 10.1088/1748-605X/ac99c5 36394277

[B246] Radice S. Liu S. Pourzal R. Laurent M. P. Wimmer M. A. (2019). Effects of bovine serum albumin and hyaluronic acid on the electrochemical response of a CoCrMo alloy to cathodic and anodic excursions. J. Bio Tribocorros 5, 104. 10.1007/s40735-019-0299-4 PMC6905392 31828005

[B247] Radmansouri M. Bahmani E. Sarikhani E. Rahmani K. Sharifianjazi F. Irani M. (2018). Doxorubicin hydrochloride - loaded electrospun chitosan/cobalt ferrite/titanium oxide nanofibers for hyperthermic tumor cell treatment and controlled drug release. Int. J. Biol. Macromol. 116, 378–384. 10.1016/j.ijbiomac.2018.04.161 29723626

[B248] Rafiq N. M. Wang W. Liew S. L. Chua C. S. Wang S. (2023). A review on multifunctional bioceramic coatings in hip implants for osteointegration enhancement. Appl. Surf. Sci. Adv. 13, 100353. 10.1016/j.apsadv.2022.100353

[B249] Rahman M. Dutta N. K. Roy C. N. (2020). Magnesium alloys with tunable interfaces as bone implant materials. Front. Bioeng. Biotechnol. 8, 564. 10.3389/fbioe.2020.00564 32587850 PMC7297987

[B250] Rakib Hasan Khan M. Shankar Hazra R. Nair G. Mohammad J. Jiang L. Reindl K. (2022). Cellulose nanofibers as Scaffold-forming materials for thin film drug delivery systems. Int. J. Pharm. 627, 122189. 10.1016/j.ijpharm.2022.122189 36100147

[B251] Rammelt S. Illert T. Bierbaum S. Scharnweber D. Zwipp H. Schneiders W. (2006). Coating of titanium implants with collagen, RGD peptide and chondroitin sulfate. Biomaterials 27, 5561–5571. 10.1016/j.biomaterials.2006.06.034 16879866

[B252] Ramskogler C. Cordero L. Warchomicka F. G. Boccaccini A. R. Sommitsch C. (2016a). Biocompatible ceramic-biopolymer coatings obtained by electrophoretic deposition on electron beam structured titanium alloy surfaces. Mater. Sci. Forum 879, 1552–1557. 10.4028/www.scientific.net/MSF.879.1552

[B253] Ramskogler C. Cordero L. Warchomicka F. G. Boccaccini A. R. Sommitsch C. (2016b). Biocompatible ceramic-biopolymer coatings obtained by electrophoretic deposition on electron beam structured titanium alloy surfaces. Mater. Sci. Forum 879, 1552–1557. 10.4028/www.scientific.net/MSF.879.1552

[B254] Ramya M. (2024). Advances in biodegradable orthopaedic implants: optimizing magnesium alloy corrosion resistance for enhanced bone repair. Biomed. Mater. and Devices. 10.1007/s44174-024-00208-x

[B255] Rastogi A. Banerjee R. (2020). Statistical optimization of bacterial cellulose production by Leifsonia soli and its physico-chemical characterization. Process Biochem. 91, 297–302. 10.1016/j.procbio.2019.12.021

[B256] Razavi M. Fathi M. Savabi O. Tayebi L. Vashaee D. (2020). Biodegradable magnesium bone implants coated with a novel bioceramic nanocomposite. Materials 13, 1315. 10.3390/ma13061315 32183231 PMC7143302

[B257] Rebelo R. Fernandes M. Fangueiro R. (2017). Biopolymers in medical implants: a brief review. Procedia Eng. 200, 236–243. 10.1016/j.proeng.2017.07.034

[B258] Resnik M. Benčina M. Levičnik E. Rawat N. Iglič A. Junkar I. (2020). Strategies for improving antimicrobial properties of stainless steel. Materials 13, 2944. 10.3390/ma13132944 32630130 PMC7372344

[B259] Reyes C. D. Petrie T. A. Burns K. L. Schwartz Z. García A. J. (2007). Biomolecular surface coating to enhance orthopaedic tissue healing and integration. Biomaterials 28, 3228–3235. 10.1016/j.biomaterials.2007.04.003 17448533 PMC2034748

[B260] Richardson J. J. Björnmalm M. Caruso F. (1979). Technology-driven layer-by-layer assembly of nanofilms. Science 2015, 348. 10.1126/science.aaa2491 25908826

[B261] Rodríguez-Contreras A. Guillem-Marti J. Lopez O. Manero J. M. Ruperez E. (2019). Antimicrobial PHAs coatings for solid and porous tantalum implants. Colloids Surf. B Biointerfaces 182, 110317. 10.1016/j.colsurfb.2019.06.047 31323450

[B262] Roi A. Roi C. Victoria Țigmeanu C. Riviș M. (2024). Composite dental implants: a future restorative approach. Dent. (Lisle). 10.5772/intechopen.114174

[B263] Ruso J. M. Messina P. V. (2017). Biopolymers for medical applications (London, United Kingdom: CRC Press). 10.1201/9781315368863

[B264] Ryu H. Seo M. Rogers J. A. (2021). Bioresorbable metals for biomedical applications: from mechanical components to electronic devices. Adv. Healthc. Mater 10, e2002236. 10.1002/adhm.202002236 33586341

[B265] Saad M. Akhtar S. Srivastava S. (2018). Composite polymer in orthopedic implants: a review. Mater Today Proc. 5, 20224–20231. 10.1016/j.matpr.2018.06.393

[B266] Saboktakin M. R. Tabatabaie R. M. Ostovarazar P. Maharramov A. Ramazanov M. A. (2012). Synthesis and characterization of modified starch hydrogels for photodynamic treatment of cancer. Int. J. Biol. Macromol. 51, 544–549. 10.1016/j.ijbiomac.2012.06.024 22732133

[B267] Sadiasa A. Kim M. S. Lee B. T. (2013). Poly(lactide-co-glycolide acid)/biphasic calcium phosphate composite coating on a porous scaffold to deliver simvastatin for bone tissue engineering. J. Drug Target 21, 719–729. 10.3109/1061186X.2013.811512 23815378

[B268] Saha S. Roy S. (2022). Metallic dental implants wear mechanisms, materials, and manufacturing processes: a literature review. Materials 16, 161. 10.3390/ma16010161 36614500 PMC9821388

[B269] Sakpal D. Gharat S. Momin M. (2022). Recent advancements in polymeric nanofibers for ophthalmic drug delivery and ophthalmic tissue engineering. Biomater. Adv. 141, 213124. 10.1016/j.bioadv.2022.213124 36148709

[B270] Salave S. Rana D. Sharma A. Bharathi K. Gupta R. Khode S. (2022). Polysaccharide based implantable drug delivery: development strategies, regulatory requirements, and future perspectives. Polysaccharides 3, 625–654. 10.3390/polysaccharides3030037

[B271] Salsabila A. Pratama A. Nurrochman A. Hermawan H. Barlian A. Prajatelistia E. (2023). Preparation of tannic acid/hyaluronic acid coating to improve the corrosion resistance of implant material based on AZ31B magnesium alloy. Met. (Basel) 13, 494. 10.3390/met13030494

[B272] Samir A. Ashour F. H. Hakim A. A. A. Bassyouni M. (2022). Recent advances in biodegradable polymers for sustainable applications. Npj Mater Degrad. 6, 68. 10.1038/s41529-022-00277-7

[B273] Sanpo N. Ang S. M. Cheang P. Khor K. A. (2009). Antibacterial property of cold sprayed chitosan-Cu/Al coating. J. Therm. Spray Technol. 18, 600–608. 10.1007/s11666-009-9391-5

[B274] Seidman M. A. Padera R. F. Schoen F. J. (2020). 2.5.2B - cardiovascular medical devices: stents, grafts, stent-grafts and other endovascular devices,” in Biomaterials science. Fourth Edition, Editors W. R. Wagner S. E. Sakiyama-Elbert G. Zhang M. J. Yaszemski (Academic Press), 1033–1050. 10.1016/B978-0-12-816137-1.00068-4

[B275] Sergi R. Bellucci D. Cannillo V. (2020). A review of bioactive glass/natural polymer composites: state of the art. Materials 13, 5560–5597. 10.3390/ma13235560 33291305 PMC7730917

[B277] Seyfoddin A. Chan A. Chen W.-T. Rupenthal I. D. Waterhouse G. I. N. Svirskis D. (2015). Electro-responsive macroporous polypyrrole scaffolds for triggered dexamethasone delivery. Eur. J. Pharm. Biopharm. 94, 419–426. 10.1016/j.ejpb.2015.06.018 26141345

[B278] Shanmugam K. Sahadevan R. (2018) “Bioceramics—an introductory overview,” in Fundamental biomaterials: ceramics (Elsevier), 1–46. 10.1016/B978-0-08-102203-0.00001-9

[B279] Sheardown H. Hicks E. A. Tayab A. Muirhead B. “2.5.6 - ophthalmologic applications: introduction,” in Biomaterials science. Fourth Edition, Editors W. R. Wagner S. E. Sakiyama-Elbert G. Zhang M. J. Yaszemski (Academic Press) 2020, 1135–1152. 10.1016/B978-0-12-816137-1.00072-6

[B280] Shen E. Wang C. Fu E. Chiang C. Chen T. Nieh S. (2008). Tetracycline release from tripolyphosphate–chitosan cross‐linked sponge: a preliminary *in vitro* study. J. Periodontal Res. 43, 642–648. 10.1111/j.1600-0765.2007.01045.x 18624950

[B281] Shen X. Shukla P. (2020) “A review of titanium based orthopaedic implants (part-I): physical characteristics,” in Problems and the need for surface modification, 1.

[B282] Shen Y. Yu X. Cui J. Yu F. Liu M. Chen Y. (2022). Development of biodegradable polymeric stents for the treatment of cardiovascular diseases. Biomolecules 12, 1245. 10.3390/biom12091245 36139086 PMC9496387

[B283] Sheng Y. Yang J. Zhao X. Liu H. Cui S. Chen L. (2020). Development and *in vitro* biodegradation of biomimetic zwitterionic phosphorylcholine chitosan coating on Zn1Mg alloy. ACS Appl. Mater Interfaces 12, 54445–54458. 10.1021/acsami.0c16662 33231070

[B284] Sherman D. Brandon D. (2008). “Mechanical properties and their relation toMicrostructure,” in Handbook ofCeramic hard materials. Editor Riedel R. 1st ed. (Darmstadt: Wiley VCH), 70.

[B285] Shi Y. Quan R. Xie S. Li Q. Cao G. Zhuang W. (2016). Evaluation of a novel HA/ZrO2-based porous bioceramic artificial vertebral body combined with a rhBMP-2/chitosan slow-release hydrogel. PLoS One 11, e0157698. 10.1371/journal.pone.0157698 27400197 PMC4939960

[B286] Shiblee MDNI Ahmed K. Khosla A. Kawakami M. Furukawa H. (2018). 3D printing of shape memory hydrogels with tunable mechanical properties. Soft Matter 14, 7809–7817. 10.1039/C8SM01156G 30074040

[B287] Silvestri A. Serafini P. M. Sartori S. Ferrando P. Boccafoschi F. Milione S. (2011). Polyurethane‐based biomaterials for shape‐adjustable cardiovascular devices. J. Appl. Polym. Sci. 122, 3661–3671. 10.1002/app.34779

[B288] Sknepnek A. Filipović S. Pavlović V. B. Mirković N. Miletić D. Gržetić J. (2024). Effects of synthesis parameters on structure and antimicrobial properties of bacterial cellulose/hydroxyapatite/TiO2 polymer–ceramic composite material. Polym. (Basel) 16, 470. 10.3390/polym16040470 PMC10892185 38399848

[B289] Song R. Murphy M. Li C. Ting K. Soo C. Zheng Z. (2018). Current development of biodegradable polymeric materials for biomedical applications. Drug Des. Devel Ther. 12, 3117–3145. 10.2147/DDDT.S165440 PMC6161720 30288019

[B290] Soran Z. Aydın R. S. T. Gümüşderelioğlu M. (2012). Chitosan scaffolds with BMP-6 loaded alginate microspheres for periodontal tissue engineering. J. Microencapsul. 29, 770–780. 10.3109/02652048.2012.686531 22612554

[B291] Sotoudeh Bagha P. Paternoster C. Khakbiz M. Sheibani S. Gholami N. Mantovani D. (2023). Surface modification of an absorbable bimodal Fe-Mn-Ag alloy by nitrogen plasma immersion ion implantation. Materials 16, 1048. 10.3390/ma16031048 36770055 PMC9919902

[B292] Stumpf T. R. Sandarage R. V. Galuta A. Fournier P. Li T. Kirkwood K. (2020). Design and evaluation of a biosynthesized cellulose drug releasing duraplasty. Mater. Sci. Eng. C 110, 110677. 10.1016/j.msec.2020.110677 32204106

[B293] Sudha P. N. Sangeetha K. Jisha Kumari A. V. Vanisri N. Rani K. (2018) “Corrosion of ceramic materials,” in Fundamental biomaterials: ceramics (Elsevier), 223–250. 10.1016/B978-0-08-102203-0.00009-3

[B294] Sun W. Liu W. Wu Z. Chen H. (2020). Chemical surface modification of polymeric biomaterials for biomedical applications. Macromol. Rapid Commun. 41, e1900430. 10.1002/marc.201900430 32134540

[B295] Sung Y. K. Kim S. W. (2020). Recent advances in polymeric drug delivery systems. Biomater. Res. 24, 12. 10.1186/s40824-020-00190-7 32537239 PMC7285724

[B296] Tabary N. Chai F. Blanchemain N. Neut C. Pauchet L. Bertini S. (2014). A chlorhexidine-loaded biodegradable cellulosic device for periodontal pockets treatment. Acta Biomater. 10, 318–329. 10.1016/j.actbio.2013.09.032 24090988

[B297] Tack L. Schickle K. Böke F. Fischer H. (2015). Immobilization of specific proteins to titanium surface using self-assembled monolayer technique. Dent. Mater. 31, 1169–1179. 10.1016/j.dental.2015.06.019 26188646

[B298] Tajvar S. Hadjizadeh A. Samandari S. S. (2023) Scaffold degradation in bone tissue engineering: an overview. Int. Biodeterior. Biodegrad. 180, 105599. 10.1016/j.ibiod.2023.105599

[B299] Tambone E. Bonomi E. Ghensi P. Maniglio D. Ceresa C. Agostinacchio F. (2021). Rhamnolipid coating reduces microbial biofilm formation on titanium implants: an *in vitro* study. BMC Oral Health 21, 49. 10.1186/s12903-021-01412-7 33541349 PMC7863462

[B300] Tan H. C. Poh C. K. Cai Y. Wang W. (2013). Anti‐fibrosis effect of BMP‐7 peptide functionalization on cobalt chromium alloy. J. Orthop. Res. 31, 983–990. 10.1002/jor.22313 23456668

[B301] Tapscott D. C. Wottowa C. (2023). Orthopedic implant materials. Treasure Island (FL): StatPearls Publishing. 32809340

[B302] Tarrahi R. Khataee A. Karimi A. Golizadeh M. Ebadi Fard Azar F. (2021). Development of a cellulose-based scaffold for sustained delivery of curcumin. Int. J. Biol. Macromol. 183, 132–144. 10.1016/j.ijbiomac.2021.04.123 33905801

[B303] Taxell P. Huuskonen P. (2022). Toxicity assessment and health hazard classification of stainless steels. Regul. Toxicol. Pharmacol. 133, 105227. 10.1016/j.yrtph.2022.105227 35817207

[B304] Teo A. J. T. Mishra A. Park I. Kim Y.-J. Park W.-T. Yoon Y.-J. (2016). Polymeric biomaterials for medical implants and devices. ACS Biomater. Sci. Eng. 2, 454–472. 10.1021/acsbiomaterials.5b00429 33465850

[B305] Terzopoulou Z. Zamboulis A. Koumentakou I. Michailidou G. Noordam M. J. Bikiaris D. N. (2022). Biocompatible synthetic polymers for tissue engineering purposes. Biomacromolecules 23, 1841–1863. 10.1021/acs.biomac.2c00047 35438479

[B306] Thiem D. G. E. Stephan D. Kniha K. Kohal R. J. Röhling S. Spies B. C. (2022a). German S3 guideline on the use of dental ceramic implants. Int. J. Implant Dent. 8, 43. 10.1186/s40729-022-00445-z 36190587 PMC9530079

[B307] Thiem D. G. E. Stephan D. Kniha K. Kohal R. J. Röhling S. Spies B. C. (2022b). German S3 guideline on the use of dental ceramic implants. Int. J. Implant Dent. 8, 43. 10.1186/s40729-022-00445-z 36190587 PMC9530079

[B308] Tomić S. Lj Babić Radić M. M. Vuković J. S. Filipović V. V. Nikodinovic-Runic J. Vukomanović M. (2023). Alginate-based hydrogels and scaffolds for biomedical applications. Mar. Drugs 21, 177. 10.3390/md21030177 36976226 PMC10055882

[B309] Topete A. Saramago B. Serro A. P. (2021). Intraocular lenses as drug delivery devices. Int. J. Pharm. 602, 120613. 10.1016/j.ijpharm.2021.120613 33865952

[B310] Trachtenberg J. E. Placone J. K. Smith B. T. Fisher J. P. Mikos A. G. (2017). Extrusion-based 3D printing of poly(propylene fumarate) scaffolds with hydroxyapatite gradients. J. Biomater. Sci. Polym. Ed. 28, 532–554. 10.1080/09205063.2017.1286184 28125380 PMC5597446

[B311] Traian C. Damien H. (2016). Biomaterials and regenerative medicine in ophthalmology. Woodhead Publishing is an imprint of Elsevier.

[B312] Tüten N. Canadinc D. Motallebzadeh A. Bal B. (2019). Microstructure and tribological properties of TiTaHfNbZr high entropy alloy coatings deposited on Ti 6Al 4V substrates. Intermet. (Barking) 105, 99–106. 10.1016/j.intermet.2018.11.015

[B313] Uppal G. Thakur A. Chauhan A. Bala S. (2022). Magnesium based implants for functional bone tissue regeneration – a review. J. Magnesium Alloys 10, 356–386. 10.1016/j.jma.2021.08.017

[B314] Vach Agocsova S. Culenova M. Birova I. Omanikova L. Moncmanova B. Danisovic L. (2023). Resorbable biomaterials used for 3D scaffolds in tissue engineering: a review. Materials 16, 4267. 10.3390/ma16124267 37374451 PMC10301242

[B315] Valente K. P. Brolo A. Suleman A. (2020). From dermal patch to implants—applications of biocomposites in living tissues. Molecules 25, 507. 10.3390/molecules25030507 31991641 PMC7037691

[B316] Vasconcelos D. M. Santos S. G. Lamghari M. Barbosa M. A. (2016). The two faces of metal ions: from implants rejection to tissue repair/regeneration. Biomaterials 84, 262–275. 10.1016/j.biomaterials.2016.01.046 26851391

[B317] Vazquez-Silva E. Abad-Farfán G. Peña-Tapia P. G. Torres-Jara P. B. Moncayo-Matute F. P. Viloria-Avila T. J. (2022). Composites and hybrid materials used for implants and bone reconstruction: a state of the art. Contemp. Eng. Sci. 15, 105–135. 10.12988/ces.2022.91974

[B318] Verma P. K. Singh S. Kapoor M. Singh S. (2024). A review on the surface topography and corrosion behavior of Mg-alloy coatings for biomedical implants. Results Surfaces Interfaces 15, 100227. 10.1016/j.rsurfi.2024.100227

[B319] Wahl D. Czernuszka J. (2006). Collagen-hydroxyapatite composites for hard tissue repair. Eur. Cell Mater 11, 43–56. 10.22203/eCM.v011a06 16568401

[B320] Wang C. Wang J. Zhang X. Yu S. Wen D. Hu Q. (2018). *In situ* formed reactive oxygen species–responsive scaffold with gemcitabine and checkpoint inhibitor for combination therapy. Sci. Transl. Med. 10, eaan3682. 10.1126/scitranslmed.aan3682 29467299

[B321] Wang S. Xu Y. Zhou J. Li H. Chang J. Huan Z. (2017). *In vitro* degradation and surface bioactivity of iron-matrix composites containing silicate-based bioceramic. Bioact. Mater 2, 10–18. 10.1016/j.bioactmat.2016.12.001 29744406 PMC5935011

[B322] Wang X. Li Y. Ren W. Hou R. Liu H. Li R. (2021a). PEI-modified diatomite/chitosan composites as bone tissue engineering scaffold for sustained release of BMP-2. J. Biomater. Sci. Polym. Ed. 32, 1337–1355. 10.1080/09205063.2021.1916868 33858302

[B323] Wang X. Li Y. Ren W. Hou R. Liu H. Li R. (2021b). PEI-modified diatomite/chitosan composites as bone tissue engineering scaffold for sustained release of BMP-2. J. Biomater. Sci. Polym. Ed. 32, 1337–1355. 10.1080/09205063.2021.1916868 33858302

[B324] Wang Y. Lan H. Yin T. Zhang X. Huang J. Fu H. (2020). Covalent immobilization of biomolecules on stent materials through mussel adhesive protein coating to form biofunctional films. Mater. Sci. Eng. C 106, 110187. 10.1016/j.msec.2019.110187 31753395

[B325] Wei W. Zhu J. Liu Y. Chen L. Zhu W. Ji H. (2024). Graphene oxide-silver-coated sulfonated polyetheretherketone (Ag/GO-SPEEK): a broad-spectrum antibacterial artificial bone implants. ACS Appl. Bio Mater 7, 3981–3990. 10.1021/acsabm.4c00338 38781457

[B326] Wells C. M. Harris M. Choi L. Murali V. P. Guerra F. D. Jennings J. A. (2019). Stimuli-responsive drug release from smart polymers. J. Funct. Biomater. 10, 34. 10.3390/jfb10030034 31370252 PMC6787590

[B327] Wong H. L. Santhanam J. Ng S. F. Bharatham B. H. (2023). Fabrication of ciprofloxacin loaded alginate/cockle shell powder nanobiocomposite bone scaffold. Life Sci. Med. Biomed. 7. 10.28916/lsmb.7.1.2023.111

[B328] Wu D. Spanou A. Diez-Escudero A. Persson C. (2020). 3D-printed PLA/HA composite structures as synthetic trabecular bone: a feasibility study using fused deposition modeling. J. Mech. Behav. Biomed. Mater 103, 103608. 10.1016/j.jmbbm.2019.103608 32090935

[B329] Wu H. Zhuo F. Qiao H. Kodumudi Venkataraman L. Zheng M. Wang S. (2022). Polymer‐/Ceramic‐based dielectric composites for energy storage and conversion. ENERGY and Environ. Mater. 5, 486–514. 10.1002/eem2.12237

[B330] Wu K. Y. Tan K. Akbar D. Choulakian M. Y. Tran S. D. (2023). A new era in ocular therapeutics: advanced drug delivery systems for uveitis and neuro-ophthalmologic conditions. Pharmaceutics 15, 1952. 10.3390/pharmaceutics15071952 37514137 PMC10385446

[B331] Wu S. Xu J. Zou L. Luo S. Yao R. Zheng B. (2021). Long-lasting renewable antibacterial porous polymeric coatings enable titanium biomaterials to prevent and treat peri-implant infection. Nat. Commun. 12, 3303. 10.1038/s41467-021-23069-0 34083518 PMC8175680

[B332] Xia D. Chen J. Zhang Z. Dong M. (2022). Emerging polymeric biomaterials and manufacturing techniques in regenerative medicine. Aggregate 3, e176. 10.1002/agt2.176

[B333] Xu L. Chu Z. Wang H. Cai L. Tu Z. Liu H. (2019a). Electrostatically assembled multilayered films of biopolymer enhanced nanocapsules for on-demand drug release. ACS Appl. Bio Mater 2, 3429–3438. 10.1021/acsabm.9b00381 35030731

[B334] Xu L. Chu Z. Wang H. Cai L. Tu Z. Liu H. (2019b). Electrostatically assembled multilayered films of biopolymer enhanced nanocapsules for on-demand drug release. ACS Appl. Bio Mater 2, 3429–3438. 10.1021/acsabm.9b00381 35030731

[B335] Xu L. Fu X. Su H. Sun H. Li R. Wan Y. (2022). Corrosion and tribocorrosion protection of AZ31B Mg alloy by a hydrothermally treated PEO/chitosan composite coating. Prog. Org. Coat. 170, 107002. 10.1016/j.porgcoat.2022.107002

[B336] Yang W. Liu Y. Pang S. Liaw P. K. Zhang T. (2020). Bio-corrosion behavior and *in vitro* biocompatibility of equimolar TiZrHfNbTa high-entropy alloy. Intermet. (Barking) 124, 106845. 10.1016/j.intermet.2020.106845

[B337] Yang X. Huang J. Chen C. Zhou L. Ren H. Sun D. (2023). Biomimetic design of double-sided functionalized silver nanoparticle/bacterial cellulose/hydroxyapatite hydrogel mesh for temporary cranioplasty. ACS Appl. Mater Interfaces 15, 10506–10519. 10.1021/acsami.2c22771 36800308

[B338] Yoon J. P. Lee C.-H. Jung J. W. Lee H.-J. Lee Y.-S. Kim J.-Y. (2018). Sustained delivery of transforming growth factor β1 by use of absorbable alginate scaffold enhances rotator cuff healing in a rabbit model. Am. J. Sports Med. 46, 1441–1450. 10.1177/0363546518757759 29543511

[B339] Yu X. Wang Y. Liu X. Ge Y. Zhang S. (2021). Ursolic acid loaded-mesoporous hydroxylapatite/chitosan therapeutic scaffolds regulate bone regeneration ability by promoting the M2-type polarization of macrophages. Int. J. Nanomedicine 16, 5301–5315. 10.2147/IJN.S323033 34393482 PMC8355748

[B340] Yu Y. Ran Q. Shen X. Zheng H. Cai K. (2020). Enzyme responsive titanium substrates with antibacterial property and osteo/angio-genic differentiation potentials. Colloids Surf. B Biointerfaces 185, 110592. 10.1016/j.colsurfb.2019.110592 31639570

[B341] Yuan Z. Ding J. Zhang Y. Huang B. Song Z. Meng X. (2022). Components, mechanisms and applications of stimuli-responsive polymer gels. Eur. Polym. J. 177, 111473. 10.1016/j.eurpolymj.2022.111473

[B342] Zafar M. S. Fareed M. A. Riaz S. Latif M. Habib S. R. Khurshid Z. (2020). Customized therapeutic surface coatings for dental implants. Coatings 10, 568. 10.3390/coatings10060568

[B343] Zaffe D. Bertoldi C. Consolo U. (2004). Accumulation of aluminium in lamellar bone after implantation of titanium plates, Ti–6Al–4V screws, hydroxyapatite granules. Biomaterials 25, 3837–3844. 10.1016/j.biomaterials.2003.10.020 15020159

[B344] Zarrintaj P. Seidi F. Youssefi Azarfam M. Khodadadi Yazdi M. Erfani A. Barani M. (2023). Biopolymer-based composites for tissue engineering applications: a basis for future opportunities. Compos B Eng. 258, 110701. 10.1016/j.compositesb.2023.110701

[B345] Zhai W. Bai L. Zhou R. Fan X. Kang G. Liu Y. (2021). Recent progress on wear‐resistant materials: designs, properties, and applications. Adv. Sci. 8, e2003739. 10.1002/advs.202003739 PMC8188226 34105292

[B346] Zhang C. Pan D. Li J. Hu J. Bains A. Guys N. (2017). Enzyme-responsive peptide dendrimer-gemcitabine conjugate as a controlled-release drug delivery vehicle with enhanced antitumor efficacy. Acta Biomater. 55, 153–162. 10.1016/j.actbio.2017.02.047 28259838

[B347] Zhang D. Xu X. Long X. Cheng K. Li J. (2019a). Advances in biomolecule inspired polymeric material decorated interfaces for biological applications. Biomater. Sci. 7, 3984–3999. 10.1039/C9BM00746F 31429424

[B348] Zhang D. Xu X. Long X. Cheng K. Li J. (2019b). Advances in biomolecule inspired polymeric material decorated interfaces for biological applications. Biomater. Sci. 7, 3984–3999. 10.1039/C9BM00746F 31429424

[B349] Zhang H. Lin X. Cao X. Wang Y. Wang J. Zhao Y. (2024). Developing natural polymers for skin wound healing. Bioact. Mater 33, 355–376. 10.1016/j.bioactmat.2023.11.012 38282639 PMC10818118

[B350] Zhang Y. Du H. Qiu Z. Liu W. Hu Y. Wang C. (2023). Enhancement of Poly-L-lactic acid stents through polydopamine coating: boosting endothelialization and suppressing inflammation. Alexandria Eng. J. 81, 525–531. 10.1016/j.aej.2023.09.024

[B351] Zhang Y. Li Z. Guo H. Wang Q. Guo B. Jiang X. (2024). A biomimetic multifunctional scaffold for infectious vertical bone augmentation. Adv. Sci. 11, e2310292. 10.1002/advs.202310292 PMC11234421 38704674

[B352] Zhang Y. Zhang M. (2002a). Calcium phosphate/chitosan composite scaffolds for controlled *in vitro* antibiotic drug release. J. Biomed. Mater Res. 62, 378–386. 10.1002/jbm.10312 12209923

[B353] Zhang Y. Zhang M. (2002b). Calcium phosphate/chitosan composite scaffolds for controlled *in vitro* antibiotic drug release. J. Biomed. Mater Res. 62, 378–386. 10.1002/jbm.10312 12209923

[B354] Zhao L. Chen S. Xie C. Liang Q. Xu D. Chen W. (2022). The fabrication of multifunctional sodium alginate scaffold incorporating ibuprofen-loaded modified PLLA microspheres based on cryogenic 3D printing. J. Biomater. Sci. Polym. Ed. 33, 1269–1288. 10.1080/09205063.2022.2049059 35235492

[B355] Zhao Y. Bai J. Xue F. Zeng R. Wang G. Chu P. K. (2023). Smart self-healing coatings on biomedical magnesium alloys: a review. Smart Mater. Manuf. 1, 100022. 10.1016/j.smmf.2023.100022

[B356] Zheng J. Zhao H. Ouyang Z. Zhou X. Kang J. Yang C. (2022). Additively-manufactured PEEK/HA porous scaffolds with excellent osteogenesis for bone tissue repairing. Compos B Eng. 232, 109508. 10.1016/j.compositesb.2021.109508

[B357] Zheng Y. Li Y. Hu X. Shen J. Guo S. (2017). Biocompatible shape memory blend for self-expandable stents with potential biomedical applications. ACS Appl. Mater Interfaces 9, 13988–13998. 10.1021/acsami.7b04808 28382821

[B358] Zhongxing L. Shaohong W. Jinlong L. Limin Z. Yuanzheng W. Haipeng G. (2021). Three-dimensional printed hydroxyapatite bone tissue engineering scaffold with antibacterial and osteogenic ability. J. Biol. Eng. 15, 21. 10.1186/s13036-021-00273-6 34372891 PMC8353754

[B359] Zhou W. Yan J. Li Y. Wang L. Jing L. Li M. (2021). Based on the synergistic effect of Mg ^2+^ and antibacterial peptides to improve the corrosion resistance, antibacterial ability and osteogenic activity of magnesium-based degradable metals. Biomater. Sci. 9, 807–825. 10.1039/D0BM01584A 33210105

